# An aspartyl protease-mediated cleavage regulates structure and function of a flavodoxin-like protein and aids oxidative stress survival

**DOI:** 10.1371/journal.ppat.1009355

**Published:** 2021-02-25

**Authors:** Anamika Battu, Rajaram Purushotham, Partha Dey, S. Surya Vamshi, Rupinder Kaur

**Affiliations:** 1 Laboratory of Fungal Pathogenesis, Centre for DNA Fingerprinting and Diagnostics, Hyderabad, India; 2 Graduate studies, Manipal Academy of Higher Education, Manipal, Karnataka, India; University at Buffalo, UNITED STATES

## Abstract

A family of eleven glycosylphosphatidylinositol-anchored aspartyl proteases, commonly referred to as CgYapsins, regulate a myriad of cellular processes in the pathogenic yeast *Candida glabrata*, but their protein targets are largely unknown. Here, using the immunoprecipitation-mass spectrometry approach, we identify the flavodoxin-like protein (Fld-LP), CgPst2, to be an interactor of one of the aspartyl protease CgYps1. We also report the presence of four Fld-LPs in *C*. *glabrata*, which are required for survival in kidneys in the murine model of systemic candidiasis. We further demonstrated that of four Fld-LPs, CgPst2 was solely required for menadione detoxification. CgPst2 was found to form homo-oligomers, and contribute to cellular NADH:quinone oxidoreductase activity. CgYps1 cleaved CgPst2 at the C-terminus, and this cleavage was pivotal to oligomerization, activity and function of CgPst2. The arginine-174 residue in CgPst2 was essential for CgYps1-mediated cleavage, with alanine substitution of the arginine-174 residue also leading to elevated activity and oligomerization of CgPst2. Finally, we demonstrate that menadione treatment led to increased CgPst2 and CgYps1 protein levels, diminished CgYps1-CgPst2 interaction, and enhanced CgPst2 cleavage and activity, thereby implicating CgYps1 in activating CgPst2. Altogether, our findings of proteolytic cleavage as a key regulatory determinant of CgPst2, which belongs to the family of highly conserved, electron-carrier flavodoxin-fold-containing proteins, constituting cellular oxidative stress defense system in diverse organisms, unveil a hidden regulatory layer of environmental stress response mechanisms.

## Author summary

Fungal bloodstream infections in immunodeficient patients are a serious clinical problem. Infections caused by the opportunistic fungal pathogen *Candida glabrata* are difficult to treat owing to its low susceptibility to widely used azole antifungals and emerging co-resistance to azole and echinocandin drugs. Despite encountering oxidative stress in host macrophages, *C*. *glabrata* is able to replicate intracellularly. Here, we show for the first time that CgYps1 aspartyl protease-mediated cleavage regulates the structural state, activity and functions of a flavodoxin-like protein CgPst2, which belongs to the family of highly conserved electron-carrier proteins that are pivotal to oxidative stress response in organisms ranging from bacteria to humans. We also show that CgPst2 is required for oxidative stress survival *in vitro*, and is sufficient to rescue, *in vivo*, the attenuated survival of a *C*. *glabrata* mutant lacking four flavodoxin-like proteins. Further, our data suggest that CgYps1 protease-mediated cleavage of CgPst2 is a dynamic event which may be coupled closely with intracellular quinone accumulation and detoxification. This strategy may aid *C*. *glabrata* establish successful infections in the human host. While advancing our understanding of fungal pathogenesis, our findings also provide insights into hitherto unknown regulatory mechanisms for flavodoxin-like fold-containing proteins.

## Introduction

Opportunistic invasive mycoses pose a serious threat to global economy and human health [1]. *Candida* species are the most prevalent cause of life-threatening fungal bloodstream infections, that are associated with a mortality rate of up to 72% [2–5]. Although *C*. *albicans* is still the most frequently isolated *Candida spp*., the incidence of bloodstream infections due to *non-albicans Candida spp*. has increased substantially in last two decades [1,2,4,6]. *C*. *glabrata* is the second to fourth most common *Candida* bloodstream pathogen depending upon the geographical region, and accounts for up to 30% of *Candida* bloodstream infections [3,5–8]. *C*. *glabrata* differs from other prevalent pathogenic *Candida* species in several respects including its haploid genome, lack of secreted proteolytic activity and hyphae formation, and close phylogenetic relationship with the non-pathogenic yeast *Saccharomyces cerevisiae* [9–11].

A family of eleven putative glycosylphosphatidylinositol (GPI)-linked aspartyl proteases, commonly referred to as yapsins, plays an essential role in virulence of *C*. *glabrata* [12]. Other pathogenic traits of *C*. *glabrata* include the ability to adhere to biotic and abiotic surfaces probably owing to the presence of a large number of cell surface adhesins, survive high level of diverse stresses, proliferate in host macrophages and remodel chromatin and metabolic pathways in response to environmental cues [10,11]. Of eleven *CgYPS1-11* genes encoding CgYapsins, *CgYPS1* is essential for survival of acid stress, maintenance of pH and vacuole homeostasis, biofilm formation and survival in macrophage and mouse models [12–15]. CgYapsins are also required for suppression of the host innate immune response [15]. Intracellular proliferation of *C*. *glabrata* in macrophages is dependent upon its ability to limit activation of the NLRP3 inflammasome and Syk (spleen tyrosine kinase) signaling pathways [15]. The lack of CgYapsins resulted in higher activation of, and consequent increased production of the pro-inflammatory cytokine IL-1β in human macrophages, which led to intracellular killing of the *Cgyps1-11Δ* mutant [15]. Importantly, CgYps1 expression restored the intracellular survival and replication defect of the *Cgyps1-11Δ* mutant, pointing towards a pivotal role for CgYps1 in interaction with host immune cells [15]. However, despite the centrality of CgYapsins to *C*. *glabrata* physiology and virulence, their target proteins remain largely unidentified.

A characteristic trait of *C*. *glabrata* is the capacity to survive well under oxidative environmental conditions [11]. *C*. *glabrata* is known to exhibit about 3- and 10- fold higher resistance to hydrogen peroxide compared to *C*. *albicans* and *S*. *cerevisiae*, respectively [10,16]. Flavodoxins are highly conserved electron-carrier proteins which are implicated in oxidative stress response in bacteria and algae [17,18]. These proteins convert quinone to hydroquinone through two-electron reduction, thereby bypassing formation of the unstable and reactive semi-quinone species [17]. Flavodoxins possess a characteristic flavodoxin-like α-β-α fold with a twisted β-sheet, made of five parallel β-strands, surrounded by five α-helices, and require FMN (flavin mononucleotide) or FAD (flavin adenine dinucleotide) as cofactors for their redox activity [17,19,20]. Eukaryotes have a large number of proteins with flavodoxin-type fold domain [17,19,21]. *S*. *cerevisiae* and *C*. *albicans* contain three and four flavodoxin-like proteins (Fld-LPs), respectively, which are required for survival of the oxidative environment [22–25]. *C*. *albicans* Fld-LPs are also important for virulence in mice [25].

In the current study, we characterize, through phenotypic analysis, four Fld-LPs in *C*. *glabrata*, and show that CgPst2 is uniquely required for survival of menadione (MD) and benzoquinone (BQ) stress. Further, we identify, through immunoprecipitation and mass spectrometry analysis, 19 interactors, including CgPst2, of the CgYps1 protease, and demonstrate that CgPst2 undergoes CgYps1-dependent cleavage at the C-terminus. Arginine-174 in CgPst2 was found to be essential for cleavage, with C-terminally cleaved CgPst2 forming homo-oligomers and possessing higher activity. Altogether, our data unravel the hitherto unknown mechanism by which *C*. *glabrata* responds to, and, survive an oxidative environment by regulating the activity and structural state of a Fld-LP via proteolytic cleavage.

## Results

### CgPst2 is required for survival of MD and BQ stress

**Fig 1:**
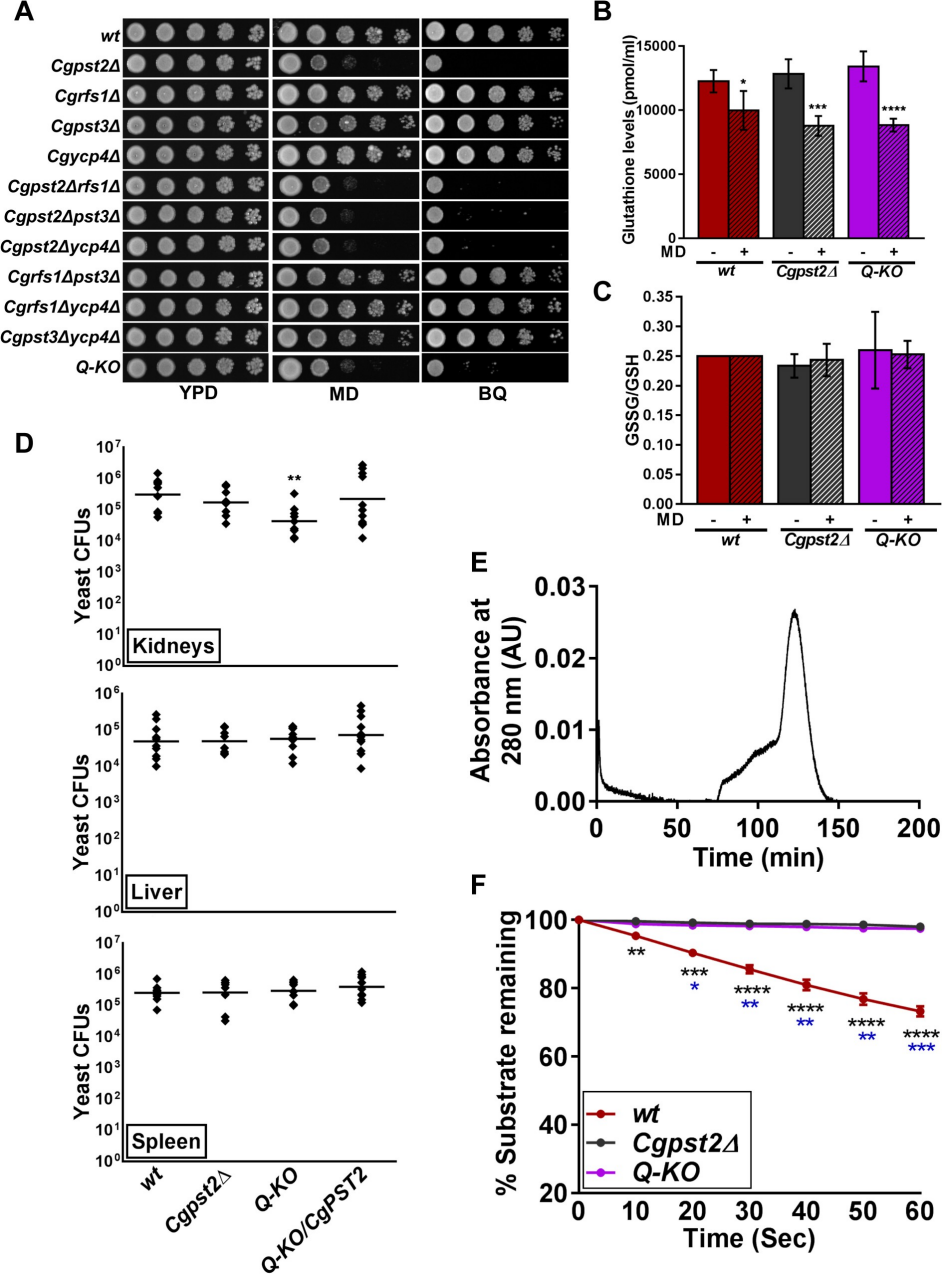
The flavodoxin-like protein CgPst2 plays an essential role in menadione and benzoquinone resistance. **A.** Serial dilution spotting assay illustrating MD (95 μM) and BQ (4 mM) sensitivity of indicated *C*. *glabrata* mutants. The *Q-KO* strain lacks four flavodoxin-like proteins, CgPst2, CgRfs1, CgPst3 and CgYcp4. **B.** Total intracellular glutathione content of log-phase cells (10.0 OD_600_). Data (mean ± SEM; n = 4–5) represent total glutathione concentration normalized to one ml of cell lysate. *, p ≤ 0.05, ***, p ≤ 0.001, ****, p ≤ 0.0001; unpaired two-tailed Student’s t test. **C.** Ratio of oxidized (GSSG) to reduced (GSH) glutathione. 4-vinylpyridine was used to derivatize/block all free thiols in cell lysates, followed by GSSG estimation using DTNB [5,5-dithio-bis-(2-nitrobenzoic acid); Ellman’s Reagent]. Reduced GSH levels were calculated by subtracting GSSG levels from total glutathione levels. Data represent (mean ± SEM; n = 3–5). **D.** Survival of indicated *C*. *glabrata* strains in the murine model of systemic candidiasis. Six to eight-week-old female BALB/c mice were infected with cells (4X10^7^ cells; 100 μl cell suspension) of overnight YPD/CAA-grown *C*. *glabrata* strains, *wt*, *Cgpst2Δ*, *Q-KO* and *Q-KO* expressing *CgPST2*, through tail vein injections. At 7^th^ day post infection, mice were sacrificed, target organs (kidneys, liver and spleen) collected, homogenized in sterile PBS and appropriate homogenate dilutions were plated on YPD medium containing penicillin and streptomycin antibiotics. Total fungal burden in each mouse organ was determined, and plotted. Diamonds and bars indicate CFUs recovered from an individual mouse, and CFU geometric mean (n = 8–12), respectively, for each organ. **, p < 0.01; Mann-Whitney U-test. **E.** Size exclusion chromatogram of purified CgPst2 protein. 300 μg 6XHIS-FLAG-CgPst2 was applied to a Sephacryl S-200 column, and protein elution profiles were determined using the absorbance values at 280 nm. The peak corresponds to 29 kDa CgPst2 protein. AU, Arbitrary Units. **F.** NADH:quinone oxidoreductase activity in cell lysates (500 μg) of log-phase *wt*, *Cgpst2****Δ*** and *Q-KO* cells, as measured using menadione (500 μM) and NADH (500 μM) substrates. Absorbance of the substrate NADH was considered as 100 at 0 h time point, and NADH oxidation was deduced from the formula: [(absorbance at each time point/0 h absorbance) X 100]. Data represent mean ± SEM. Black and blue asterisks indicate statistically significant differences in activity between *wt* and *Cgpst2Δ*, and *wt* and *Q-KO* strains, respectively. *, p < 0.0332; **, p < 0.0021; ***, p < 0.0002; ****, p < 0.0001; Grouped multiple *t*-test (n = 3 to 4).

Towards deciphering the functions of Fld-LPs in *C*. *glabrata*, we first identified, using BLASTP analysis, orthologs of *S*. *cerevisiae* [22] and *C*. *albicans* [25] Fld-LPs, and found four *C*. *glabrata* proteins, CgPst2, CgRfs1, CgPst3 and CgYcp4, to exhibit varied levels of sequence similarity with one another, and with their *S*. *cerevisiae* and *C*. *albicans* counterparts (S1A Fig). CgPst2, CgRfs1, CgPst3 and CgYcp4 all possessed FMN-binding sites (S1B Fig). Notably, *CgPST2* and *CgRFS1* ORFs were most similar, sharing 75% identity at the nucleotide level. Next, we generated strains deleted for single, double or multiple *Fld-LP* genes and profiled the created mutants phenotypically. We found growth of the *Cgpst2Δ* mutant to be highly attenuated in the presence of MD and BQ (Fig 1A). Of note, sensitivity to other oxidative stressors, duroquinone, and cumene hydroperoxide, and thermal stress was not observed for any mutant (S2A Fig). Notably, all mutants, *Cgpst2Δ* single, *Cgpst2Δrfs1Δ*, *Cgpst2Δpst3Δ* and *Cgpst2Δycp4Δ* double, and the quadruple knockout lacking all four Fld-LPs (CgPst2, CgRfs1, CgPst3 and CgYcp4; referred as *Q-KO* from hereon), showed similar growth attenuation in MD and BQ-containing medium, indicating that of four Fld-LPs, CgPst2 is the sole regulator of MD and BQ stress survival (Fig 1A). Consistently, ectopic expression of *CgPST2* complemented the quinone sensitivity of *Cgpst2Δ* and *Q-KO* strains (S2B Fig).

Menadione (2-methyl-l,4-naphthoquinone; vitamin K3), induces oxidative stress in cells, mainly due to superoxide radical and hydrogen peroxide production through the process of oxygen-dependent redox cycling [26]. Therefore, we next checked the effect of CgPst2 loss and MD treatment on intracellular levels of the antioxidant glutathione. We found similar levels of total glutathione, with unchanged GSSG/GSH ratio, in all three strains, *wt*, *Cgpst2Δ* and *Q-KO* (Fig 1B and 1C). Further, MD treatment also led to similar 20–35% reduction in total intracellular glutathione content in all strains, without any alteration in GSSG/GSH ratio (Fig 1B and 1C). These data suggest that neither CgFld-LP loss nor MD treatment had any appreciable impact on glutathione redox status.

To examine the role of Fld-LPs in pathogenesis of *C*. *glabrata*, we infected *wt*, *Cgpst2Δ* and four-Fld-LP-deficient (*Q-KO*) strains to BALB/c mice. We found *Q-KO* to exhibit attenuated survival, compared to *wt* cells, in kidneys of infected mice, which was rescued upon ectopic expression of *CgPST2* (Fig 1D), indicating that CgPst2 expression is sufficient to rescue the mutant survival defect. Further, despite strong MD and BQ sensitivity *in vitro*, *Cgpst2Δ* mutant exhibited *wt*-like virulence in mice (Fig 1D), which could reflect functional redundancy among CgFld-LPs *in vivo*. Collectively, these data suggest that CgPst2 is required for survival of MD and BQ stress *in vitro*, and CgFld-LPs modulate organ-dependent survival in the murine systemic candidiasis model.

### CgPst2 contributes to cellular NADH:quinone oxidoreductase activity

Similar to other Fld-LPs, CgPst2 is predicted to contain five-stranded parallel beta sheet surrounded by five alpha helices. WrbA, the founding member of Fld-LP family in *Escherichia coli*, and *S*. *cerevisiae* Pst2 are known to exist in a tetrameric form, and implicated in quinone detoxification [27–30]. Therefore, to determine if CgPst2 also forms a tetramer, we tagged CgPst2 with 6X-Histidine-FLAG epitope at the N-terminus, and purified the recombinant protein from *E*. *coli* (S3A Fig). Size exclusion chromatography (SEC) revealed CgPst2 to be a monomeric protein of 29 kDa (Fig 1E). The recombinant CgPst2 was non-functional, as it showed no NADH:quinone oxidoreductase activity *in vitro* (S3B Fig). Therefore, to examine if CgPst2 is a bonafide quinone-oxidoreductase, we checked the NADH:quinone oxidoreductase activity in cell extracts of *wt* and *CgPST2*-deleted strains. We monitored oxidation of NADH spectrophotometrically, and found 30% of total NADH to be oxidized within 1 min of incubation with *wt* cell extracts, while this oxidation was absent in *Cgpst2Δ* and *Q-KO* extract-incubated reaction mixtures (Fig 1F). Of note, the exogenous addition of FAD or FMN to the enzyme assay mixture had no effect on NADH:quinone oxidoreductase activity in *wt* and mutant cell extracts (S3C Fig), indicating that cell lysates have adequate amount of these cofactors. Ectopic expression of *CgPST2* in *Cgpst2Δ* and *Q-KO* strains rescued the NADH oxidation defect (S3D Fig), suggesting that no NADH:quinone oxidoreductase activity in mutant cell extracts is due to the lack of CgPst2. Collectively, these data show that CgPst2 possesses NADH:quinone oxidoreductase activity, which may play a part in MD and BQ stress survival.

### Immunoprecipitation (IP) and mass spectrometry (MS) analysis identified CgPst2 as a CgYps1 interactor

**Fig 2:**
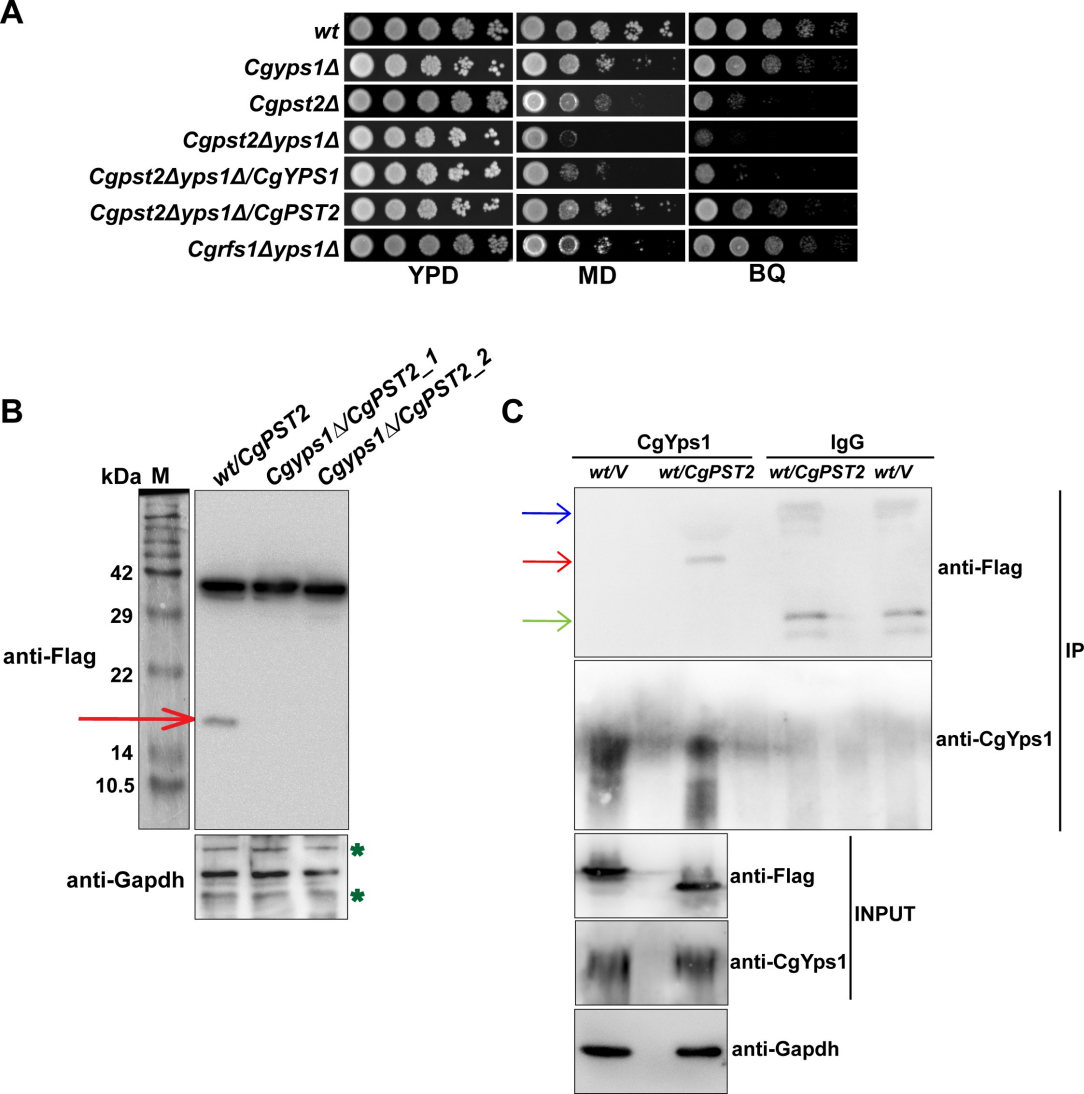
CgPst2 interacts with CgYps1. **A.** Serial dilution spotting assay illustrating MD (95 μM) and BQ (4 mM) sensitivity of indicated *CgYPS1*-deleted strains. **B.** Immunoblot analysis of CgPst2-SFB in cell extracts (60 μg) of *wt* and *Cgyps1Δ* transformants expressing *CgPST2-SFB*. The red arrow marks the small (~ 16 kDa) cleaved fragment of CgPst2. *Cgyps1Δ/CgPST2_1* and *Cgyps1Δ/CgPST2_2* represent two independent *Cgyps1Δ* transformants. M, Protein Marker. **C.** Immunoblot analysis of CgPst2-CgYps1 interaction. Lysates (3.0 mg) of *wt* cells expressing either SFB-GFP or CgPst2-SFB were immunoprecipitated with anti-CgYps1 or anti-mouse immunoglobulin G (IgG) antibody, resolved on 4–20% polyacrylamide gradient, 12% polyacrylamide and 12% polyacrylamide gels for CgYps1, CgPst2-SFB and CgGapdh, respectively, and were probed with anti-CgYps1, anti-Flag and anti-Gapdh antibodies. CgGapdh was used as loading control. Please note that for Input samples, 200, 75 and 75 μg protein was loaded for detection of CgYps1, CgPst2-SFB and CgGapdh proteins, respectively. Red, blue and green arrows mark the CgPst2-SFB, Ig heavy chain and Ig light chain, respectively. ‘*V*’ refers to the vector expressing SFB-GFP protein.

During the course of this study, we found that, compared to *C*. *albicans* and *S*. *cerevisiae*, CgPst2 was uniquely found in the secretome of *C*. *glabrata* [31]. Additionally, its secretion was enhanced in the *Cgyps1-11*Δ mutant, that lacks all eleven CgYapsins [31], indicating a role of CgYapsins in proper sorting/localization of CgPst2. Since CgYps1 has previously been implicated in MD tolerance [15], and the *Cgyps1*Δ mutant contained high reactive oxygen species (ROS) levels [14], we reasoned that CgPst2 and CgYps1 may act in conjunction during MD stress response. Thus, to decipher the relationship, if any, between CgPst2 and CgYps1, we performed three experiments. First, we generated a double mutant that lacked both CgPst2 and CgYps1. The *Cgpst2Δyps1Δ* mutant displayed enhanced sensitivity to MD, compared to single *Cgpst2Δ* and *Cgyps1Δ* mutants (Fig 2A). As a control, we also generated *Cgrfs1Δyps1Δ* mutant, and found its MD sensitivity to be similar to that of *Cgyps1Δ* mutant (Fig 2A). Further, ectopic expression of *CgYPS1* or *CgPST2* restored MD sensitivity slightly and substantially, respectively, of the *Cgpst2Δyps1Δ* mutant (Fig 2A). Expectedly, the *Cgpst2Δyps1Δ* mutant showed no NADH:quinone oxidoreductase activity (S4 Fig). Altogether, these results highlight a major and minor role for CgPst2 and CgYps1, respectively, in MD stress survival.

Second, we generated a fusion protein by tagging CgPst2 with GFP at the C-terminus. Confocal imaging revealed the fusion protein to localize at the plasma membrane in both *wt* and *Cgyps1Δ*, and MD treatment did not alter CgPst2 localization in either strain (S5 Fig). These results preclude any role of CgYps1 in cellular localization of CgPst2.

Third, we identified interactors/probable substrates of CgYps1 protease through IP-MS analysis. For this, we immunoprecipitated CgYps1, using anti-CgYps1 polyclonal antibody [31], from log-phase *wt* and *Cgyps1-11Δ* cells, in duplicates, and sent to Taplin Mass Spectrometry Facility for protein identification. By employing three criteria, (a minimum of two unique peptides, presence in both *wt* replicate samples, and absence in both *Cgyps1-11Δ* samples), we identified 19 proteins, including CgPst2, that interacted with CgYps1 (Table 1). The functional classification and predicted localization of CgYps1 interactors is listed in Table 1. Gene Ontology (GO) analysis revealed CgYps1 interactors to primarily belong to carbohydrate metabolism, response to chemical, translation, transport and anaerobic respiration processes (S1 Table), underscoring the role of CgYps1 in regulation of diverse cellular processes. Of note, this is the first report of a fungal yapsin interacting with metabolic proteins. However, since IP-MS-based analysis can produce false positives, further validation is required to establish these highly abundant cellular proteins as bonafide CgYps1 interactors.

**Table 1:** List of CgYps1 interacting proteins, as identified by immunoprecipitation-mass spectrometry analysis.

Sl. No	Interactor-encoding ORF (Systematic ID)	*S*. *cerevisiae* ortholog	Number of unique peptides (in two replicate samples)		Predicted functions^¶^	Protein size (kDa)	Predicted localization^#^
**Oxidoreduction process and energy homeostasis**							
1	*CAGL0A00495g*	*PMA1*	16	2	Plasma membrane P2-type H^+^-ATPase; a major regulator of cytoplasmic pH and plasma membrane potential.	98.3	Endoplasmic reticulum, Membrane type
2	*CAGL0J00451g*	*TDH3*	6	5	Putative glyceraldehyde-3-phosphate dehydrogenase.	35.9	Cytoplasm
3	*CAGL0D01298g*	*TKL1*	4	7	Transketolase; catalyzes conversion of xylulose-5-phosphate and ribose-5-phosphate to sedoheptulose-7-phosphate and glyceraldehyde-3-phosphate in the pentose phosphate pathway.	73.7	Peroxisome
4	*CAGL0M09581g*	*ATP1*	5	5	Alpha subunit of the F1 sector of mitochondrial F1F0 ATP synthase.	58.5	Mitochondrion
5	*CAGL0F01947g*	*LPD1*	3	5	Dihydrolipoamide dehydrogenase; the lipoamide dehydrogenase component (E3) of the pyruvate dehydrogenase and 2-oxoglutarate dehydrogenase multi-enzyme complexes.	53.1	Mitochondrion
6	*CAGL0F04213g*	*PET9*	2	5	Major ADP/ATP carrier of the mitochondrial inner membrane; exchanges cytosolic ADP for mitochondrially synthesized ATP.	32.8	Mitochondrion, Membrane type
7	*CAGL0H08327g*	*TPI1*	4	2	Triose phosphate isomerase, abundant glycolytic enzyme.	26.9	Cytoplasm
8	*CAGL0C02343g*	*ARB1*	3	3	ATPase of the ATP-binding cassette (ABC) family.	81.1	Plastid
9	*CAGL0M13343g*	*GND1*	3	3	6-phosphogluconate dehydrogenase; catalyzes an NADPH regenerating reaction in the pentose phosphate pathway.	53.5	Cytoplasm
10	*CAGL0K11858g*	*PST2*	2	2	Protein with similarity to a family of flavodoxin-like proteins; induced by oxidative stress.	21.0	Cytoplasm
11	*CAGL0I03916g*	*ARF2*	2	2	Putative ADP-ribosylation factor; involved in invasive growth.	20.6	Golgi apparatus, Membrane type
**Ribosome biogenesis**							
12	*CAGL0J07238g*	*RPS12*	3	2	Protein component of the small (40S) ribosomal subunit.	15.5	Cytoplasm
13	*CAGL0K11748g*	*RPS11A*	2	2	Protein component of the small (40S) ribosomal subunit.	17.9	Cytoplasm
14	*CAGL0L04840g*	*RPS23*	2	2	Putative ribosomal protein.	16.0	Cytoplasm
15	*CAGL0A00979g*	*RPL38*	2	2	60S ribosomal ribosomal protein subunit.	8.9	Cytoplasm
**Other functions**							
16	*CAGL0I04356g*	*TIF1*	3	2	Translation initiation factor eIF4A.	44.8	Nucleus
17	*CAGL0C04389g*	*HTB2*	3	2	Histone H2B; core histone protein required for chromatin assembly	14.0	Nucleus
18	*CAGL0H02057g*	*GAR1*	2	3	Protein component of the H/ACA snoRNP pseudouridylase complex; modify 18S pre-rRNA.	19.5	Nucleus
19	*CAGL0D01034g*	*PSA1*	2	2	GDP-mannose pyrophosphorylase involved in the synthesis of GDP-mannose for protein glycosylation	39.4	Cytoplasm

^¶^These functions are predicted based on functions of the corresponding *S*. *cerevisiae* orthologs (www.yeastgenome.org).

^#^Subcellular localization of proteins was determined using DeepLoc server (http://www.cbs.dtu.dk/services/DeepLoc/).

### CgYps1 cleaves CgPst2 at the C-terminus

Of 19 identified interactors, one protein was CgPst2. To characterize CgPst2-CgYps1 interaction, we tagged CgPst2 with SFB epitope at the C-terminus. Immunoblot analysis revealed full length CgPst2-SFB protein (~ 40 kDa) in both *wt* and *Cgyps1Δ* strains, however, a shorter fragment of ~ 16 kDa was observed uniquely in *wt* cells (Fig 2B). Its absence in *Cgyps1Δ* mutant indicates that CgYps1 may be required for CgPst2 cleavage at the C-terminus. We also validated CgPst2-CgYps1 interaction through immunoblot (Fig 2C) and affinity purification analysis (S6 Fig). Of note, we could not check the processing of CgPst2-GFP, as multiple bands were detected by anti-GFP antibody in the Western analysis.

**Fig 3:**
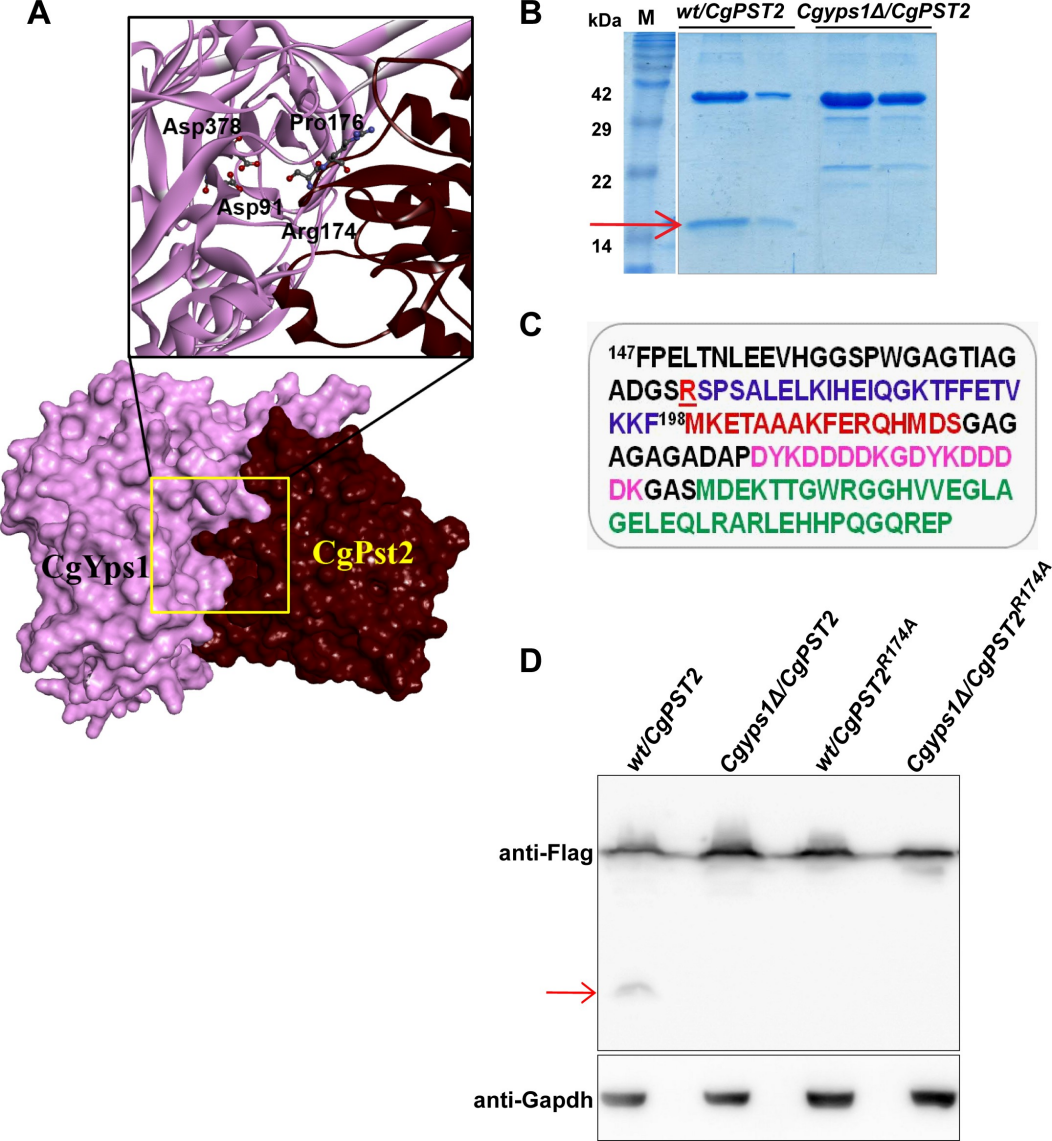
CgPst2 is cleaved at the C-terminus. **A.** Molecular surface and Ribbon models depicting CgYps1 (Length: 601 aa; Predicted MW: 64 kDa) with CgPst2 (Length: 198 aa; Predicted MW: 21 kDa). D^91^ and D^378^ in CgYps1 represent catalytically active aspartic acid residues. The ^171^DGS**R**S**P**SA^178^ region in CgPst2 contains predicted CgYps1-binding residues, Arginine-174 and Proline-176. Hydrogen bond-forming amino acid residues are highlighted in bold letters in the predicted interaction region. The molecular surfaces of receptor (CgYps1) and substrate (CgPst2) are coloured purple and maroon, respectively. **B.** Coomassie-blue-stained 18% SDS-PAGE gel images indicating expression of the full-length (~ 40 kDa) and the C-terminal cleaved fragment (~ 16 kDa; marked with a red arrow) of CgPst2-SFB, after two-step affinity purification of cell extracts of *wt* and *Cgyps1Δ* strains. **C.** The C-terminus amino acid sequence of CgPst2-SFB protein starting with the phenylalanine (F) residue at 147^th^ position of CgPst2 protein. CgPst2 is 198 amino acid long. The predicted cleavage residue R174 is underlined and indicated in red. The SFB tag (85 aa) consists of S-protein (MKETAAAKFERQHMDS; brown), two copies of the FLAG tag (DYKDDDDK; pink) and streptavidin-binding-peptide (MDEKTTGWRGGHVVEGLAGELEQLRARLEHHPQGQREP; green) sequences. MS analysis of the shorter CgPst2-SFB fragment identified peptides corresponding to SFB and CgPst2 sequences. **D.** Immunoblot analysis of *wt* cell extracts expressing either *wild-type* CgPst2-SFB or alanine-substituted-CgPst2-SFB (*CgPST2*^*R174A*^), using anti-Flag antibody. The red arrow marks the small cleaved fragment of *wild-type* CgPst2. CgGapdh was used as a loading control.

Next, we performed molecular docking analysis to predict the potential CgYps1-cleavage sites on CgPst2. For this, CgPst2 and CgYps1 sequences were retrieved from the Candida genome database, and submitted to the online tool I-TASSER [32]. The models with best Confidence (C)-score were selected and analyzed by Z-DOCK [33]. This structural modelling analysis predicted Aspartate-91 and Aspartate-378 of CgYps1 to interact with Proline-176 and Arginine-174 residues, present at the C-terminus of CgPst2, respectively (Fig 3A). The predicted arginine-174 residue lies in a highly conserved DGSR sequence in CgPst2, that CgPst2 shares with several Fld-LPs (S7 Fig). Importantly, D91 and D378, predicted catalytic residues of CgYps1, are required for its functions in cell wall stress [15]. Since Yapsins are known to cleave C-terminal to monobasic or dibasic residues, with *S*. *cerevisiae* Yps1 also reported to cleave at the monobasic residue [34,35], we hypothesized that CgYps1 may cleave CgPst2 at/after R174 residue. To test this, we extracted the ~16 kDa cleaved fragment of CgPst2-SFB (Fig 3B) and analyzed by peptide mass fingerprinting. We identified a peptide corresponding to the terminal sequence of CgPst2, ^175^SPSALELKIHEIQGKTFFETVK^196^ (Fig 3C), along with SFB epitope peptides (Fig 3C). Further, mutation of R174 in CgPst2 to alanine-174 (CgPst2^*R174A*^) led to disappearance of the shorter CgPst2-SFB fragment in CgPst2^*R174A*^-SFB*-*expressing *wt* (Fig 3D) and *Cgpst2Δ* (S8 Fig) samples, suggesting that R174 in CgPst2 is required for its cleavage at C-terminus. Of note, since we were unable to obtain good expression of N-terminally-SFB-tagged CgPst2 protein in *C*. *glabrata*, we could not use SFB-CgPst2 for cleavage analysis. Altogether, these data point towards CgPst2 being a bonafide substrate of CgYps1.

### MD treatment increases NADH:quinone oxidoreductase activity

**Fig 4:**
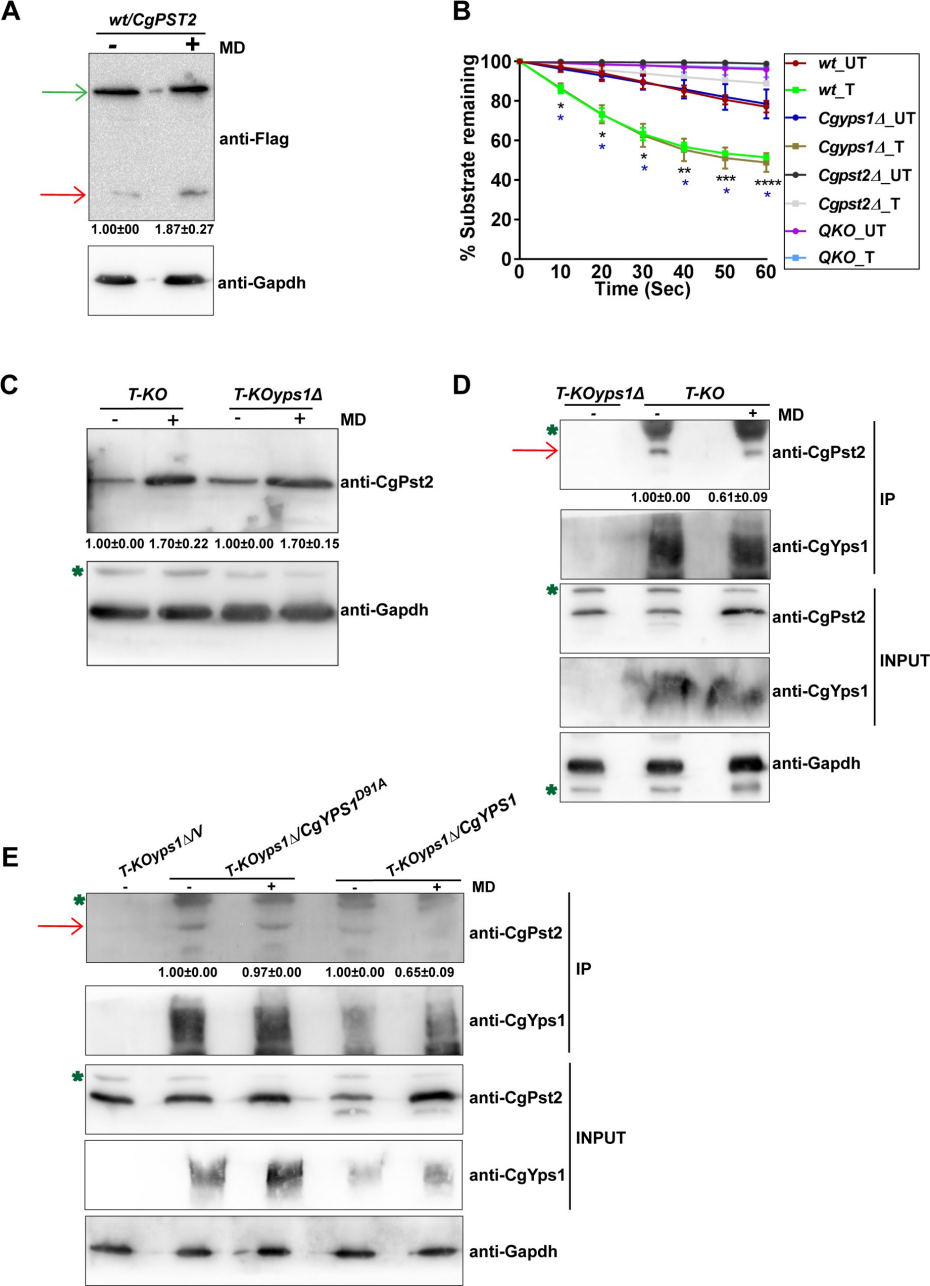
The cleavage and NADH:quinone oxidoreductase activity of CgPst2 is increased upon menadione treatment. **A.** Immunoblot analysis of CgPst2 expression, using anti-Flag antibody, in cell extracts of untreated or menadione-(MD; 90 μM for 90 min)-treated log-phase cells of the *wt* strain expressing CgPst2-SFB, using anti-Flag antibody. CgGapdh was used as a loading control. The intensity of the shorter 16 kDa band in 4 independent Western blots was quantified using the ImageJ densitometry software, and this signal was normalized to the corresponding CgGapdh-normalized total CgPst2 signal, as total CgPst2 levels were also elevated, upon MD treatment, compared to CgGapdh signal. Data (mean ± SEM) represent the fold-change in levels of the C-terminal cleaved fragment of CgPst2-SFB in treated cells, compared to untreated cells (considered as 1.0), and are shown underneath the blot. *p* ≤ 0.05; paired two-tailed Student’s *t* test. The red and green arrows indicate the C-terminal cleaved fragment of CgPst2 and full length CgPst2-SFB, respectively. **B.** NADH:quinone oxidoreductase activity in log-phase cultures of indicated *C*. *glabrata* strains that were either treated with 90 μM menadione (T) for 90 min, or left untreated (UT), as measured using menadione (500 μM) and NADH (500 μM) substrates. Absorbance of the substrate NADH was considered as 100 at 0 h time point, and NADH oxidation was deduced from the formula: [(absorbance at each time point/0 h absorbance) X 100]. Black and blue asterisks indicate statistically significant differences in activity of treated *wt* and *Cgyps1Δ* samples, respectively, compared to respective untreated lysates. The *Q-KO* strain lacks four flavodoxin-like proteins, CgPst2, CgRfs1, CgPst3 and CgYcp4. *, p < 0.0332; **, p < 0.0021; ***, p < 0.0002; ****, p < 0.0001; Grouped multiple *t*-test (n = 3 to 5). **C.** Immunoblot analysis of CgPst2 levels in *T-KO* (Cg*rfs1Δpst3Δycp4Δ*) and *T-KOyps1Δ* (Cg*rfs1Δpst3Δycp4Δyps1Δ*) cells, using anti-CgPst2 antibody. The intensity of individual bands in 4 independent Western blots was quantified using ImageJ densitometry software, and CgPst2 signal was normalized to the corresponding CgGapdh signal. Fold-change (mean ± SEM) in CgPst2 levels in treated cells, compared to untreated cells (considered as 1.0), is shown underneath the blot. *p* ≤ 0.05; paired two-tailed Student’s *t* test. The green asterisk indicates non-specific band. **D-E.** Immunoblot analysis showing CgYps1-CgPst2 **(D)** and CgYps1-CgYps1^*D91A*^-CgPst2 **(E)** interaction upon MD treatment (90 μM for 90 min) in *T-KO* (Cg*rfs1Δpst3Δycp4Δ*) and *T-KOyps1Δ* (Cg*rfs1Δpst3Δycp4Δyps1Δ*) cells, using anti-CgPst2 antibody. 6 mg precleared cell lysates were incubated with anti-CgYps1 antibody-conjugated beads for 12 h at 4°C. After bead washing, bead-bound proteins were boiled in 2X-SDS loading buffer and resolved on 4–20% polyacrylamide gradient and 12% polyacrylamide gels for CgYps1 and CgPst2 samples, respectively. For input samples, 60, 60 and 200 μg protein were loaded to detect CgPst2, CgGapdh and CgYps1 proteins, respectively. Two to three independent Western blots were quantified using the ImageJ densitometry software, and CgPst2 signal was normalized to the corresponding CgGapdh signal. Fold-change in CgPst2 levels in treated cells, compared to untreated cells (considered as 1.0), is shown underneath the blot. *p* ≤ 0.05; paired two-tailed Student’s *t* test. The red arrow marks CgPst2 band, while the green asterisk denotes non-specific band.

Since CgPst2 is pivotal to MD detoxification (Fig 1A), we next checked the effect of MD treatment on CgYps1-mediated processing of CgPst2. For this, we performed four experiments. First, we measured CgPst2 cleavage, and found about 1.9-fold increase in the levels of the C-terminal cleaved fragment of CgPst2 in MD-treated *wt* cells, compared to untreated cells (Fig 4A). Second, we determined the effect of MD-induced increase in cleavage of CgPst2 on CgPst2 activity. We found that the NADH:quinone oxidoreductase activity of CgPst2 was also higher upon MD treatment (Fig 4B). These data indicate that MD treatment enhances the C-terminal processing of CgPst2 and stimulates its activity. Intriguingly, *Cgyps1Δ* cells also responded to MD treatment by elevating NADH:quinone oxidoreductase activity (Fig 4B), indicating that CgPst2 is functional in this mutant, consistent with the mild MD sensitivity of the *Cgyps1Δ* mutant (Fig 2A). This result also suggests that CgYps1 is unlikely to be the sole mediator of MD-induced increase in CgPst2 activity, and CgPst2 functions.

Third, to check if MD treatment has any effect on *CgPST2* transcript and protein levels, we first generated antibody against *E*. *coli*-purified CgPst2 protein, and checked its specificity. This antibody recognized both CgPst2 and CgRfs1, however, no signal was seen in *Q-KO* cells lacking all four Fld-LPs (S9A Fig). Next, we generated *Cgrfs1Δpst3Δycp4Δ* (*T-KO* from hereon) and *Cgrfs1Δpst3Δycp4Δyps1Δ* (*T-KOyps1Δ* from hereon) strains to specifically amplify the *CgPST2* gene, as *CgPST2*-amplification-specific primers showed cross-reactivity, probably, owing to high sequence similarity among *CgFld-LP* genes. Measurement of endogenous *CgPST2* transcript and protein amounts revealed no change in transcript (S9B Fig) and about 1.7-fold higher protein levels (Fig 4C) of CgPst2 in MD-treated *T-KO* and *T-KOyps1Δ* cells, respectively, compared to corresponding untreated cells. Of note, we could not verify CgYps1-mediated CgPst2 cleavage with anti-CgPst2 antibody due to two reasons. First, anti-CgPst2 antibody did not recognize the 3 kDa C-terminal cleaved fragment of CgPst2. Second, the size difference between the cleaved N-terminal CgPst2 fragment and the full-length CgPst2, probably owing to other posttranslational modifications, was not conspicuous on SDS-PAGE analysis of cell extracts. However, whether CgPst2 undergoes any posttranslational modification is yet to be determined. In this context, it is noteworthy that Ycp4 in *S*. *cerevisiae* [36] and Pst2 in *C*. *albicans* [37] are known to undergo palmitoylation and ubiquitination, respectively. Alternatively, it is also possible that CgPst2 cleavage occurs at a low rate, and the cleaved N-terminal CgPst2 fragment represents a minor species of CgPst2 under *in vitro* conditions, thereby rendering detection very difficult. Altogether, these data suggest that MD treatment elevates CgPst2 protein levels without affecting its transcriptional regulation.

Fourth, we checked the effect of MD treatment on CgYps1-CgPst2 interaction. We observed that despite increased levels of CgPst2 (Fig 4C) and CgYps1 (S9C Fig) proteins upon MD treatment, the interaction between CgPst2-CgYps1 was less, as anti-CgYps1 antibody pulled down 40% lower amount of CgPst2 in MD-treated cells, compared to untreated cells (Fig 4D). Consistently, *Q-KO* cells expressing *CgPST2* ectopically showed weaker CgYps1-CgPst2 interaction upon MD treatment (S9D Fig). This diminished CgYps1-CgPst2 interaction in MD-treated cells could be due to enhanced cleavage of CgPst2 by CgYps1, and the consequent release of CgPst2 from CgYps1. We reasoned, if this is true, the interaction of catalytically-dead CgYps1 with CgPst2, should not change upon MD treatment, as the catalytically inactive CgYps1 will not be able to process CgPst2, thereby continuing to remain associated with CgPst2. To test this notion, we checked the interaction of CgYps1^*D91A*^ [one catalytic aspartate (D91) substituted with alanine] [15] with CgPst2 through co-immunoprecipitation analysis. We found that MD treatment had no significant effect on CgYps1^*D91A*^-CgPst2 interaction, as it was similar between untreated and MD-treated *C*. *glabrata* cells (Fig 4E), indicating that the release of CgPst2 from CgYps1, upon MD treatment, requires CgYps1’s proteolytic activity. Of note, to demonstrate that CgYps1^*D91A*^ is catalytically inactive, we purified CgYps1 and CgYps1^*D91A*^ from *Pichia pastoris* (S10A Fig), and checked their proteolytic activity through gelatin hydrolysis (S10B Fig) and hemoglobin digestion (S10C Fig) assays. We found CgYps1^*D91A*^ to be enzymatically inactive (S10B and S10C Fig).

Altogether, these results suggest that the loss of CgYps1-CgPst2 interaction upon MD treatment is likely to be due to enhanced CgYps1-mediated cleavage of CgPst2 and subsequent release of CgPst2. These data also reinforce that MD treatment enhances CgYps1-dependent cleavage of CgPst2, which may stimulate CgPst2 activity by removing the C-terminal region. Thus, we hypothesized that the C-terminally truncated CgPst2 will be more active than CgPst2.

### CgPst2-*C*Δ^*R174-F198*^ complements MD sensitivity of *CgPST2*-deleted strains

**Fig 5:**
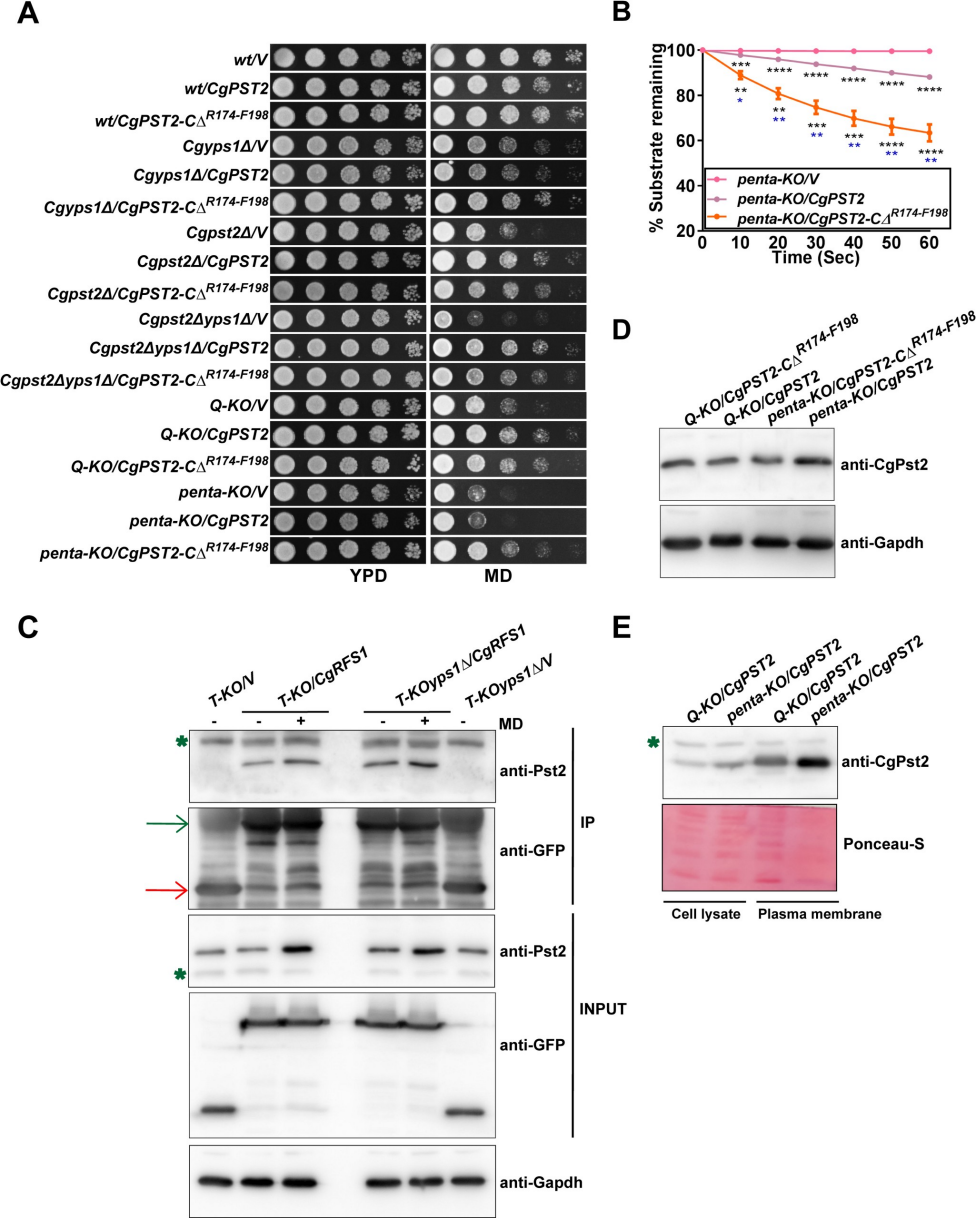
CgPst2-*C*Δ^*R174-F198*^ possesses higher activity than CgPst2 in *penta-KO*. **A.** Serial dilution spotting assay showing *CgPST2-C****Δ***^*R174-F198*^-mediated rescue of MD (80 μM) sensitivity. V, pRK74 vector. The *Q-KO* strain lacks four flavodoxin-like proteins (Fld-LPs), CgPst2, CgRfs1, CgPst3 and CgYcp4, while the *penta-KO* strain lacks CgYps1 along with four Fld-LPs, CgPst2, CgRfs1, CgPst3 and CgYcp4. **B.** NADH:quinone oxidoreductase activity in cell extracts of *penta-KO* expressing *CgPST2* or *CgPST2-C****Δ***^*R174-F198*^. Data represent mean ± SEM (n = 3). Black and blue asterisks represent statistically significant activity differences in indicated strains compared to *penta-KO/V* and *penta-KO/CgPST2*, respectively. *, p < 0.0332; **, p < 0.0021; ***, p < 0.0002; ****, p < 0.0001; Grouped multiple *t*-test. V, pRK74 vector. **C.** Immunoblot analysis showing interaction between CgPst2 and CgRfs1-GFP. 3 mg precleared lysates of untreated and MD (90 μM for 90 min)-treated *T-KO* (Cg*rfs1Δpst3Δycp4Δ*) and *T-KOyps1Δ* (Cg*rfs1Δpst3Δycp4Δyps1Δ*) cells expressing CgRfs1-GFP were incubated with anti-GFP antibody-conjugated beads, followed by Western analysis. The red and green arrows mark GFP and CgRfs1-GFP protein bands, respectively, in IP samples, while the green asterisk denotes non-specific band. **D.** Immunoblot analysis of CgPst2 levels in *Q-KO* and *penta-KO* strains expressing *CgPST2* or *CgPST2-C****Δ***^*R174-F198*^. **E.** Immunoblot analysis showing enrichment of CgPst2 in plasma membrane fractions of the *Q-KO* and *penta-KO* strains expressing *CgPST2*. Whole-cell lysates and plasma membrane fractions (60 μg), prepared by glass bead lysis and sucrose gradient ultracentrifugation, respectively, were resolved on 12% polyacrylamide gel, and probed with anti-CgPst2 antibody. The ponceau S-stained membrane is shown as loading control. The green asterisk denotes non-specific band.

CgPst2 is a 198 aa long protein. To test our hypothesis, we generated C-terminally truncated CgPst2 (CgPst2-*C*Δ^*R174-F198*^), that lacked last 25 amino acids from R174 onwards, and performed two experiments: Activity determination of CgPst2 and CgPst2-*C*Δ^*R174-F198*^ in cell lysates of mutants lacking or containing CgYps1, and Complementation analysis of MD sensitivity of different mutants. We found CgPst2-*C*Δ^*R174-F198*^ to rescue MD sensitivity of all *CgPST2*-deleted strains, *Cgpst2Δ*, *Cgpst2Δyps1Δ*, *Q-KO* and *Q-KOyps1Δ* (referred as *penta-KO* from hereon) (Fig 5A), suggesting that CgPst2-*C*Δ^*R174-F198*^ is functionally active. Surprisingly, we did not observe good rescue of MD sensitivity of *penta-KO* cells that were expressing *CgPST2* (Fig 5A). Further, consistent with our hypothesis and MD growth phenotypes, we found a much faster rate of NADH oxidation in cell extracts of *penta-KO* expressing *CgPST2*-*C*Δ^*R174-F198*^, compared to *CgPST2*-expressing *penta-KO* strain (Fig 5B), thereby suggesting that CgPst2-*C*Δ^*R174-F198*^ is more active than CgPst2.

Notably, CgPst2-mediated reversal of MD sensitivity of *Cgpst2Δyps1Δ* mutant but not of *penta KO* strain (Fig 5A) implicate other Fld-LPs (CgRfs1, CgPst3 and CgYcp4) in the regulation of CgPst2 activity. Therefore, to check if CgPst2 associates with other Fld-LPs, we tagged CgRfs1, CgPst3 and CgYcp4 proteins with the GFP epitope at their C-termini, and expressed in the *T-KO* strain which contains only CgPst2. Immunoprecipitation analysis revealed CgPst2 to interact with CgRfs1 under normal and MD treatment conditions in both *T-KO* and *T-KOyps1Δ* cells (Fig 5C). Notably, we could not detect CgPst2 and CgYcp4 interaction (S11 Fig), which could either be due to low CgYcp4 protein expression (S11 Fig), or CgPst2-CgYcp4 interaction may require the presence of CgRfs1. Despite multiple efforts, we could not check CgPst2-CgPst3 interaction, as we were unable to obtain good expression of CgPst3-GFP.

Further, the reduced functioning of CgPst2 in *penta-KO* cells raised three possibilities. First, CgPst2, compared to CgPst2-*C*Δ^*R174-F198*^, is not expressed well in *penta-KO* cells. Second, CgPst2 is mislocalized in *penta-KO*. Third, CgYps1-mediated processing of CgPst2/CgYps1-CgPst2 interaction is important for CgPst2 functions in MD stress survival. To determine which of these possibilities accounts for the reduced functions of CgPst2 in the *penta-KO* strain, we first checked CgPst2 expression in *Q-KO* and *penta-KO* cells expressing *CgPST2* or *CgPST2-CΔ*^*R174-F198*^. We observed good expression of CgPst2 and CgPst2*-*CΔ^*R174-F198*^ in both *Q-KO* and *penta-KO* (Fig 5D). In fact, the amount of CgPst2 was higher in *penta-KO* cells, compared to *Q-KO* cells (Fig 5D), indicating that MD sensitivity of the *penta-KO*/*CgPST2* strain is not due to diminished CgPst2 protein levels.

Next, we checked the localization of CgPst2 by subcellular fractionation analysis in *CgPST2*-expressing *Q-KO* and *penta-KO* strains. For this, we collected plasma membrane (PM) fractions via sucrose density gradient ultracentrifugation of cell extracts of *Q-KO* and *penta-KO* strains expressing *CgPST2*. The purity of the obtained PM fraction was evaluated by immunoblot analysis using anti-CgPma1 antibody (S12 Fig), which detects the plasma membrane proton pump CgPma1 [13]. Notably, the signal for cytosolic CgGapdh was also largely absent in the PM fraction (S12 Fig). Anti-CgPst2 Western analysis revealed CgPst2 to be enriched in the PM fraction of both strains, with the *penta-KO/CgPST2* strain also showing higher amounts of CgPst2 in cell lysates and PM samples, compared to that of the *Q-KO/CgPST2* strain (Fig 5E). These data indicate that the localization of CgPst2 to the plasma membrane is not dependent upon CgYps1, and the reduced functions of CgPst2 in *penta-KO/CgPST2* strain is unlikely to be owing to CgPst2 mislocalization.

Further, the enrichment of CgPst2 in the PM fraction prompted us to examine the presence of CgYps1 in this fraction, with the rationale that CgPst2 and CgYps1 interaction may occur at the plasma membrane. Notably, we detected a good signal with anti-CgYps1 antibody in the PM sample of the *Q-KO/CgPST2* strain, which was absent in the *penta-KO* strain (S12 Fig), thereby localizing CgYps1 to the plasma membrane. Given that both CgYps1 and CgPst2 were enriched in the PM fraction (S12 Fig), the CgYps1-mediated processing of CgPst2 is likely to take place at the plasma membrane. Altogether, these data also implicate CgYps1 in regulation of CgPst2 protein levels. Moreover, the higher activity of CgPst2*-*CΔ^*R174-F198*^ than CgPst2 in the *penta-KO* strain (Fig 5B) suggests that CgYps1-dependent cleavage may relieve the inhibitory effect of the C-terminal region on CgPst2 activity.

Overall, we draw six major conclusions from these data. First, CgPst2 interacts with at least one other Fld-LP, CgRfs1. Second, CgRfs1, CgPst3 and CgYcp4 are important for CgPst2 functions in MD resistance, as MD treatment could stimulate CgPst2 activity in the *Cgyps1Δ* mutant, despite the lack of CgPst2 activation owing to CgYps1-mediated processing. This increase in CgPst2 activity in *Cgyps1Δ* mutant may arise from association of CgPst2 with other flavodoxin-like proteins. Third, CgYps1-mediated processing appears to be essential for CgPst2 functions in MD detoxification only in the absence of other Fld-LPs, underscoring that CgYps1-mediated cleavage of CgPst2 may reflect one of many mechanisms controlling CgPst2 activity. Fourth, both CgYps1 and CgPst2 localize to the plasma membrane, and the plasma membrane localization of CgPst2 is not dependent on CgYps1. Fifth, despite the presence of a functional CgPst2, MD sensitivity of the *Cgyps1Δ* mutant points to a dual role for CgYps1 in MD resistance: CgPst2-dependent, and a yet to be identified CgPst2-independent function. Finally, since the C-terminal truncation resulted in an active CgPst2 enzyme, CgYps1-mediated cleavage of CgPst2 is likely to be a regulatory event that modulates CgPst2 activity in response to internal and external cues.

### CgPst2^*R174A*^ is proficient in MD detoxification

**Fig 6:**
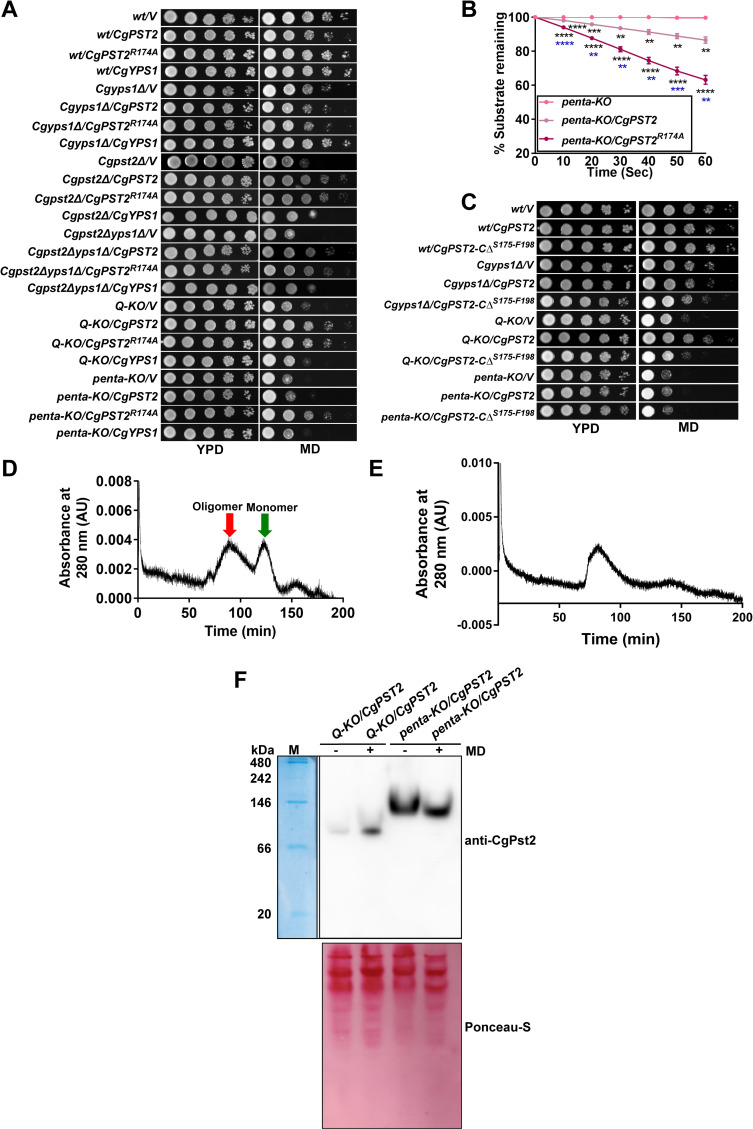
CgPst2^*R174A*^ rescues MD sensitivity of *CgPST2*-deleted strains. **A.** Serial dilution spotting assay showing *CgPST2*^*R174A*^-mediated rescue of MD (80 μM) sensitivity. V, pRK74 vector. The *Q-KO* strain lacks four flavodoxin-like proteins (Fld-LPs), CgPst2, CgRfs1, CgPst3 and CgYcp4, while the *penta-KO* strain lacks CgYps1 along with four Fld-LPs, CgPst2, CgRfs1, CgPst3 and CgYcp4. **B.** NADH:quinone oxidoreductase activity in cell extracts of *penta-KO* expressing *CgPST2* or *CgPST2*^*R174A*^. Data represent mean ± SEM (n = 3). Black and blue asterisks represent statistically significant activity differences in indicated strains compared to *penta-KO* and *penta-KO/CgPST2*, respectively. **, p < 0.0021; ***, p < 0.0002; ****, p < 0.0001; Grouped multiple *t*-test. **C.** Serial dilution spotting assay showing MD (80 μM) sensitivity of *Q-KO* and *penta-KO* strains expressing *CgPST2-CΔ*^*S175-F198*^. **D-E.** Size exclusion chromatograms of purified CgPst2^*R174A*^ **(D)** and CgPst2-CΔ^*R174-F198*^ **(E)**. After loading on the Sephacryl S-200 column, protein elution profiles were determined using the absorbance values at 280 nm. The CgPst2^*R174A*^ showed two peaks corresponding to 296 kDa (red arrow; oligomeric form) and 29 kDa (green arrow; monomeric form) sizes, and CgPst2-CΔ^*R174-F198*^ showed one peak of 296 kDa. **F.** Native PAGE analysis showing increased oligomer formation upon MD treatment. 200 μg whole cell lysates of untreated and MD (90 μM for 90 min)-treated *Q-KO* and *penta-KO* expressing *CgPST2* were resolved in a discontinuous Tris-glycine buffer system under non-denaturing conditions, and probed with anti-CgPst2 antibody. The native protein molecular weight marker (M) was stained with coomassie brilliant blue, and is shown on the right side of the blot. The ponceau S-stained membrane is shown as loading control.

Arg-174 is essential for CgPst2 cleavage (Fig 3D). Therefore, to determine the functional role of R174 residue in regulating CgPst2 activity, we expressed CgPst2 carrying alanine substitution of the R174 residue (CgPst2^*R174A*^), and found it to complement MD sensitivity of *Cgpst2Δ*, *Cgpst2Δyps1Δ*, *Q-KO* and *penta-KO* strains (Fig 6A). Similar to CgPst2-CΔ^*R174-F198*^, CgPst2^*R174A*^ also possessed higher NADH:quinone oxidoreductase activity than CgPst2 in *penta KO* (Fig 6B). These results were unexpected, as CgPst2^*R174A*^ is not cleaved by CgYps1, and thus, should retain the C-terminal region. Therefore, to check whether CgPst2^*R174A*^ is hyperactive or expressed at a higher level, we performed Western analysis and assessed the protein amounts in extracts of the *Q-KO* and *penta-KO* cells expressing *CgPST2* or *CgPST2*^*R174A*^. We found similar amounts of CgPst2^*R174A*^ in both *Q-KO* and *penta-KO* strains (S13A Fig). Notably, CgPst2, which was unable to rescue MD sensitivity of the *penta-KO* strain (Fig 5A), showed 6-fold higher abundance than CgPst2^*R174A*^ (S13A Fig), indicating that the better functioning of CgPst2^*R174A*^ in *penta-KO* cells is not due to high protein levels.

Further, to probe deeper into the active nature of CgPst2^*R174A*^ in the *penta-KO* strain, we generated and expressed CgPst2-*C*Δ^*S175-F198*^ protein that lacked last 24 amino acids after R174. CgPst2-*C*Δ^*S175-F198*^ and CgPst2-*C*Δ^*R174-F198*^ differ from each other, only in one residue, with CgPst2-*C*Δ^*S175-F198*^ containing R174. As expected, compared to the full-length CgPst2, both these C-terminally truncated proteins ran faster on SDS-PAGE, corresponding to a molecular weight of about 19 kDa (S13B Fig). However, unlike CgPst2-*C*Δ^*R174-F198*^, CgPst2-*C*Δ^*S175-F198*^ expression neither reversed MD sensitivity of *Q-KO* and *penta KO* cells (Fig 6C) nor exhibited NADH:quinone oxidoreductase activity (S14A Fig), despite good protein expression (S14B Fig). Altogether, these data suggest that CgPst2-*C*Δ^*S175-F198*^ is non-functional, and underscore the importance of R174 removal in activation of CgPst2, with CgPst2-*C*Δ^*S175-F198*^, CgPst2-*C*Δ^*R174-F198*^ and CgPst2^*R174A*^ possessing no, higher and higher activity, respectively, compared to the full-length CgPst2.

### CgPst2 processing regulates structural state

Fld-LPs are known to exist in tetramer forms, with oligomeric forms exhibiting elevated stability and/or activity [27,30,38]. The recombinant full-length CgPst2 existed in a monomeric form (Fig 1E), and was inactive (S3B Fig). Owing to differential activities of the C-terminally truncated CgPst2 proteins, we decided to examine the assembly state of these proteins. For this, we expressed and purified 6XHis-FLAG-CgPst2^*R174A*^, CgPst2-*C*Δ^*R174-F198*^ and CgPst2-*C*Δ^*S175-F198*^ from *E*. *coli* (S15A–S15C Fig). Despite being functional in *C*. *glabrata*, CgPst2^*R174A*^ and CgPst2-*C*Δ^*R174-F198*^ proteins showed no NADH:quinone oxidoreductase activity (S15D and S15E Fig), suggesting that these recombinant proteins probably lost enzymatic activity during purification, are misfolded, or require post-translational modification/s for activity. SEC analysis revealed two forms of CgPst2^*R174A*^, a monomer of 29 kDa and an oligomer of 296 kDa (Fig 6D), indicating that R174 residue may impede CgPst2 oligomerization. Notably, CgPst2-*C*Δ^*R174-F198*^ was found only in the oligomeric form of 296 kDa (Fig 6E), while CgPst2-*C*Δ^*S175-F198*^ formed aggregates and could not enter the column (S15F Fig). These data suggest that the role of C-terminal region in proper folding and function of CgPst2 is governed by R174 residue. Overall, the SEC analysis of different CgPst2 variants raises the prospect that CgPst2 exists as a monomer, and R174 removal, and by extension, the proteolytic cleavage of CgPst2 by CgYps1, may stimulate CgPst2 oligomerization. However, since all CgPst2 (wild-type, C-terminally truncated and R174-mutated) proteins, purified from *E*. *coli*, were enzymatically inactive, there is a good possibility that the misfolded CgPst2 contributes to their varied structural states.

We, therefore, next asked the question whether CgPst2 exists in a homo-oligomeric form in *C*. *glabrata* and if so, whether this oligomeric state is altered by the presence of menadione and/or CgYps1. For this, we subjected whole cell extracts of *Q-KO* and *penta-KO* strains expressing *CgPST2* to native PAGE analysis, followed by Western blotting with anti-CgPst2 antibody. We observed about a 90 kDa band, probably representing CgPst2 tetramer, in *CgPST2*-expressing *Q-KO* cells, whose intensity was significantly increased upon MD treatment (Fig 6F). Intriguingly, this CgPst2 oligomer species was neither present in untreated nor MD-treated *CgPST2*-expressing *penta-KO* cells (Fig 6F). Instead, a higher-order CgPst2 band of about 130 kDa was observed in untreated and MD-treated *CgPST2*-expressing *penta-KO* cells (Fig 6F), which may represent the non-functional aggregated form of CgPst2. Further, the *penta-KO* cells expressing functionally active, C-terminally truncated CgPst2-CΔ^*R174-F198*^ and arginine-mutated CgPst2^*R174A*^ protein displayed the 90 kDa CgPst2 band, suggesting that these proteins are able to form a tetramer in *C*. *glabrata* cells, which may contribute to CgPst2 activity (S16 Fig). Altogether, three key findings emerge from these data. First, CgPst2 exists in a homo-oligomeric form. Second, MD treatment enhances CgPst2 homo-oligomerization. Third, CgYps1 is required for homo-oligomerization of CgPst2. Of note, although, we could neither detect monomeric nor higher-order oligomeric (296 kDa) form of CgPst2 in native PAGE immunoblot analysis, as observed in SEC analysis, it is possible that these CgPst2 species either represent a minor form of CgPst2 in *C*. *glabrata* or misfolding of CgPst2 in *E*. *coli* accounts for its inability to form tetramers. Further studies are warranted to address these possibilities as well as to examine higher-order homo-oligomers, formed by recombinant CgPst2-*C*Δ^*R174-F198*^ and CgPst2-*C*Δ^*R174A*^ proteins, and CgPst2 in *penta-KO/CgPST2* strain. Additionally, the possibility of post-translational modifications and/or binding of CgPst2 with other proteins contributing to the higher molecular weight CgPst2 bands in *C*. *glabrata* is yet to be precluded.

## Discussion

Yapsins represent a family of GPI-anchored aspartyl proteases that is unique to fungi [35]. These proteases consist of two subunits α and β, with each subunit providing a catalytic aspartate residue to the active site cleft, predominantly cleave C-terminally to monobasic or dibasic residues, and are pivotal to fungal physiology and virulence [12,35,39]. Despite invasive fungal pathogens killing about one and a half million people every year [1], our understanding of the stress survival mechanisms of human pathogenic fungi remains woefully inadequate. CgYps1, an aspartyl protease, occupies a central stage in the regulation of many physiological processes, that are modulated by the CgYapsin family in the pathogenic yeast C. *glabrata*. Here, we have identified the potential interactors of a fungal Yapsin, through a pull-down assay, and characterized, in detail, the flavodoxin-like protein CgPst2, of 19 CgYps1-interacting proteins identified. We show that CgPst2 possesses NADH:quinone oxidoreductase activity, and is required for MD and BQ resistance. We report CgPst2 as the first physiological target of the CgYps1 protease, as it undergoes processing in a CgYps1-dependent manner in *C*. *glabrata*. This inference was further strengthened by an *in vitro* cleavage assay wherein CgYps1, purified from *P*. *pastoris* culture supernatants, could cleave the recombinant CgPst2 protein (S17 Fig), thereby underscoring the significance of CgYps1-CgPst2 interaction identified in the current study.

Flavodoxin-like fold-containing proteins, which adopt the conserved three-dimensional α/β twisted open-sheet fold, possess two-electron NAD(P)H:quinone oxidoreductase activity, and play a major role in stress response in diverse organisms including bacteria, yeasts, plants and mammals, by inhibiting redox cycling [17,18,22,25,27,28,30]. These have also been implicated in metabolic gene regulation, stress tolerance and virulence [23,25,30]. Several distinct mechanisms are used to control the activity of Fld-LPs which differ, in their oligomeric state, amino acid sequence, flavin cofactor and redox partners, from one another [17–20]. Fld-LPs use ping-pong mechanism, with FMN or FAD as cofactor, and NADH and quinone as electron donor and acceptor, respectively, to detoxify quinones via two-electron reduction pathway [17–19]. We report for the first time that CgYps1-mediated cleavage is a key determinant of activity and oligomeric state of cellular CgPst2, that adds another wholly unexpected regulatory mechanism to the Fld-LP stress defense system.

Structural studies on *E*. *coli wrbA* and *S*. *cerevisiae* Pst2 show that they form tetramer, irrespective of the FMN cofactor binding [27,30,40]. Multimerization brings many regions together and creates a complex active site [29]. An extended C-terminus has been postulated to inhibit the oligomerization of Fld-LPs, with *S*. *cerevisiae* Pst2 forming a tetramer, while Ycp4, containing additional 49 amino acids at the C-terminus, probably existing in a dimeric form, with both proteins implicated in quinone tolerance [22–24,30]. Similarly, rat and human NQO1 [NAD(P)H:quinone oxidoreductase 1)] are dimeric enzymes [41], while *E*. *coli wrbA*, which lacks the C-terminal sub-region of NQO1, forms a tetramer [27]. Furthermore, serine substitution of the proline-187 residue, a common cancer-associated polymorphism, in the C-terminal region of NQO1 resulted in NQO1 dimer destabilization and loss of catalytic activity [42,43]. The flavodoxin-like C-terminal domain of *E*. *coli* catalase HPII has also been implicated in its stability [44]. Consistent with all this, we found R174 residue in the C-terminus of CgPst2 to be essential for CgYps1-dependent cleavage, and consequent modulation of CgPst2 oligomerization and activity. The reduced CgPst2 activity and the lack of CgPst2 tetrameric form in *penta KO*, and contrasting effects of two C-terminal-truncations, lacking or containing R174, on CgPst2 activity, highlight the functional relevance of CgYps1-mediated CgPst2 processing in MD stress survival. However, given the conserved nature of R174 across several Fld-LPs (S7 Fig), and with this single residue mutation being sufficient to alter the activity of CgPst2, it will be intriguing to investigate if this residue is a protease target in other organisms as well.

Despite increased expression upon MD exposure (Fig 4E), localization of *C*. *glabrata* Pst2 at the plasma membrane remains unchanged under regular and MD stress conditions (S5 Fig). Notably, while the high throughput studies revealed *S*. *cerevisiae* Pst2, which is induced upon oxidative stress, to be localized to the mitochondria [45,46], the GFP fusion proteins of Pst2, Rfs1 and Ycp4 in *S*. *cerevisiae* have been reported to be localized to the eisosome domain in the plasma membrane [47]. Through confocal imaging and biochemical analysis, we showed that CgPst2 is present at the plasma membrane in *C*. *glabrata*. Similarly, *C*. *albicans* Pst2 is also located at the plasma membrane [25]. Altogether, these findings raise the possibility of cell membrane-localized Fld-LPs conferring growth advantage to fungal cells under oxidative stress-generating environmental conditions.

Regulated proteolysis is a common mechanism to modulate protein structure, function, localization and turnover [48]. Here, we demonstrate the essentiality of R174 residue in CgPst2 for CgYps1-dependent cleavage, which releases the C-terminal region. Conceivably, like other proteolysis events, multiple cellular factors including growth-phase, ROS abundance, expression of other Fld-LPs, environmental pH and CgYps1 availability, may impact the processing of CgPst2. In our analysis, the short cleaved C-terminal fragment was detected in small and variable amounts which could be due to its unstable nature. Alternatively, a small amount and non-detection of the cleaved C-terminal and N-terminal fragment of CgPst2, respectively, may indicate that only a minor fraction of CgPst2 undergoes cleavage under laboratory growth conditions.

Of note, of four Fld-LPs, the sole requirement for CgPst2 for MD and BQ resistance places CgPst2 at the forefront of hydrogen peroxide and superoxide anion radical detoxification mechanisms. Therefore, the existence of an exquisite control for CgPst2 activity is not unexpected in the pathogenic yeast *C*. *glabrata*, which encounters, and successfully counteracts the host-macrophage-elicited oxidative stress, and proliferates in macrophages [12,49,50]. In this context, it is noteworthy that the phagolysosomes of activated neutrophils have been shown to contain elevated amounts of ubiquinone [51]. So besides detoxifying endogenous ubiquinone of the electron transport chain, CgFld-LPs may aid *C*. *glabrata* survive attack of the host immune system.

Intriguingly, our genetic data suggest that MD sensitivity of the *Cgyps1Δ* mutant is independent of CgPst2, as the active CgPst2 constructs, CgPst2-*C*Δ^*R174-F198*^ and CgPst2^*R174A*^, could not restore growth of the *Cgyps1Δ* mutant on MD medium, and the double *Cgpst2Δyps1Δ* mutant exhibited MD sensitivity higher than single mutants. Moreover, the MD-induced increase in CgPst2 activity in *Cgyps1Δ* mutant indicates that CgYps1 is unlikely to be the sole regulator of CgPst2 activity. This notion was further corroborated with CgYps1-mediated processing being important for CgPst2 functions in MD detoxification only in the absence of other Fld-LPs.

**Fig 7:**
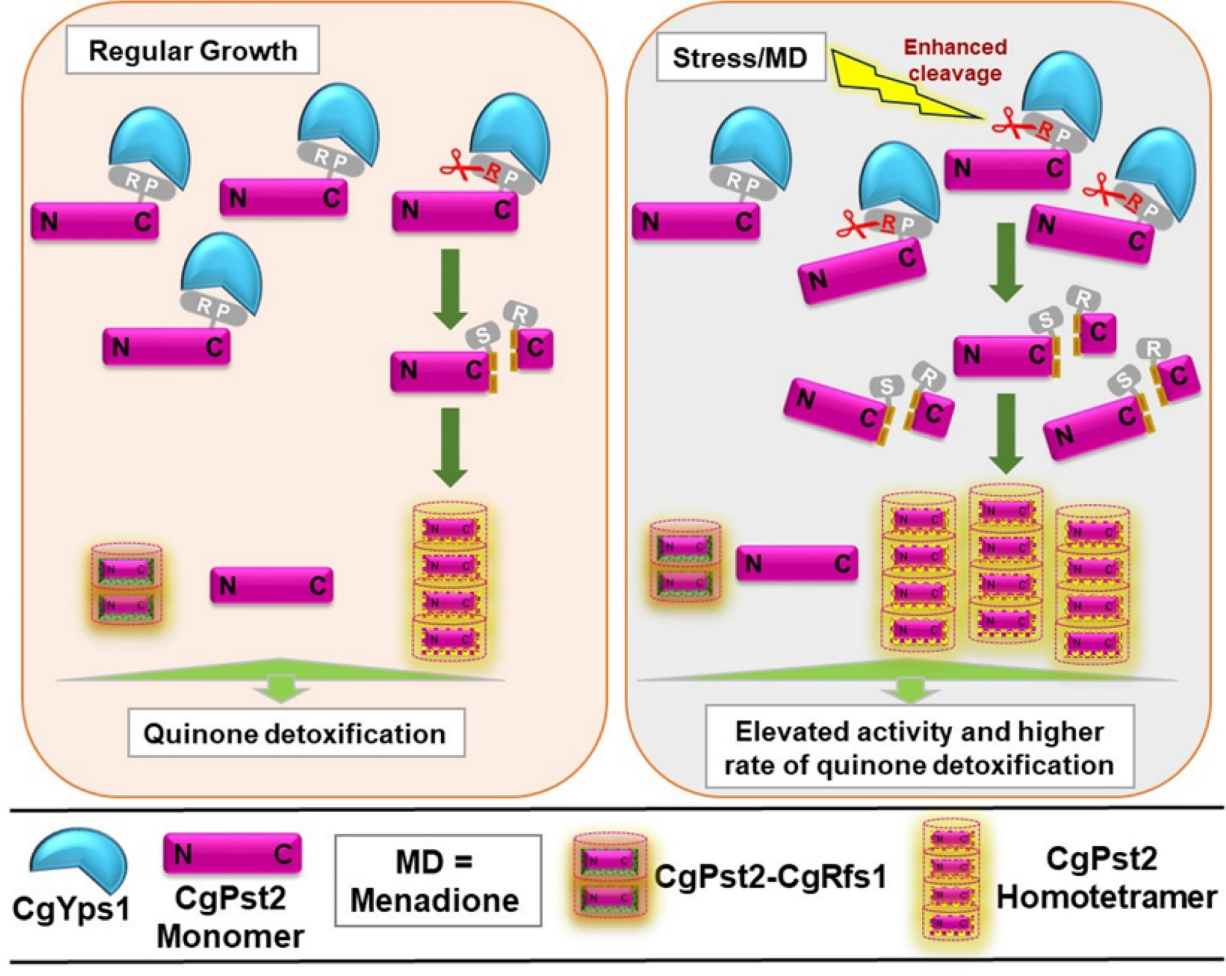
A schematic summarizing key findings of the study. Arginine-174 (R) and Proline-176 (P) residues of CgPst2 are predicted to interact with CgYps1 at the plasma membrane, and CgYps1 processes R174 residue in the C-terminus of CgPst2. This cleavage, which is elevated upon menadione treatment, leads to removal of the C-terminal domain, resulting in CgPst2 homo-tetramerization, higher activity and efficient quinone detoxification. Of note, the signal, that stimulates CgYps1-mediated cleavage of CgPst2, is not known. CgPst2 also interacts with CgRfs1, however, CgPst2-CgRfs1 association is not dependent on CgYps1, and occurs under both regular and MD treatment conditions. Altogether, CgPst2 functions are regulated at multiple levels, and CgYps1-mediated cleavage of CgPst2 may reflect one of many mechanisms controlling CgPst2 activity.

Cleavage and oligomerization, resulting in CgPst2 activation, are likely to be linked events that may occur at the plasma membrane and constitute cellular response to the quinone stress. Of note, the tetrameric form of *E*. *coli wrbA* is shown to be more thermostable than monomeric or dimeric forms [38]. Three particularly intriguing findings of our study, the homo-tetrameric form of CgPst2 in *Q-KO*/*CgPST2* cells, CgPst2-mediated rescue of MD sensitivity of *Q-KO* and *Cgpst2Δyps1Δ* mutant, but not of *penta-KO* (summarized in S2 Table), that lacks CgPst2 tetramer, and the interaction of CgPst2 with CgRfs1, suggest that CgPst2 is capable of forming both homo-oligomer, and heteromers, with CgYps1 and CgFld-LP availability and/or cellular context probably dictating the type of oligomers formed. Therefore, we propose a complex multilayered regulation for NADH:quinone oxidoreductase activity of CgPst2, that could be governed by CgPst2 assembly. Given the absence of CgPst2 tetramer, and reduced CgPst2 activity in *penta-KO/CgPST2* cells, we posit that CgYps1-mediated processing releases the C-terminal domain and induces CgPst2 homo-tetramerization, with the homo-tetrameric state being more active (Fig 7). In light of this, it is tempting to speculate that CgYps1-CgPst2 interaction aids in keeping a readily available cellular pool of CgPst2 for activation, when the need arises. Thus, the R174 residue, and, by extension, CgYps1-mediated cleavage, is likely to be pivotal to maintain a balance between differentially active forms of CgPst2, based on the cellular requirement. However, it must be noted that an experimental demonstration of menadione-induced, CgYps1-dependent increase in the ratio of oligomeric to monomeric form of CgPst2 is required to prove the proposed model unequivocally.

Further, whether CgPst2 heteromers contain CgPst3 or CgYcp4, is yet to be determined. Additionally, the lack of *in vitro* phenotype for mutants deleted for *CgRFS1*, *CgPST3* and *CgYCP4* genes does not exclude their possible participation in oxidative stress-counteracting mechanisms, particularly in light of *Q-KO* showing attenuated survival in the mice infection model. How other Fld-LPs function, and if CgYps1 or other ten CgYapsins regulate their structural states and activity, remain to be investigated. In this context, it is worth noting that this is the first report of an aspartyl protease interacting with, and, regulating the activity of a Fld-LP through processing.

CgYps1 has a GPI-anchor region at the C-terminus, and is predicted to be present at the plasma membrane or the cell wall in *C*. *glabrata* [52] (www.candidagenome.org). Given the cell membrane localization of CgPst2 and CgYps1 (S12 Fig), CgYps1 and CgPst2 are likely to interact at the plasma membrane. However, since these are probably present on opposite sides of the plasma membrane, further studies are warranted to characterize, in-depth, the CgYps1-CgPst2 interaction at the plasma membrane. Similarly, the interaction of CgYps1 with the plasma membrane proton pump CgPma1, which has been identified as CgYps1 interactor in our IP-MS analysis (Table 1), is likely to take place at the plasma membrane, with CgYps1 being previously implicated in activity regulation of CgPma1 [13]. Moreover, Yps1 in *S*. *cerevisiae* is located at the plasma membrane [53], and CgYps1 ectopic expression rescued cell wall-related phenotypes of the *S*. *cerevisiae yps1Δ* mutant [54], indicating some molecular targets to be common to Yps1 protease of these two yeasts.

Substrate identification is necessary to elucidate the real functions of CgYapsins. Our IP-MS analysis identified, through a pull-down assay, 19 CgYps1-interacting proteins which are involved in both oxidoreduction process and energy homeostasis, and translation processes (Table 1). DeepLoc-based subcellular localization analysis revealed potential CgYps1 interactors to have varied unanticipated location ranging from the nucleus to the peroxisome (Table 1), indicating that the CgYps1-target protein repertoire is large, and not confined to the cell wall or the cell membrane proteins. However, the possibility that CgYps1 interacts non-enzymatically with some identified proteins and/or few highly abundant proteins are non-specific interactors, is yet to be ruled out. In this context, it is noteworthy that the cell wall targets of CgYps1 are likely to be underrepresented in this analysis of whole cell lysate samples. Moreover, since we could detect CgYps1 in the plasma membrane fraction (S12 Fig), it is possible that some of these identified interactions, including CgPst2-CgYps1, may occur at the plasma membrane. Additionally, the possibility of identified cytosolic proteins displaying cue-dependent dual subcellular localization is currently being investigated.

In conclusion, in addition to identifying intracellular interactors of a fungal yapsin for the first time, we report a novel regulatory mechanism for NADH:quinone oxidoreductase activity of a flavodoxin-like protein in *C*. *glabrata*, whose homologs are present in bacteria, plants and humans.

## Materials and methods

### Ethics statement

Mice infection experiments were performed at the Animal House Facility of Centre for DNA Fingerprinting and Diagnostics (CDFD), Hyderabad, India in accordance with guidelines of the Committee for the Purpose of Control and Supervision of Experiments on Animals, Government of India. Procedures were designed to minimize animal suffering, and approved by the Institutional Animal Ethics Committee [EAF/RK/CDFD/22]. Mice infection studies were performed, as described previously [50].

### Strains and media

All *C*. *glabrata* strains are derivatives of the vaginal isolate BG2, and routinely grown at 30°C on standard YPD medium. Logarithmic (log)-phase cells were obtained by growing overnight cultures for 4–5 h at 30°C in the fresh medium.

### Gene cloning and disruption

*C*. *glabrata* mutants were generated by replacing the ORF with the *nat1* gene, which confers nourseothricin resistance, using the homologous recombination-based approach, and gene replacements were confirmed by PCR, as described previously [55]. Multiple gene deletions were carried out in the same strain using the recyclable *nat1* selection marker, as described previously [56]. For mutant complementation and overexpression studies, *C*. *glabrata* genes were expressed from *PGK1* (pRK74 plasmid) and *PDC1* (pRK999 plasmid) promoter, respectively. For specific amplification of *CgPST2*, either the plasmid (pRK) containing *CgPST2* gene along with 500 bp of 5’and 3’ UTR or genomic DNA of the *Cgrfs1Δ* mutant, was used as template. The *CgPST2*^*R174A*^ allele was generated through two-fragment PCR approach, consisting of two separate PCR amplifications using mutagenesis primers, followed by fusion PCR and restriction digestion. The *CgPST2*, *CgPST2-CΔ*^*R174-F198*^, *CgPST2*^*R174A*^ and *CgPST2-CΔ*^*S175-F198*^ genes were cloned in XmaI-Xba1, Xba1-Spe1, XmaI-Xba1, and Xba1-Xho1 sites, respectively, in the pGRB2.1 plasmid for analysis in *C*. *glabrata*, while genes were cloned in EcoR1-Xho1 sites in pET28a(+) plasmid for studies in *E*.*coli*. For CgPst2 tagging with the triple epiotpe SFB (S protein-Flag-Streptavidin-binding peptide) and GFP at the C-terminus, *CgPST2 ORF* (*CAGL0K11858g*; 0.597 kb) was cloned in XbaI-SpeI sites in pRK1349 and pRK1000 plasmid, respectively, carrying the *CgPDC1* promoter. *CgYPS1*^*D91A*^ was generated using mutagenic primers, as described previously [15]. All plasmid clones were confirmed by sequencing, functional complementation or protein expression. Strains, plasmids and primers used in this study are listed in S3–S5 Tables, respectively.

### NADH:quinone oxidoreductase activity measurement

The NADH:quinone oxidoreductase activity in *C*. *glabrata* cell extracts was measured, as described previously for *E*. *coli*. purified proteins [57]. Briefly, log-phase cultures of *C*. *glabrata* strains were either treated with 90 μM MD for 90 min, or left untreated, and cell lysates were prepared by glass bead lysis method. Cell lysates (500 μg) were added to the reaction buffer [10 mM Tris-HCl (pH 7.4), 150 mM NaCl] containing NADH (500 μM) and menadione (500 μM). The blank control contained no NADH. The reaction was started with NADH addition, and the absorbance was recorded at 340 nm in a 1-cm-path-length quartz cuvette over a period of 60 seconds at 10 seconds interval on the Spectramax M5 plate reader. For CgPst2 and its variants purified from *E*. *coli*, the purified protein (50 μg) was incubated for 1 h with FMN (100 μM) or FAD (100 μM) on ice, followed by addition of other reaction components, as described above. The blank control contained no protein. The commercially available NAD(P)H:FMN oxidoreductase (1 Unit, Roche, # 10476480001) was used as positive control. Absorbance of the substrate NADH was considered as 100 at 0 h time point, and NADH oxidation was calculated by dividing the absorbance at each time point by 0 h absorbance, and multiplying the number by 100.

### Immunoprecipitation, mass spectrometry and Western blot analysis

For IP, log-phase *C*. *glabrata* cell extracts were prepared by glass bead lysis. For IP-MS analysis, using anti-CgYps1 antibody, rProtein A-Sepharose Fast Flow beads (GE Healthcare) were first incubated with cell extracts (2 mg) of *wt* and *Cgyps1-11Δ* strains in lysis buffer (15 mM Na_2_HPO_4_, 150 mM NaCl, 2% Triton X- 100, 0.1% SDS, 0.5% DOC, 10 mM EDTA, 0.02% NaN_3_) containing protease inhibitors for 4 h at 4°C, to remove proteins binding non-specifically to beads. These precleared lysate supernatants were incubated with antibody (20 μl)-coated rProtein A-Sepharose beads for 2–3 h at 4°C with gentle rocking on an end-to-end rotator. Beads were washed once with lysis buffer and thrice with wash buffer (50 mM NaCl, 10 mM Tris, 0.02% NaN_3_), and centrifuged at 1500 rpm for 2 min. After boiling in 2X SDS dye for 5 min, samples containing beads were run on 10% SDS-PAGE gel, until the dye front entered the resolving gel. After coomassie brilliant blue staining, gel sections containing protein bands were cut and sent for protein identification to the Taplin Biological Mass Spectrometry Facility, Harvard Medical School, Boston. The Sequest software was used to identify peptides, and peptides, filtered to 1% false discovery rate, were mapped to the *C*. *glabrata* reference proteome database (www.candidagenome.org/). Proteins identified with ≥ 2 unique peptides in both biological replicate samples of *wt* and *Cgyps1-11Δ* strains were selected for further analysis. For identification of CgYps1-specific interactors, proteins identified in the immunoprecipitated samples of *Cgyps1-11Δ* mutant were removed from the CgYps1 interactor list. The raw mass spectrometry proteomics data have been deposited to the ProteomeXchange Consortium via the PRIDE [58] partner repository with the dataset identifier PXD022766.

For affinity purification, CgPst2-SFB expressing log-phase cells were grown in CAA medium for 4–5 h and collected. Cell lysates (3 mg), were incubated with streptavidin beads for 2 h at 4°C. After washing, beads were boiled in 2X SDS dye, proteins resolved on a 12% and 8% SDS-PAGE gel for CgPst2 and CgYps1 detection, respectively, and immunoblotted with anti-Flag and anti-CgYps1 antibodies. For CgPst2 protein detection, log-phase cells were either left untreated or treated with MD (90 μM) for 90 min, and cell lysates were prepared in protein extraction buffer [50 mM Tris-HCl (pH 7.5), 2 mM EDTA, 2% glucose, 10 mM sodium fluoride, 1 mM sodium orthovanadate and 1 X protease inhibitor] using glass beads. Total protein (200 μg for CgYps1 and 60 μg for CgPst2) were resolved on SDS-PAGE gel, followed by transfer to the polyvinylidene difluoride (PVDF) membrane and probing with anti-CgPst2 or anti-CgYps1 antibody.

For native PAGE analysis, cell lysates (200 μg) were separated on a discontinuous Tris-glycine polyacrylamide gel system consisting of a 4.0% stacking and a 10% resolving gel, prepared in 0.375 M Tris-HCl, (pH 8.8), under non-denaturing conditions. The running buffer contained 25 mM Tris (pH 8.0) and 192 mM glycine. The Gel was pre-run for 30 min, prior to sample loading in 2X Native PAGE dye [Tris-HCl (62.5 mM; pH 6.8), glycerol (25%) and bromophenol blue (1%)]. Gels were run for 3–4 h at 100 V at 4^ο^C to avoid excessive heating, and proteins were transferred to PVDF membrane in the transfer buffer (25 mM Tris, 192 mM glycine, pH 8.0) containing 10% methanol for 14 h at 4^ο^C. The membrane was processed for immunoblot analysis using anti-CgPst2 antibody. The gel lane containing the protein molecular weight marker was cut, and stained with coomassie brilliant blue.

### In silico protein analysis

For molecular docking studies, CgPst2 and CgYps1 sequences were retrieved from CGD (Candida genome database; http://www.candidagenome.org), and submitted to the online tool I-TASSER (Iterative Threading ASSEmbly Refinement; https://zhanglab.ccmb.med.umich.edu/I-TASSER/). The top structure models with best Confidence (C)-score were selected and analyzed by another online tool Z-DOCK (http://zdock.umassmed.edu/) to acquire CgYps1-CgPst2 docking image.

### C-terminal-cleaved CgPst2 fragment analysis

Affinity purification using streptavidin beads was used to retrieve the small C-terminal cleaved fragment of CgPst2. Cell extracts (2 mg) of *wt* and *Cgyps1Δ* strains expressing CgPst2-SFB were incubated overnight with prewashed streptavidin beads at 4^ο^C, and centrifuged at 1500 rpm for 5 min. After 3 washes, beads were incubated with biotin solution (2 mg/ml) for 4 h at 4^ο^C, followed by incubation of the biotin solution with S-protein beads for 2 h at 4^ο^C. S-protein beads were washed, boiled in 2X SDS sample buffer and resolved on 18% SDS-PAGE. The gel was stained with Coomassie Brilliant Blue and destained. The band corresponding to the smaller cleaved fragment of ~16 kDa was cut and sent to the Sandor Life Sciences, Hyderabad, India (https://sandorlifesciences.co.in) for peptide mass fingerprinting analysis using MALDI Autoflex II TOF/TOF (Bruker Daltonics Inc. MA). The CgPst2 fragment was identified using parameters of peptide mass tolerance of ± 300 and fragment mass tolerance of ± 2 Da with maximum 2 missed cleavages.

### CgPst2 purification and size-exclusion chromatography analysis

The *CgPST2* (*CAGL0K11858*) ORF was cloned in EcoR1 and Xho1 sites in the pET28a(+) plasmid and transformed into *E*. *coli* BL21 (DE3) strain, followed by selection for kanamycin resistance (50 μg/ml). A purified transformant carrying *pET28a-6XHis-FLAG-CgPST2* was grown in LB medium containing kanamycin and induced with IPTG (0.5 mM) at 18^ο^C for 16 h. After cell collection via centrifugation at 8000 rpm for 10 min, cells were suspended in lysis buffer [50 mM Tris-HCl (pH 8.0), 150 mM NaCl and 10 mM Imidazole] and sonicated for 30 cycles of 30 sec ON/OFF (Biorupter, Diagenode). Cell lysates were centrifuged at 18000 rpm for 20 min, and the soluble recombinant CgPst2 protein was purified from the supernatant using the TALON metal affinity resin via affinity purification. Briefly, after overnight incubation of the supernatant with TALON beads at 4°C, this mix was added to the Polypropylene Column (Thermo Fisher Scientific #R64050), washed with the wash buffer (50 mM Tris-HCl (pH 8.0), 150 mM NaCl and 50 mM Imidazole), and the column-bound 6XHis-CgPst2 protein was eluted in the 250 mM imidazole-containing buffer. After removing imidazole through Amicon Ultra centrifugal filter units (3 kDa cut off), the protein purity was determined by SDS-PAGE and Western blot analysis. For size-exclusion chromatography, the column (1 cm radius and 48 cm height) was packed manually with Sephacryl S-200 matrix beads and run on the FPLC system (Bio-Rad). After pre-equilibrating the column with two column-volumes of buffer containing 50 mM Tris-HCl (pH 8.0) and 150 mM NaCl, about 300–500 μg of 6XHIS-FLAG-CgPst2 was applied to the injection ring with the volume of 300 μl. Samples were run with a flow rate of 0.22 ml/min for a total time of 5 h with at room temperature, and fractions of 0.5 ml were collected and analyzed with anti-His antibody. Blue dextran (1 mg/ml) was used to calculate the void volume. Marker proteins, thyroglobulin (A; 670 kDa), gammaglobulin (B; 158 kDa), ovalbumin (C; 44 kDa), myoglobin (D; 17kDa) and vitamin B12 (E; 1.35 kDa), of different sizes were used to calibrate the column. The calibration graph was plotted using the gel-phase distribution coefficient (Kav) against logarithm of the molecular weight (Log MW) of marker proteins. For each sample, at least two independent experiments were performed, and the molecular mass of eluted CgPst2 was calculated from the calibration plot using linear regression (R^2^ ≥ 0.96). CgPst2^*R174A*^, CgPst2-CΔ^*R174-F198*^ and CgPst2-CΔ^*S175-F198*^ were purified and analyzed in the similar fashion.

### Plasma membrane isolation

Log-phase *C*. *glabrata* cells (100 ml; 2.0 OD_600_) were harvested, washed with MQ water, suspended in homogenization buffer [50 mM Tris (pH 7.5), EDTA (2.5 mM)] and were lysed with glass beads. An aliquot of the supernatant was saved as the whole cell lysate fraction, and the remainder supernatant was subjected to ultracentrifugation (SW 41 Ti rotor) at 25000 rpm for 30 min at 4°C. The pellet was suspended in the suspension buffer [10 mM Tris (pH 7.5), 0.5 mM EDTA and 10% glycerol] containing protease inhibitors, followed by discontinuous sucrose gradient [43.5 and 53.5% (w/v)] ultracentrifugation at 35000 rpm for 5 h. The middle layer containing the plasma membrane was collected very carefully and again subjected to ultracentrifugation at 38000 rpm for 30 min. After discarding the supernatant, the plasma membrane pellet was suspended in suspension buffer containing protease inhibitors. Protein concentration in cell lysate and plasma membrane fractions was estimated by BCA method, and 60–200 μg samples were run on SDS-PAGE and probed with anti-CgYps1, anti-CgPst2, anti-Gapdh and anti-Pma1(Santa Cruz Biotechnology; # sc-33735) antibodies. Anti-Pma1 antibody, that recognizes the *C*. *glabrata* plasma membrane ATPase CgPma1 [13], was used to check the quality of plasma membrane preparation.

### Functional and statistical analysis

The Candida Genome Database (CGD)-GO Slim Mapper tool for BP (biological process) (http://www.candidagenome.org/cgi-bin/GO/goTermMapper) was used to functionally annotate *C*. *glabrata* genes. The FungiFun tool (https://elbe.hki-jena.de/fungifun/), with *C*. *glabrata* CBS138 as the reference strain, was used for GO functional enrichment analysis. The experimental data were statistically analysed using Student’s t-test. Mice data were analysed using Mann-Whitney U test. The p-values *p < 0.05; **p < 0.01; ***p < 0.001 and ****p *<* 0.0001 were used to determine statistical significance. Raw numerical data are presented in S6 Table.

Other protocols are provided in S1 Text.

## Supporting information

S1 Fig *C*. *glabrata* contains four flavodoxin-like proteins. **A.** Amino acid sequence identity (%) among Fld-LPs of *C*. *glabrata*, *S*. *cerevisiae* and *C*. *albicans*. Systematic ORF names of Fld-LPs, taken from Candida (http://www.candidagenome.org/) and Saccharomyces (https://www.yeastgenome.org/) Genome Databases, are indicated, with common gene names in brackets. Amino acid identity among protein sequences of these ORFs was determined using BLASTP analysis, with *C*. *glabrata* protein as a query sequence, against *C*. *glabrata* CBS138, *C*. *albicans* SC5314 (Assembly 19) and *S*. *cerevisiae* (S288C) reference strains. **B.** Schematic illustration of CgPst2, CgRfs1, CgPst3 and CgYcp4 protein domain structures, as predicted by the SMART tool 12 (http://smart.embl-heidelberg.de/smart/set_mode.cgi?NORMAL=1). Amino acids spanning the predicted domains are indicated. SMART tool predicted the FMN RED and Flavodoxin_2 domains. Proteins with Flavodoxin-2 domain include bacterial and eukaryotic NAD(P)H dehydrogenase (quinone) enzymes. These enzymes catalyze the NAD(P)H-dependent two-electron reduction of quinones and protect cells against damage by free radicals and reactive oxygen species. FMN RED domain is found in several flavoproteins such as FMN-dependent NADPH-azoreductases, which catalyze the reductive cleavage of azo bond in aromatic azo compounds to the corresponding amines, and NAD(P)H:quinone oxidoreductases, which reduce quinone to the hydroquinone state to prevent interaction of the semiquinone with O_2_ and production of superoxide. The figure is not drawn to scale.
(TIF)

S2 Fig Mutants lacking CgFld-LPs are not sensitive to thermal stress. **A.** Serial dilution spotting analysis showing growth of *C*. *glabrata* mutants deleted for single or multiple *CgFld-LP* genes on indicated stress conditions. Duroquinone (DQ) and cumene hydroperoxide (CHP) were used at a concentration of 450 μM and 40 μM, respectively. Plates were incubated at 30^ο^C unless indicated otherwise, and images were captured after 24 h for thermal stress (42^ο^C), and 48 h for DQ and CHP. The *Q-KO* strain lacks four flavodoxin-like proteins, CgPst2, CgRfs1, CgPst3 and CgYcp4. **B.** Serial dilution spotting analysis showing *CgPST2*-mediated complementation of menadione (MD; 95 μM) and benzoquinone (BQ; 4 mM) sensitivity of *Cgpst2Δ* and *Q-KO* strains. ‘*V*’ refers to empty vector. Plates were incubated at 30^ο^C, and images were captured after 24 h and 48 h for MD and BQ stresses, respectively.
(TIF)

S3 Fig The recombinant CgPst2 protein is non-functional. **A.** 6X-Histidine-FLAG-CgPst2 purification from *Escherichia coli*. The *E*. *coli* BL21 (DE3) strain transformant carrying *pET28a*^*(+)*^*-6XHIS-FLAG-CgPST2* plasmid was grown in LB medium, induced with IPTG (0.5 mM) at 18°C for 16 h, and cells were collected. After cell lysis, the recombinant CgPst2 protein was purified using TALON metal affinity resin *via* affinity purification. 30 μl eluates along with flow through were resolved on 12% SDS-PAGE, and stained with Coomassie Brilliant Blue. The black arrow marks CgPst2 band. Immunoblot analysis of these fractions with anti-His antibody (1:5000 dilution) is shown at the bottom. M, Protein Marker. **B.** NADH:quinone oxidoreductase activity measurement of recombinant CgPst2. 50 μg of purified CgPst2 was incubated for 1 h with FMN (100 μM) or FAD (100 μM) on ice, followed by addition of menadione (500 μM) in buffer containing Tris-HCl (10 mM; pH 7.4) and NaCl (150 mM). The reaction was started with NADH (500 μM) addition, and absorbance was recorded at 340 nm in a 1-cm-path-length quartz cuvette over a period of 60 seconds at 10 seconds interval on Spectramax M5 plate reader. The commercially available NAD(P)H:FMN oxidoreductase (1 Unit, Roche, # 10476480001) was taken as positive control. Blank contained no protein. Absorbance of the substrate NADH was considered as 100 at 0 h time point, and NADH oxidation was calculated by dividing the absorbance at each time point by 0 h absorbance, and multiplying the number by 100. Data represent mean ± SEM. Grouped multiple *t*-test was performed, with n = 3 to 5. **, p < 0.0021; ****, p < 0.0001. **C.** NADH:quinone oxidoreductase activity measurement in cell extracts of *wt*, *Cgpst2****Δ*** and *Q-KO* strains in enzymatic reaction mixtures that contained exogenously added FMN (50 **μ**M) or FAD (50 **μ**M). Data represent mean ± SD (n = 2–3). The *Q-KO* strain lacks four flavodoxin-like proteins, CgPst2, CgRfs1, CgPst3 and CgYcp4. **D.** NADH:quinone oxidoreductase activity measurement in extracts of *Cgpst2Δ* and *Q-KO* cells expressing *CgPST2*. *CgPST2* expression restored the activity deficit in *Cgpst2****Δ*** and *Q-KO* strains. Data represent mean ± SEM. Black and blue asterisks indicate statistically significant differences in activity of *QKO* and *Cgpst2Δ* samples, respectively, expressing *CgPST2*, compared to corresponding strains carrying vector. Grouped multiple *t*-test was performed, with n = 3. *, p < 0.0332; **, p < 0.0021.
(TIF)

S4 Fig The *Cgpst2Δyps1Δ* mutant exhibited no NADH:quinone oxidoreductase activity. NADH:quinone oxidoreductase activity measurement in extracts of *wt*, *Cgyps1Δ*, *Cgpst2Δ* and *Cgpst2Δyps1Δ* strains. Data represent mean ± SEM. Grouped multiple *t*-test was performed, with n = 3. Black and blue asterisks indicate statistically significant activity differences in *wt* strain, compared to *Cgpst2Δ* and *Cgpst2Δyps1Δ* strains, respectively. Brown and orange asterisks indicate statistically significant activity differences in *Cgyps1Δ* mutant compared to *Cgpst2Δ* and *Cgpst2Δyps1Δ* mutants, respectively. *, p < 0.0332; **, p < 0.0021; ***, p < 0.0002.
(TIF)

S5 Fig CgPst2 is localized at the plasma membrane. Confocal images illustrating cell membrane localization of CgPst2-GFP in log-phase cells of indicated strains grown in CAA or CAA medium containing menadione (90 μM; MD) for 90 min. DIC, Differential interference contrast. Bar = 2 μm.
(TIF)

S6 Fig Immunoblot analysis of CgPst2-CgYps1 interaction. Cell extracts of *wt* and *Cgyps1Δ* strains expressing CgPst2-SFB (3 mg protein) were incubated with streptavidin beads for 2 h at 4^ο^C and centrifuged at 2000 rpm for 5 min. Washed Beads were boiled in 2X SDS sample buffer, loaded on 8% (for CgYps1) and 12% (For SFB-tagged CgPst2) SDS-PAGE, and probed with anti-CgYps1 and anti-Flag antibodies, respectively. Please note that CgYps1 was not detectable in Input samples.
(TIF)

S7 Fig Multiple sequence alignment of 18 flavodoxin-like proteins. Protein sequences of Fld-LPs of *C*. *glabrata*, *S*. *cerevisiae*, *C*. *albicans*, *Aspergillus fumigatus*, *Paracoccidioides brasiliensis*, *Bacillus cereus*, *E*. *coli*, *Homo sapiens* and *Arabidopsis thaliana* were retrieved from the UniProt database (https://www.uniprot.org/uniprot/), and aligned and coloured using the Clustal Omega server (https://www.ebi.ac.uk/Tools/msa/clustalo/). Sequence alignment, corresponding to 127–198 amino acids, in the C-terminal region of CgPst2 is shown. The red arrow points to the conserved R174 residue in CgPst2. UniProtKB accession numbers are as follows: Q6FM13_CANGA (CgPst2), Q6FRB1_CANGA (CgRfs1), Q6FXR3_CANGA (CgPst3), Q6FS27_CANGA (CgYcp4), PST2_YEAST (*S*. *cerevisiae* Pst2), RFS1_YEAST (*S*. *cerevisiae* Rfs1), YCP4_YEAST (*S*. *cerevisiae* Ycp4), LOT6_YEAST (*S*. *cerevisiae* Lot6), A0A1D8PHR5_CANAL (*C*. *albicans* Pst1), Q59Y37_CANAL (*C*. *albicans* Pst2), A0A1D8PT02_CANAL (*C*. *albicans* Pst3), A0A1D8PT03_CANAL (*C*. *albicans* Ycp4), Q4WKD8_ASPFU (*Aspergillus fumigatus* Pst2), Q8X194_PARBR (*Paracoccidioides brasiliensis y20*), Q81G57_BACCR (*Bacillus cereus* Nrdl), NQOR_ECOLI (*E*. *coli* wrbA), NQO1_HUMAN (*Homo sapiens* NQO1), FQR1_ARATH (*Arabidopsis thaliana* FQR1).
(TIF)

S8 Fig Immunoblot analysis of *Cgpst2Δ* and *Cgpst2Δyps1Δ* cell extracts expressing alanine-substituted-*CgPST2-SFB* (*CgPST2*^*R174A*^), using anti-Flag antibody. CgGapdh was used as a loading control. M, Protein Marker.
(TIF)

S9 Fig CgYps1 proteins levels are elevated in response to MD treatment. **A.** Immunoblot analysis of CgPst2 expression in cell extracts of indicated *C*. *glabrata* strains to check the specificity of anti-CgPst2 polyclonal antibody. YPD-grown log-phase cells were lysed using glass beads, and 60 μg protein was resolved on 12% SDS-PAGE. After transferring proteins to the PVDF membrane, the blot was probed with antibody (1:1000 dilution) raised against purified CgPst2 protein in BALB/c mice. This mouse anti-CgPst2 sera yielded no and good signal in cell lysates of *Q-KO* (lacks all four flavodoxin-like proteins, CgPst2, CgRfs1, CgPst3 and CgYcp4), and *Cgpst2Δ* and Cg*rfs1Δ* mutants, respectively, indicating that the generated antibody binds to both CgPst2 and CgRfs1 proteins. CgGapdh was used as a loading control. **B.** qPCR-based determination of *CgPST2* transcript levels. Log-phase *T-KO* (*Cgrfs1Δpst3Δycp4Δ*) and *T-KOyps1Δ* (*Cgrfs1Δpst3Δycp4Δyps1Δ*) cells were either left untreated (UT) or treated with 90 μM menadione for 90 min (T). Data (mean ± SEM, *n =* 3) were normalized against the *CgACT1* mRNA control, and represent fold change in *CgPST2* expression in treated samples, compared to corresponding untreated samples (taken as 1.0). **C.** Immunoblot analysis of CgYps1 expression in cell extracts of untreated or menadione-(MD; 90 μM for 90 min)-treated log-phase cells of the *wt* strain, using anti-CgYps1 antibody. CgGapdh was used as a loading control. Of note, CgYps1 is predicted to be highly glycosylated which may account for diffuse nature of the CgYps1 band. Consistent with predicted posttranslational modifications, the CgYps1 band corresponds to about 135 kDa, as compared to the expected size of 64 kDa. **D.** Immunoblot analysis showing CgYps1-CgPst2 interaction in log-phase untreated and MD (90 μM for 90 min)-treated *Q-KO* cells expressing *CgPST2*. Immunoprecipitation was carried out with anti-CgYps1 antibody, and blots were probed with anti-CgYps1 and anti-CgPst2 antibodies. Untreated *penta-KO* strain expressing vector (V) was used as negative control. The green asterisk denotes non-specific band. The *Q-KO* strain lacks four flavodoxin-like proteins (Fld-LPs), CgPst2, CgRfs1, CgPst3 and CgYcp4, while the *penta-KO* strain lacks CgYps1 along with four Fld-LPs, CgPst2, CgRfs1, CgPst3 and CgYcp4.
(TIF)

S10 Fig CgYps1^*D91A*^ lacks proteolytic activity. **A.** The *Pichia pastoris* GS115 strain expressing secretory forms of either catalytically active (rCgYps1) and inactive CgYps1 (rCgYps1^*D91A*^) proteins was grown in the BMGY medium at 30°C for 24 h and incubated in the BMMY medium containing 2% methanol for 48 h. Methanol was used to induce the *AOX1* promoter, which drives the expression of CgYps1 proteins. 2% Methanol was added every 24 h to maintain the induction and expression of CgYps1 proteins. After 48 h, 1 ml supernatant was precipitated with 80% ammonium sulphate, pellet was suspended in PBS, and CgYps1 protein induction was checked by SDS PAGE. Induced proteins were purified by anion exchange chromatography using DEAE cellulose resin. The eluate fractions were resolved on 12% SDS-PAGE. The orange asterisk marks CgYps1 band. The protein samples were also probed with anti-His antibody (1:5000 dilution) and shown underneath the gel images. M, protein marker. **B.** CgYps1-mediated proteolysis of gelatin. The YNB-agar medium containing 1% gelatin was incubated either with 100 μg of purified proteins (rCgYps1 and rCgYps1^*D91A*^) or citrate buffer (pH 4.0; used for enzyme elution) for overnight at 37°C. The plate was stained with Coomassie Brilliant Blue G250 and imaged. The zone of hydrolysis was observed only with the CgYps1 enzyme. **C.** Proteolytic activity of DEAE cellulose-purified rCgYps1 and rCgYps1^*D91A*^ proteins (20 μg) was measured using 2.5% hemoglobin as a substrate in the citrate buffer (100 mM; pH 4.0) for 30 min at 37°C. TCA (10%) was added to stop the enzymatic reaction and precipitate the undigested hemoglobin and CgYps1 proteins. Cleaved peptides were collected from the supernatant and incubated with sodium bicarbonate (5 mM) and Folin-Ciocalteu reagent at 37°C for 30 min. The absorbance was read at 660 nm and converted into the μ moles of tyrosine released per mg of the protein. Control indicates the enzymatic reaction mixture containing buffer and substrate, but no CgYps1 protein. Data represent mean ± SEM of 4 independent samples. Of note, rCgYps1^*D91A*^ showed no proteolytic activity, indicating that Aspartate-91 in CgYps1 is required for enzyme activity. **, p < 0.01; unpaired two-tailed Student’s t test.
(TIF)

S11 Fig CgPst2 does not interact with CgYcp4. Immunoblot analysis showing no interaction between CgPst2 and CgYcp4-GFP. 6 mg precleared lysates of untreated and MD (90 uM for 90 min)-treated *T-KO* (*Cgrfs1Δpst3Δycp4Δ*) and *T-KOyps1Δ* (*Cgrfs1Δpst3Δycp4Δyps1Δ*) cells expressing *CgYCP4-GFP* were incubated with anti-GFP antibody-conjugated beads, followed by Western analysis with anti-GFP and anti-CgPst2 antibodies. The red and green arrows mark GFP and CgYcp4-GFP protein bands, respectively, in IP samples, while the green asterisk denotes non-specific band. Please note that Ycp4-GFP expression was not observed in Input samples, despite loading 200 μg protein.
(TIF)

S12 Fig CgYps1 is present in the plasma membrane fraction. Immunoblot analysis showing enrichment of CgYps1 and CgPst2 in the plasma membrane fraction of the *Q-KO* strain expressing *CgPST2*. Whole-cell lysates and plasma membrane fractions (60 μg and 200 μg for CgPst2 and CgYps1, respectively), prepared by glass bead lysis and sucrose gradient ultracentrifugation, respectively, were resolved on 4–20% polyacrylamide gradient, 12% polyacrylamide, 12% polyacrylamide and 12% polyacrylamide gels for CgYps1, CgPst2, CgPma1 and CgGapdh, respectively, and probed with anti-CgYps1, anti-CgPst2, anti-Pma1 and anti-Gapdh antibodies. The *penta-KO* strain was used as a negative control.
(TIF)

S13 Fig The *penta-KO* strain displays higher levels of CgPst2 than CgPst2^*R174A*^. **A.** Immunoblot analysis of CgPst2 levels in *Q-KO* and *penta-KO* strains expressing *CgPST2* or *CgPST2*^*R174A*^. Whole-cell extracts (60 μg), prepared by glass bead lysis, were resolved on 12% SDS-PAGE and probed with anti-CgPst2 and anti-Gapdh antibodies. The intensity of individual bands in 4 independent Western blots was quantified using the ImageJ densitometry software, and CgPst2 signal was normalized to the corresponding CgGapdh signal. Fold-change (mean ± SEM) in CgPst2 levels in *CgPST2*^*R174A*^-expressing cells, compared to *CgPST2*-expressing cells (considered as 1.0), is shown underneath the blot. *p* ≤ 0.01; paired two-tailed Student’s *t* test. The green asterisk indicates non-specific band. **B.** Immunoblot analysis of CgPst2 expression in *penta-KO* strains expressing *CgPST2Δ*^*R174-F198*^ or *CgPST2Δ*^*S175-F198*^. Whole-cell extracts (60 μg) of indicated strains were prepared by glass bead lysis and resolved on 18% SDS-PAGE for 4–5 h. The blots were probed with anti-CgPst2 and anti-Gapdh antibodies.
(TIF)

S14 Fig CgPst2-*C*Δ^*S175-F198*^ is non-functional. **A.** NADH:quinone oxidoreductase activity measurement in extracts of *Q-KO* expressing either *CgPST2* or *CgPST2-CΔ*^*S175-F198*^. Data represent mean ± SEM. Grouped multiple *t*-test was performed, with n = 3 to 4. *, p < 0.0332; **, p < 0.0021; ***, p < 0.0002; ****, p < 0.0001. The *Q-KO* strain lacks four flavodoxin-like proteins, CgPst2, CgRfs1, CgPst3 and CgYcp4. **B.** Immunoblot analysis of CgPst2 levels in *Q-KO* strains expressing *CgPST2* or *CgPST2-CΔ*^*S175-F198*^. The green asterisk denotes non-specific band.
(TIF)

S15 Fig The recombinant CgPst2^*R174A*^ and CgPst2-CΔ^*R174-F198*^ proteins do not exhibit NADH:quinone oxidoreductase activity. **A-C.** 6X-Histidine-FLAG-CgPst2^*R174A*^ **A.**, 6X-Histidine-FLAG-CgPst2-CΔ^*R174-F198*^ **B.** and 6X-Histidine-FLAG-CgPst2-CΔ^*S175-F198*^ **C.** protein purification from *E*. *coli*. The *E*. *coli* BL21 (DE3) strain transformants carrying *pET28a*^*(+)*^*-6XHIS-FLAG-CgPST2*^*R174A*^, *pET28a*^*(+)*^*-6XHIS-FLAG-CgPST2-CΔ*^*R174-F198*^ and *pET28a*^*(+)*^*-6XHIS-FLAG-CgPST2-CΔ*^*S175-F198*^ plasmids were grown in LB medium, induced with IPTG (0.5 mM) at 18^ο^C for 16 h, and cells were collected. After cell lysis, the recombinant proteins were purified using TALON metal affinity resin *via* affinity purification. 30 μl eluates, representing purified rCgPst2^*R174A*^ **A.** rCgPst2-CΔ^*R174-F198*^ **B.** and rCgPst2-CΔ^*S175-F198*^ **C.** proteins, along with the flow through, were resolved on 12% SDS-PAGE, and stained with Coomassie Brilliant Blue. The green arrow marks CgPst2 band. M, Protein Marker. **D-E.** NADH:quinone oxidoreductase activity measurement of the recombinant CgPst2^*R174A*^ **D.** and CgPst2-CΔ^*R174-F198*^ **E.** protein (250 μg) was measured, as described in the legend of S3B Fig. Blank contained no protein. Data represent mean ± SEM. Grouped multiple *t*-test was performed, with n = 3. Black and blue asterisks indicate statistically significant activity differences between the positive control [NAD(P)H:FMN oxidoreductase (1 Unit, Roche, # 10476480001)] and recombinant proteins incubated with FMN (100 μM) and FAD (100 μM), respectively. *, p < 0.0332. Please note that the enzymatic activity of the purified recombinant CgPst2-CΔ^*S175-F198*^ protein could not be determined, as it formed precipitates during the assay reaction. **F.** Size exclusion chromatogram of CgPst2-CΔ^*S175-F198*^. After loading 300 μg of purified 6XHIS-FLAG-CgPst2-CΔ^*S175-F198*^ protein on the Sephacryl S-200 column, protein elution profiles were determined using the absorbance values at 280 nm. The CgPst2-CΔ^*S175-F198*^ protein could not bind to the column, and appeared as aggregates in the column’s void volume, as determined by column calibration with blue dextran. AU, Arbitrary Units.
(TIF)

S16 Fig Native PAGE analysis showing CgPst2 tetramer band in *penta-KO* cells expressing *CgPST2-CΔ*^*R174-F198*^ and *CgPST2*^*R174*^. 300 μg whole cell lysates of *Q-KO*- expressing vector pRK74 (V) or *CgPST2*, and *penta-KO* expressing *CgPST2-CΔ*^*R174-F198*^, *CgPST2*^*R174A*^ and *CgPST2* were resolved in a discontinuous Tris-glycine buffer system under non-denaturing conditions, and probed with anti-CgPst2 antibody. The native protein molecular weight marker (M) was stained with coomassie brilliant blue, and is shown on the right side of the blot. The ponceau S-stained membrane is shown as loading control.
(TIF)

S17 Fig CgYps1 cleaves CgPst2. *In vitro* cleavage assay. The *Pichia pastoris* GS115 strain expressing either CgYps1 or CgYps1^*D91A*^ was induced with 2% methanol for 48 h, and pelleted down at 14000 rpm for 10 min at 4°C. The supernatants containing CgYps1 and CgYps1^*D91A*^ were filtered through a 0.22 μM filter (Millipore, USA), and concentrated using Amicon Ultra centrifugal filter unit (3 kDa cutoff). The retentate was precipitated with ammonium sulphate (100% saturation) for 10 min at 4°C, followed by centrifugation and pellet suspension in citrate buffer (pH 4.0). For the cleavage assay, 30 μg of r6XHis-Flag-CgPst2 was incubated with 60 μg of partially purified rCgYps1 or rCgYps1^*D91A*^ enzymes at 37°C for 4 h in citrate buffer (pH 4.0). Digested samples were run on 18% SDS-PAGE and probed with anti-His antibody. The assay samples containing both CgYps1 and CgPst2 displayed an additional faster migrating CgPst2 protein band (~ 24 kDa; marked with orange asterisk), probably representing the N-terminal fragment of the cleaved form of CgPst2, which was absent in reaction mixtures containing either CgPst2 alone or both CgPst2 and CgYps1^*D91A*^. These data indicate that the catalytically active CgYps1 can cleave CgPst2. Of note, we could not detect the small C-terminal-cleaved CgPst2 fragment, as hexa-His epitope is present at the N-terminus. To estimate the size difference between the cleaved and un-cleaved form of CgPst2, *E-coli*-purified full-length CgPst2 and CgPst2-CΔ^*R174-F198*^ (50 μg) were run on 18% SDS-PAGE as a control, and probed with anti-his antibody. M, Protein Marker.
(TIF)

S1 Table GO-SLIM Mapper and FungiFun analysis of 19 CgYps1-interacting proteins. (XLSX)

S2 Table Summary of menadione susceptibility of indicated *C*. *glabrata* strains expressing either vector, CgYps1, full-length CgPst2 or CgPst2 protein variants. (XLSX)

S3 Table List of strains used in the study. (XLSX)

S4 Table List of plasmids used in the study. (XLSX)

S5 Table List of primers used in the study. (XLSX)

S6 Table Raw numerical data underlying plotted graphs. (XLSX)

S1 Text Supporting protocols. (DOCX)
