# Supplementary figures and images for: An aspartyl protease-mediated cleavage regulates structure and function of a flavodoxin-like protein and aids oxidative stress survival
Source: PLoS Pathog. 2021 Feb 25;17(2):e1009355. doi: 10.1371/journal.ppat.1009355 (PMC7943015; doi:10.1371/journal.ppat.1009355)

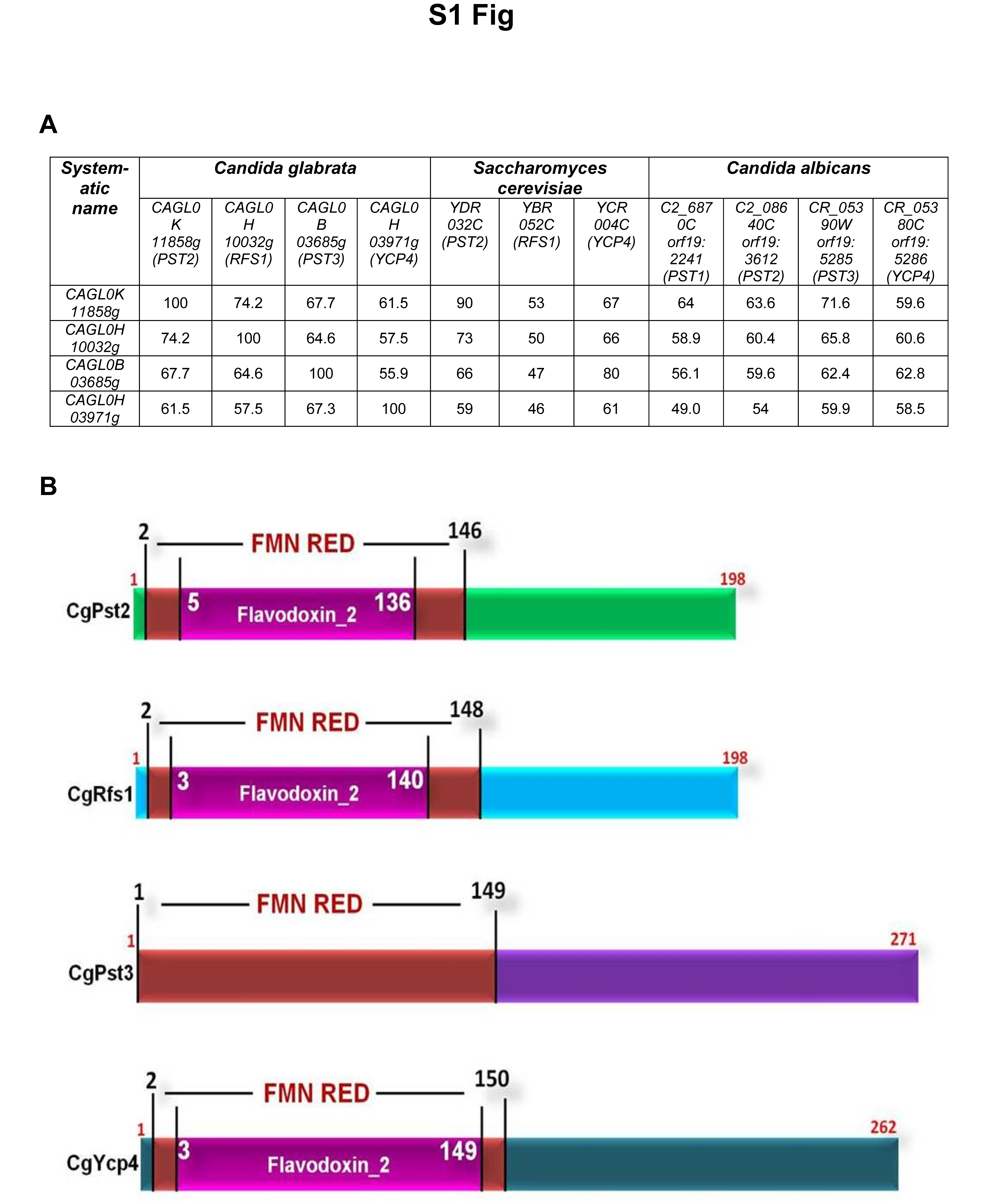

Supplement: S1 Fig — A. Amino acid sequence identity (%) among Fld-LPs of C. glabrata, S. cerevisiae and C. albicans. Systematic ORF names of Fld-LPs, taken from Candida (http://www.candidagenome.org/) and Saccharomyces (https://www.yeastgenome.org/) Genome Databases, are indicated, with common gene names in brackets. Amino acid identity among protein sequences of these ORFs was determined using BLASTP analysis, with C. glabrata protein as a query sequence, against C. glabrata CBS138, C. albicans SC5314 (Assembly 19) and S. cerevisiae (S288C) reference strains. B. Schematic illustration of CgPst2, CgRfs1, CgPst3 and CgYcp4 protein domain structures, as predicted by the SMART tool 12 (http://smart.embl-heidelberg.de/smart/set_mode.cgi?NORMAL=1). Amino acids spanning the predicted domains are indicated. SMART tool predicted the FMN RED and Flavodoxin_2 domains. Proteins with Flavodoxin-2 domain include bacterial and eukaryotic NAD(P)H dehydrogenase (quinone) enzymes. These enzymes catalyze the NAD(P)H-dependent two-electron reduction of quinones and protect cells against damage by free radicals and reactive oxygen species. FMN RED domain is found in several flavoproteins such as FMN-dependent NADPH-azoreductases, which catalyze the reductive cleavage of azo bond in aromatic azo compounds to the corresponding amines, and NAD(P)H:quinone oxidoreductases, which reduce quinone to the hydroquinone state to prevent interaction of the semiquinone with O2 and production of superoxide. The figure is not drawn to scale. (TIF) [file ppat.1009355.s001.tif]

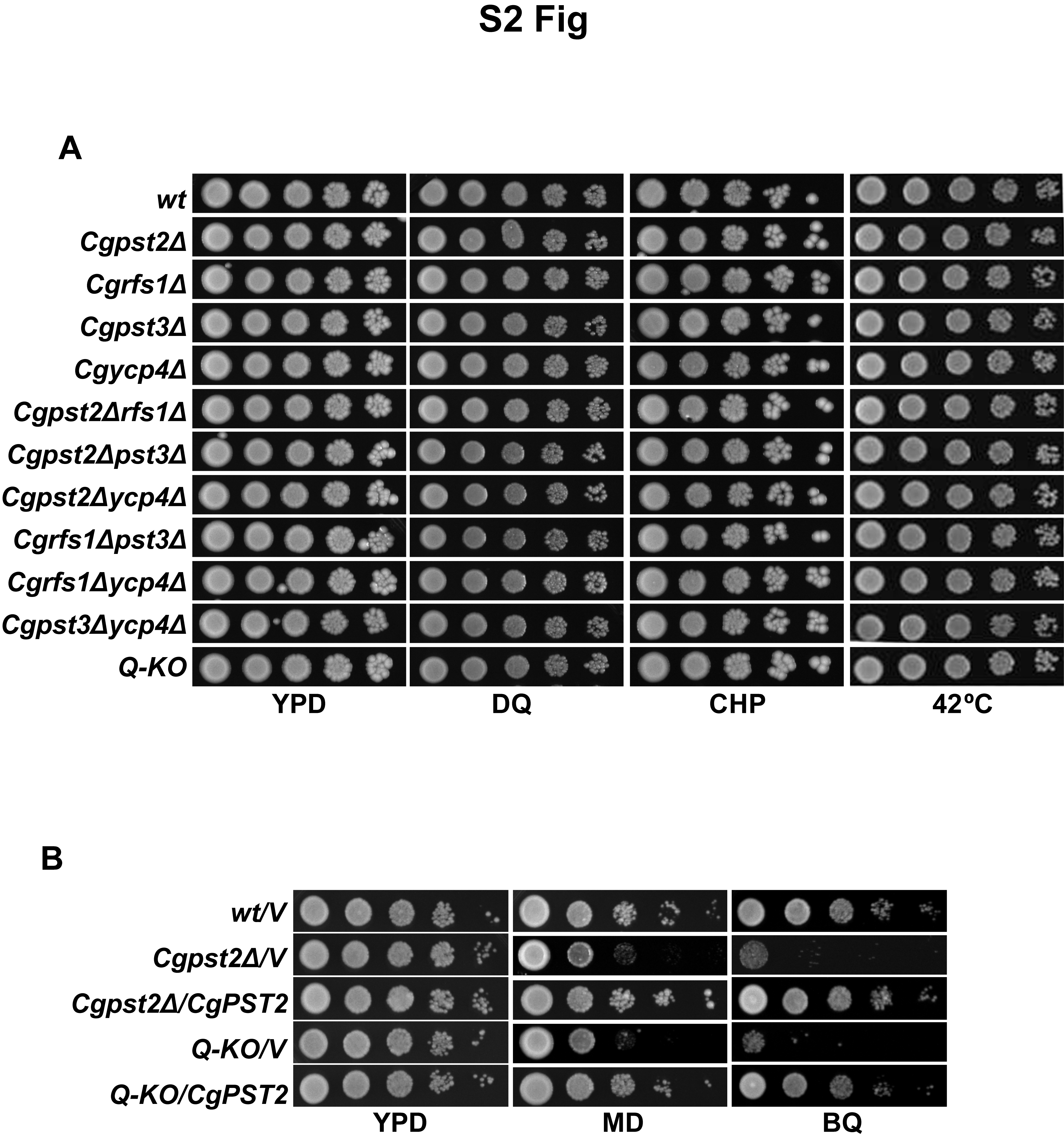

Supplement: S2 Fig — A. Serial dilution spotting analysis showing growth of C. glabrata mutants deleted for single or multiple CgFld-LP genes on indicated stress conditions. Duroquinone (DQ) and cumene hydroperoxide (CHP) were used at a concentration of 450 μM and 40 μM, respectively. Plates were incubated at 30οC unless indicated otherwise, and images were captured after 24 h for thermal stress (42οC), and 48 h for DQ and CHP. The Q-KO strain lacks four flavodoxin-like proteins, CgPst2, CgRfs1, CgPst3 and CgYcp4. B. Serial dilution spotting analysis showing CgPST2-mediated complementation of menadione (MD; 95 μM) and benzoquinone (BQ; 4 mM) sensitivity of Cgpst2Δ and Q-KO strains. ‘V’ refers to empty vector. Plates were incubated at 30οC, and images were captured after 24 h and 48 h for MD and BQ stresses, respectively. (TIF) [file ppat.1009355.s002.tif]

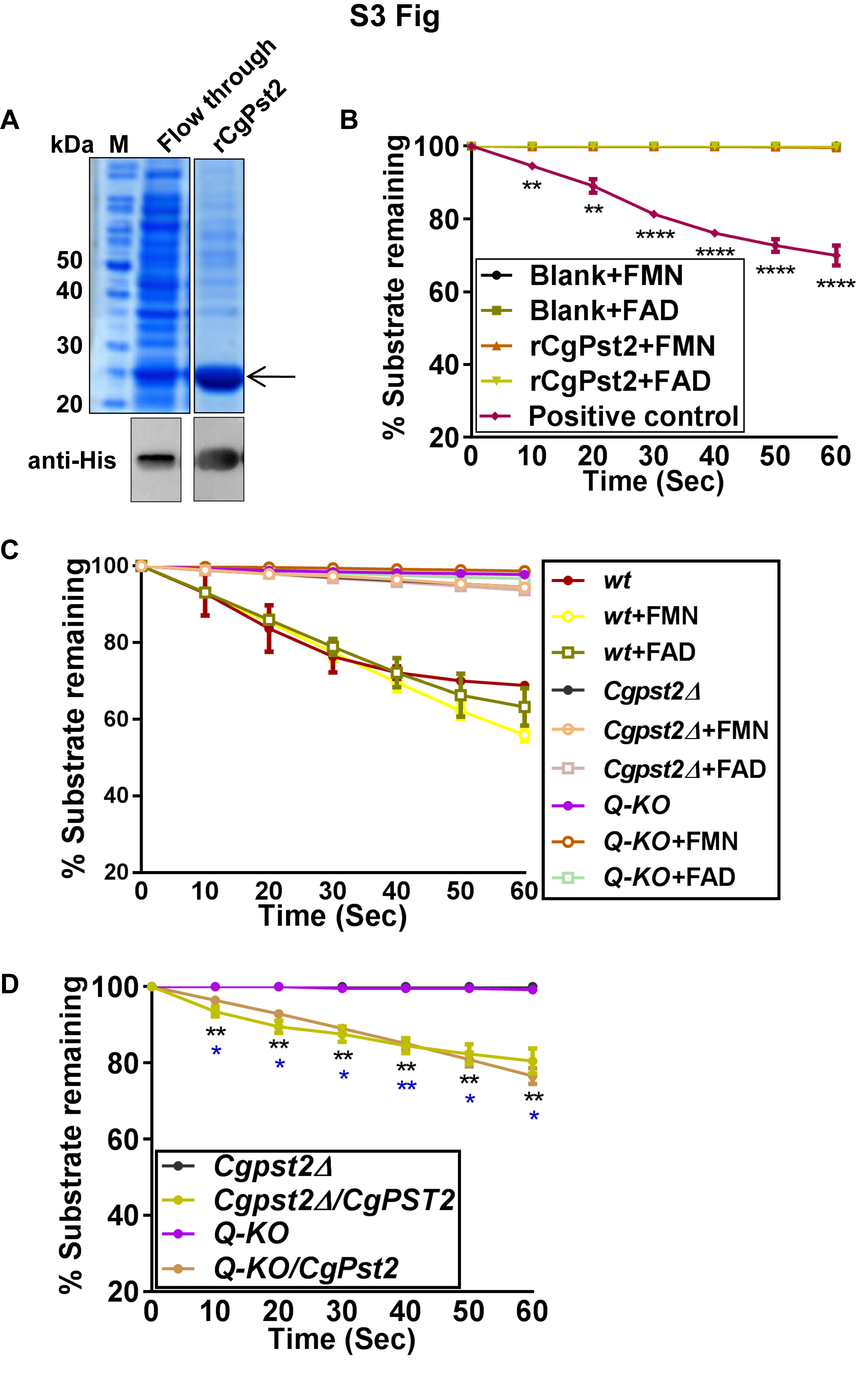

Supplement: S3 Fig — A. 6X-Histidine-FLAG-CgPst2 purification from Escherichia coli. The E. coli BL21 (DE3) strain transformant carrying pET28a(+)-6XHIS-FLAG-CgPST2 plasmid was grown in LB medium, induced with IPTG (0.5 mM) at 18°C for 16 h, and cells were collected. After cell lysis, the recombinant CgPst2 protein was purified using TALON metal affinity resin via affinity purification. 30 μl eluates along with flow through were resolved on 12% SDS-PAGE, and stained with Coomassie Brilliant Blue. The black arrow marks CgPst2 band. Immunoblot analysis of these fractions with anti-His antibody (1:5000 dilution) is shown at the bottom. M, Protein Marker. B. NADH:quinone oxidoreductase activity measurement of recombinant CgPst2. 50 μg of purified CgPst2 was incubated for 1 h with FMN (100 μM) or FAD (100 μM) on ice, followed by addition of menadione (500 μM) in buffer containing Tris-HCl (10 mM; pH 7.4) and NaCl (150 mM). The reaction was started with NADH (500 μM) addition, and absorbance was recorded at 340 nm in a 1-cm-path-length quartz cuvette over a period of 60 seconds at 10 seconds interval on Spectramax M5 plate reader. The commercially available NAD(P)H:FMN oxidoreductase (1 Unit, Roche, # 10476480001) was taken as positive control. Blank contained no protein. Absorbance of the substrate NADH was considered as 100 at 0 h time point, and NADH oxidation was calculated by dividing the absorbance at each time point by 0 h absorbance, and multiplying the number by 100. Data represent mean ± SEM. Grouped multiple t-test was performed, with n = 3 to 5. **, p < 0.0021; ****, p < 0.0001. C. NADH:quinone oxidoreductase activity measurement in cell extracts of wt, Cgpst2Δ and Q-KO strains in enzymatic reaction mixtures that contained exogenously added FMN (50 μM) or FAD (50 μM). Data represent mean ± SD (n = 2–3). The Q-KO strain lacks four flavodoxin-like proteins, CgPst2, CgRfs1, CgPst3 and CgYcp4. D. NADH:quinone oxidoreductase activity measurement in extracts of Cgpst2Δ and Q-KO cells ex [file ppat.1009355.s003.tif]

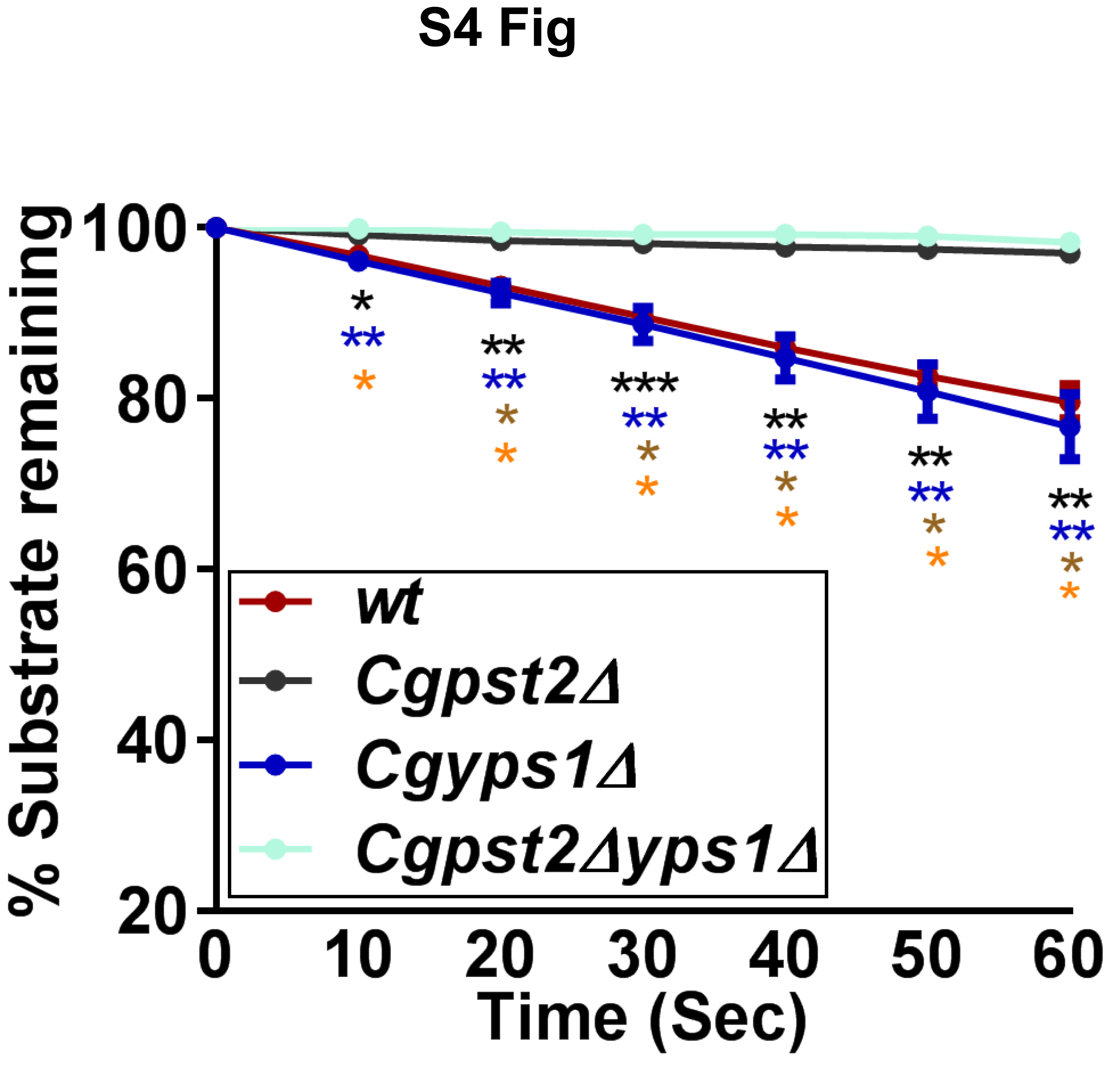

Supplement: S4 Fig — NADH:quinone oxidoreductase activity measurement in extracts of wt, Cgyps1Δ, Cgpst2Δ and Cgpst2Δyps1Δ strains. Data represent mean ± SEM. Grouped multiple t-test was performed, with n = 3. Black and blue asterisks indicate statistically significant activity differences in wt strain, compared to Cgpst2Δ and Cgpst2Δyps1Δ strains, respectively. Brown and orange asterisks indicate statistically significant activity differences in Cgyps1Δ mutant compared to Cgpst2Δ and Cgpst2Δyps1Δ mutants, respectively. *, p < 0.0332; **, p < 0.0021; ***, p < 0.0002. (TIF) [file ppat.1009355.s004.tif]

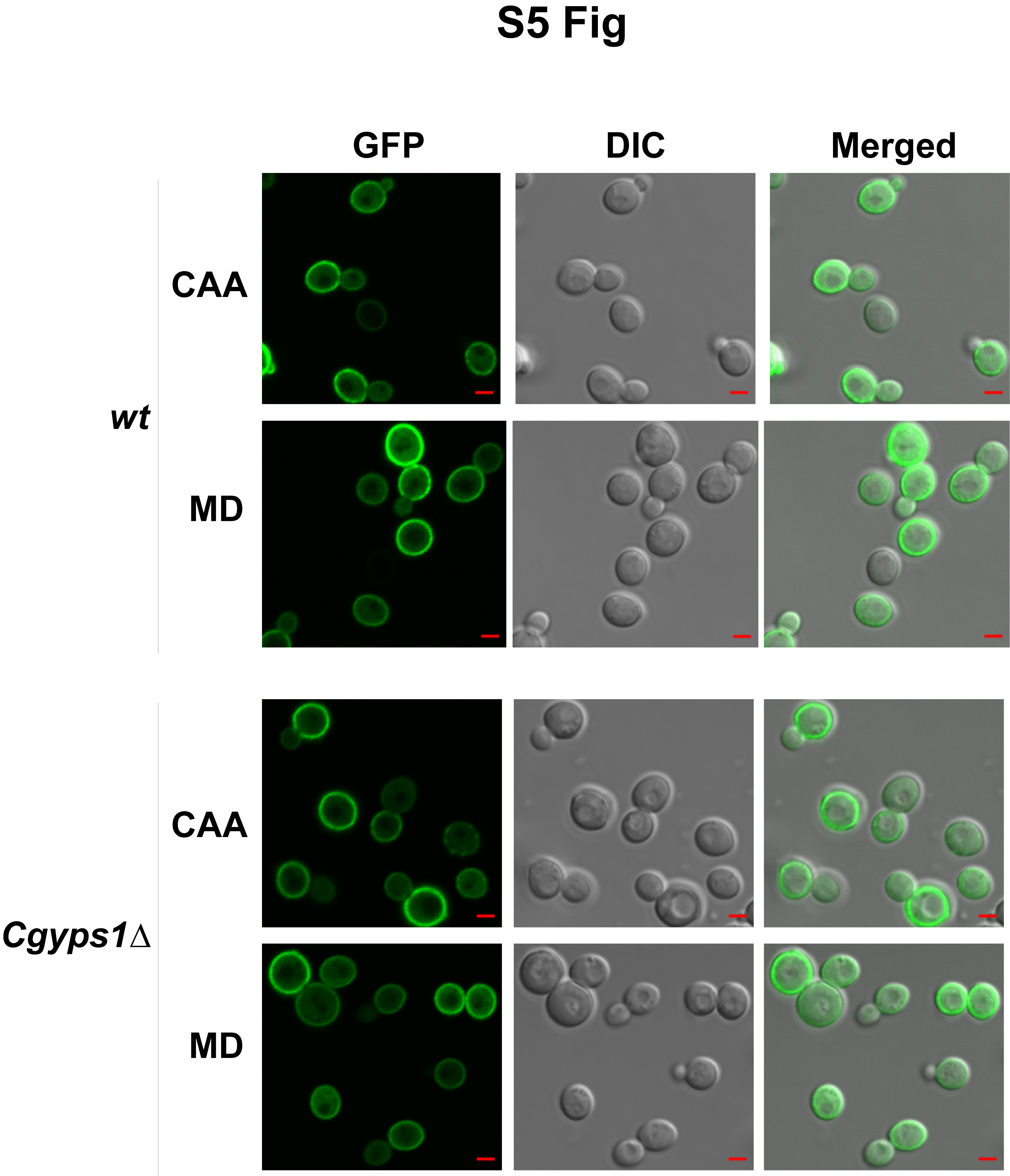

Supplement: S5 Fig — Confocal images illustrating cell membrane localization of CgPst2-GFP in log-phase cells of indicated strains grown in CAA or CAA medium containing menadione (90 μM; MD) for 90 min. DIC, Differential interference contrast. Bar = 2 μm. (TIF) [file ppat.1009355.s005.tif]

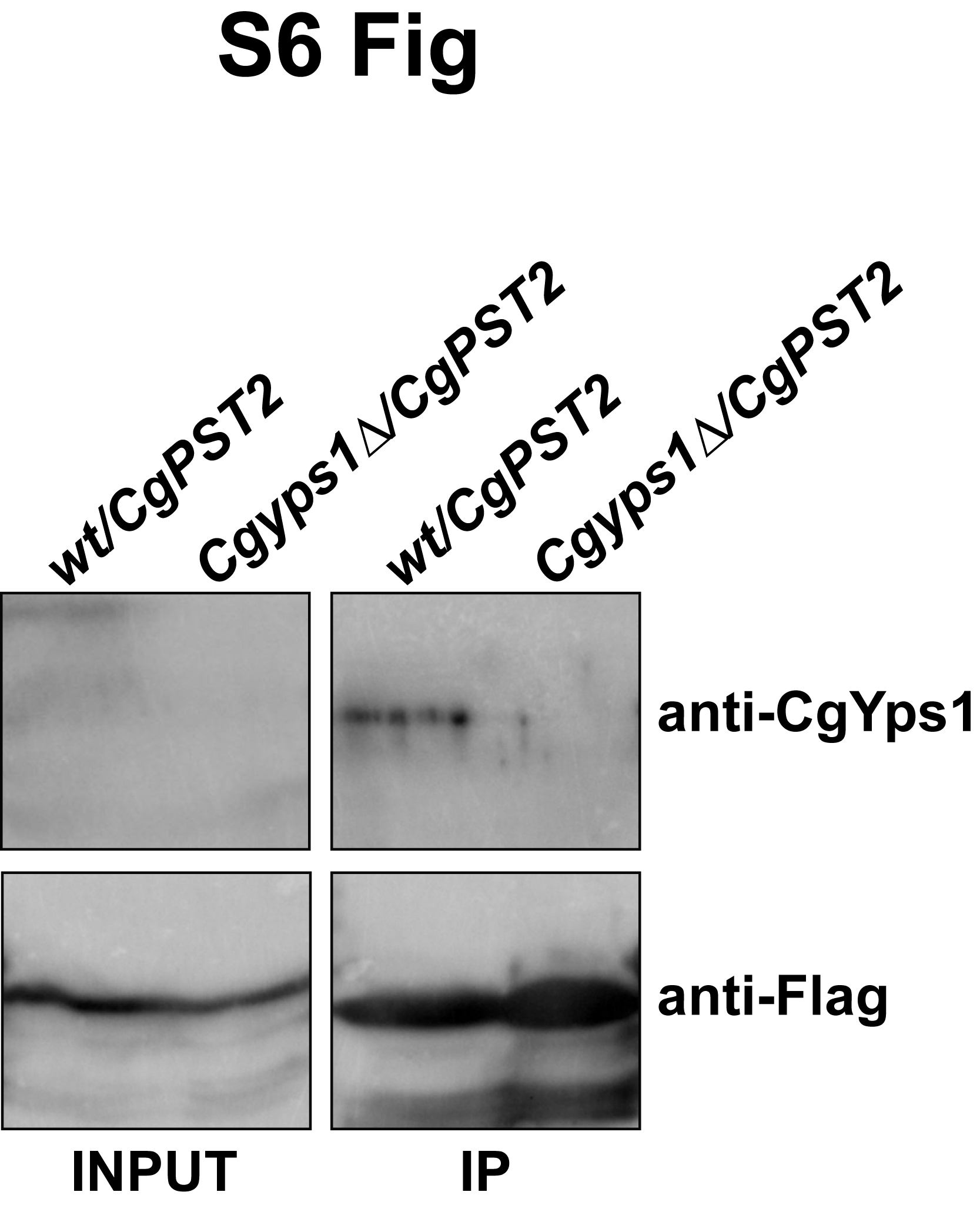

Supplement: S6 Fig — Cell extracts of wt and Cgyps1Δ strains expressing CgPst2-SFB (3 mg protein) were incubated with streptavidin beads for 2 h at 4οC and centrifuged at 2000 rpm for 5 min. Washed Beads were boiled in 2X SDS sample buffer, loaded on 8% (for CgYps1) and 12% (For SFB-tagged CgPst2) SDS-PAGE, and probed with anti-CgYps1 and anti-Flag antibodies, respectively. Please note that CgYps1 was not detectable in Input samples. (TIF) [file ppat.1009355.s006.tif]

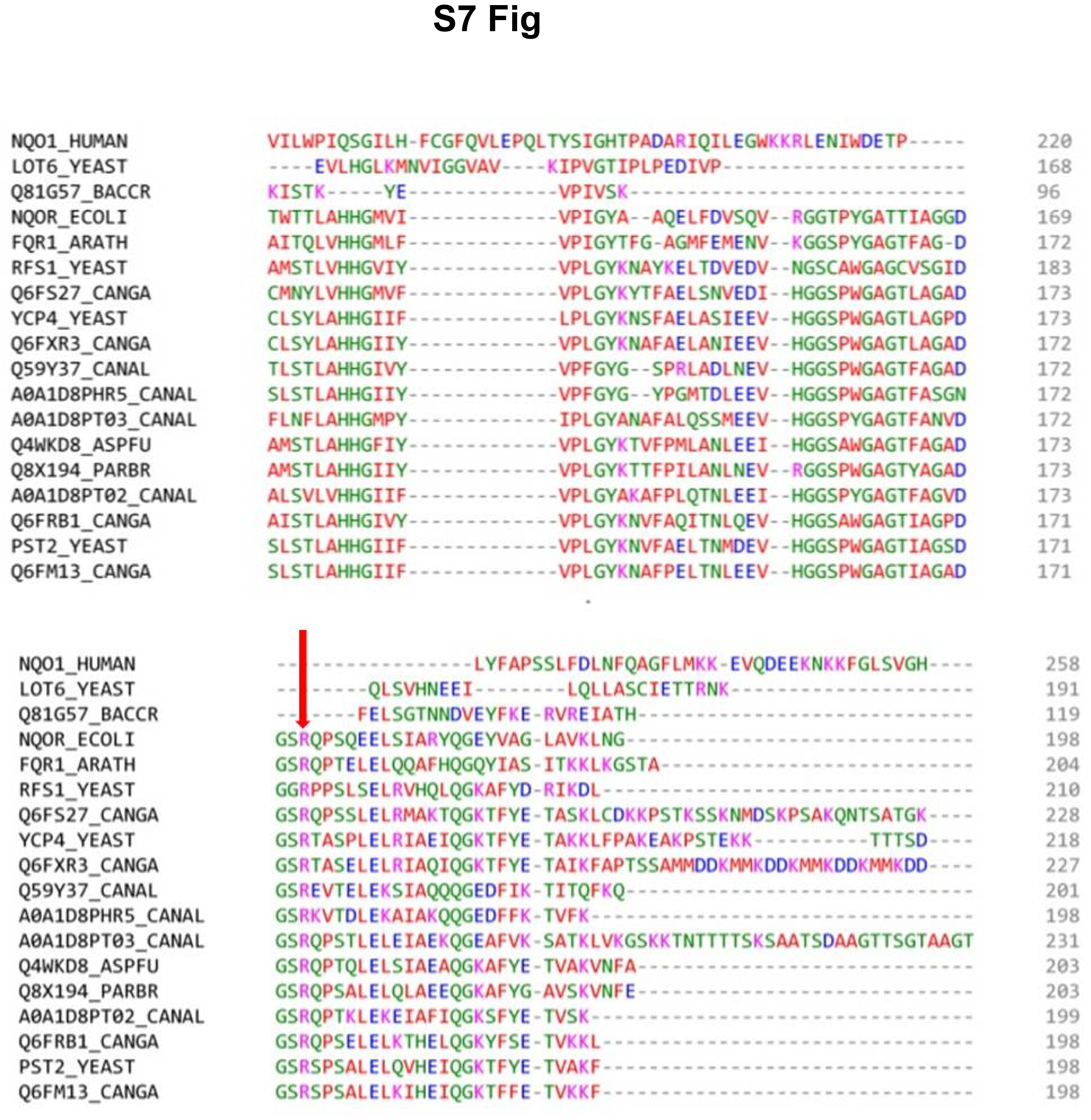

Supplement: S7 Fig — Protein sequences of Fld-LPs of C. glabrata, S. cerevisiae, C. albicans, Aspergillus fumigatus, Paracoccidioides brasiliensis, Bacillus cereus, E. coli, Homo sapiens and Arabidopsis thaliana were retrieved from the UniProt database (https://www.uniprot.org/uniprot/), and aligned and coloured using the Clustal Omega server (https://www.ebi.ac.uk/Tools/msa/clustalo/). Sequence alignment, corresponding to 127–198 amino acids, in the C-terminal region of CgPst2 is shown. The red arrow points to the conserved R174 residue in CgPst2. UniProtKB accession numbers are as follows: Q6FM13_CANGA (CgPst2), Q6FRB1_CANGA (CgRfs1), Q6FXR3_CANGA (CgPst3), Q6FS27_CANGA (CgYcp4), PST2_YEAST (S. cerevisiae Pst2), RFS1_YEAST (S. cerevisiae Rfs1), YCP4_YEAST (S. cerevisiae Ycp4), LOT6_YEAST (S. cerevisiae Lot6), A0A1D8PHR5_CANAL (C. albicans Pst1), Q59Y37_CANAL (C. albicans Pst2), A0A1D8PT02_CANAL (C. albicans Pst3), A0A1D8PT03_CANAL (C. albicans Ycp4), Q4WKD8_ASPFU (Aspergillus fumigatus Pst2), Q8X194_PARBR (Paracoccidioides brasiliensis y20), Q81G57_BACCR (Bacillus cereus Nrdl), NQOR_ECOLI (E. coli wrbA), NQO1_HUMAN (Homo sapiens NQO1), FQR1_ARATH (Arabidopsis thaliana FQR1). (TIF) [file ppat.1009355.s007.tif]

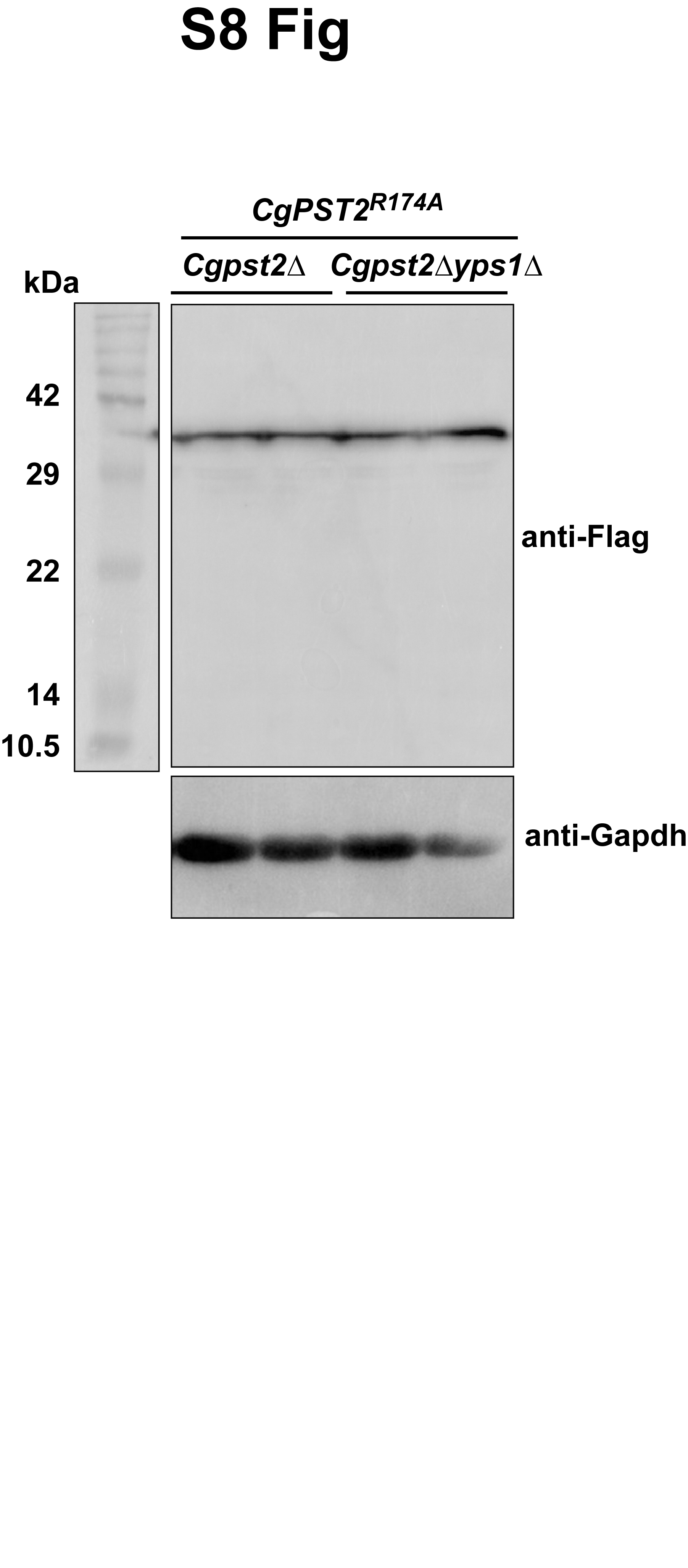

Supplement: S8 Fig — CgGapdh was used as a loading control. M, Protein Marker. (TIF) [file ppat.1009355.s008.tif]

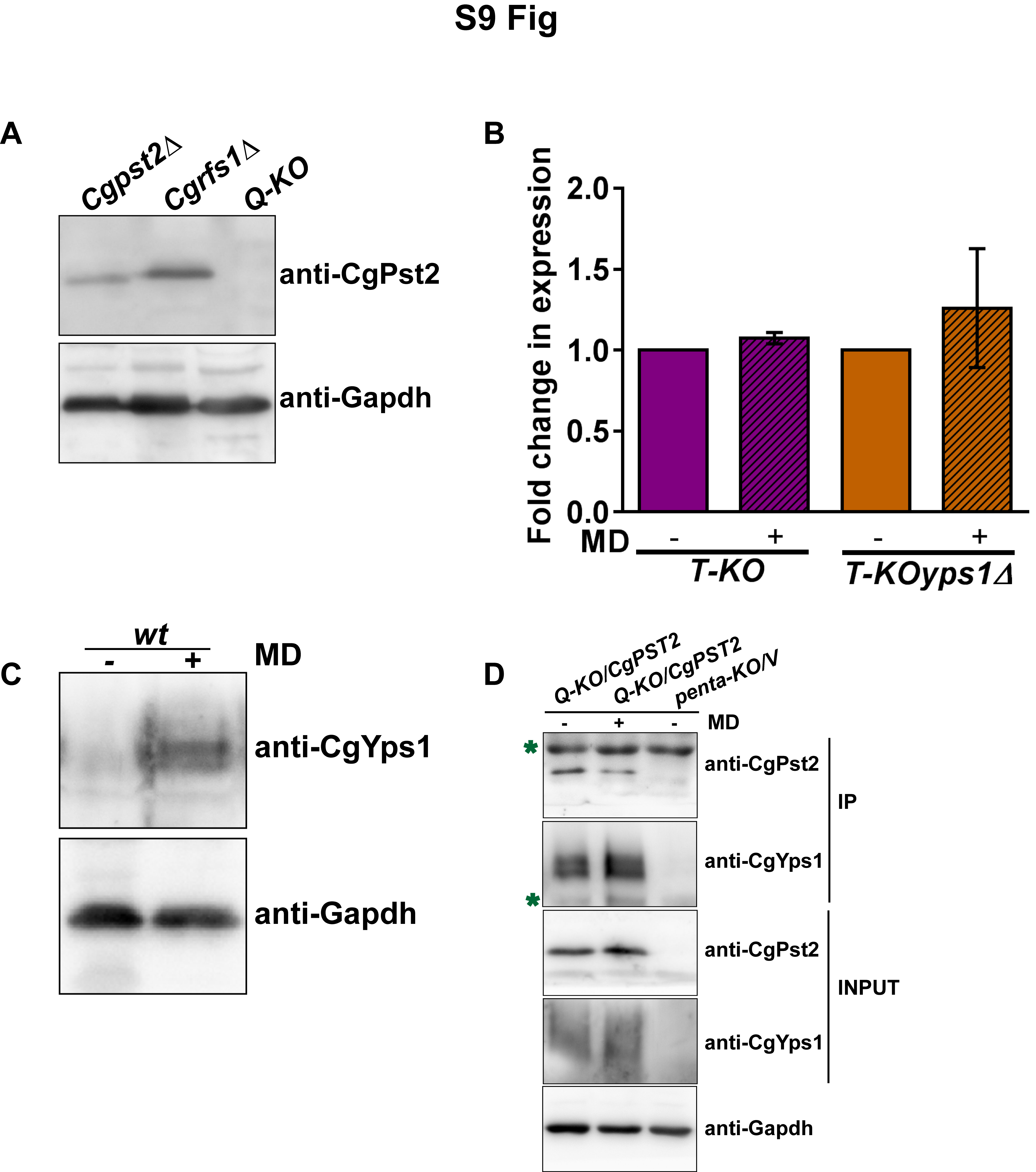

Supplement: S9 Fig — A. Immunoblot analysis of CgPst2 expression in cell extracts of indicated C. glabrata strains to check the specificity of anti-CgPst2 polyclonal antibody. YPD-grown log-phase cells were lysed using glass beads, and 60 μg protein was resolved on 12% SDS-PAGE. After transferring proteins to the PVDF membrane, the blot was probed with antibody (1:1000 dilution) raised against purified CgPst2 protein in BALB/c mice. This mouse anti-CgPst2 sera yielded no and good signal in cell lysates of Q-KO (lacks all four flavodoxin-like proteins, CgPst2, CgRfs1, CgPst3 and CgYcp4), and Cgpst2Δ and Cgrfs1Δ mutants, respectively, indicating that the generated antibody binds to both CgPst2 and CgRfs1 proteins. CgGapdh was used as a loading control. B. qPCR-based determination of CgPST2 transcript levels. Log-phase T-KO (Cgrfs1Δpst3Δycp4Δ) and T-KOyps1Δ (Cgrfs1Δpst3Δycp4Δyps1Δ) cells were either left untreated (UT) or treated with 90 μM menadione for 90 min (T). Data (mean ± SEM, n = 3) were normalized against the CgACT1 mRNA control, and represent fold change in CgPST2 expression in treated samples, compared to corresponding untreated samples (taken as 1.0). C. Immunoblot analysis of CgYps1 expression in cell extracts of untreated or menadione-(MD; 90 μM for 90 min)-treated log-phase cells of the wt strain, using anti-CgYps1 antibody. CgGapdh was used as a loading control. Of note, CgYps1 is predicted to be highly glycosylated which may account for diffuse nature of the CgYps1 band. Consistent with predicted posttranslational modifications, the CgYps1 band corresponds to about 135 kDa, as compared to the expected size of 64 kDa. D. Immunoblot analysis showing CgYps1-CgPst2 interaction in log-phase untreated and MD (90 μM for 90 min)-treated Q-KO cells expressing CgPST2. Immunoprecipitation was carried out with anti-CgYps1 antibody, and blots were probed with anti-CgYps1 and anti-CgPst2 antibodies. Untreated penta-KO strain expressing vector (V) was used as negative control. The green [file ppat.1009355.s009.tif]

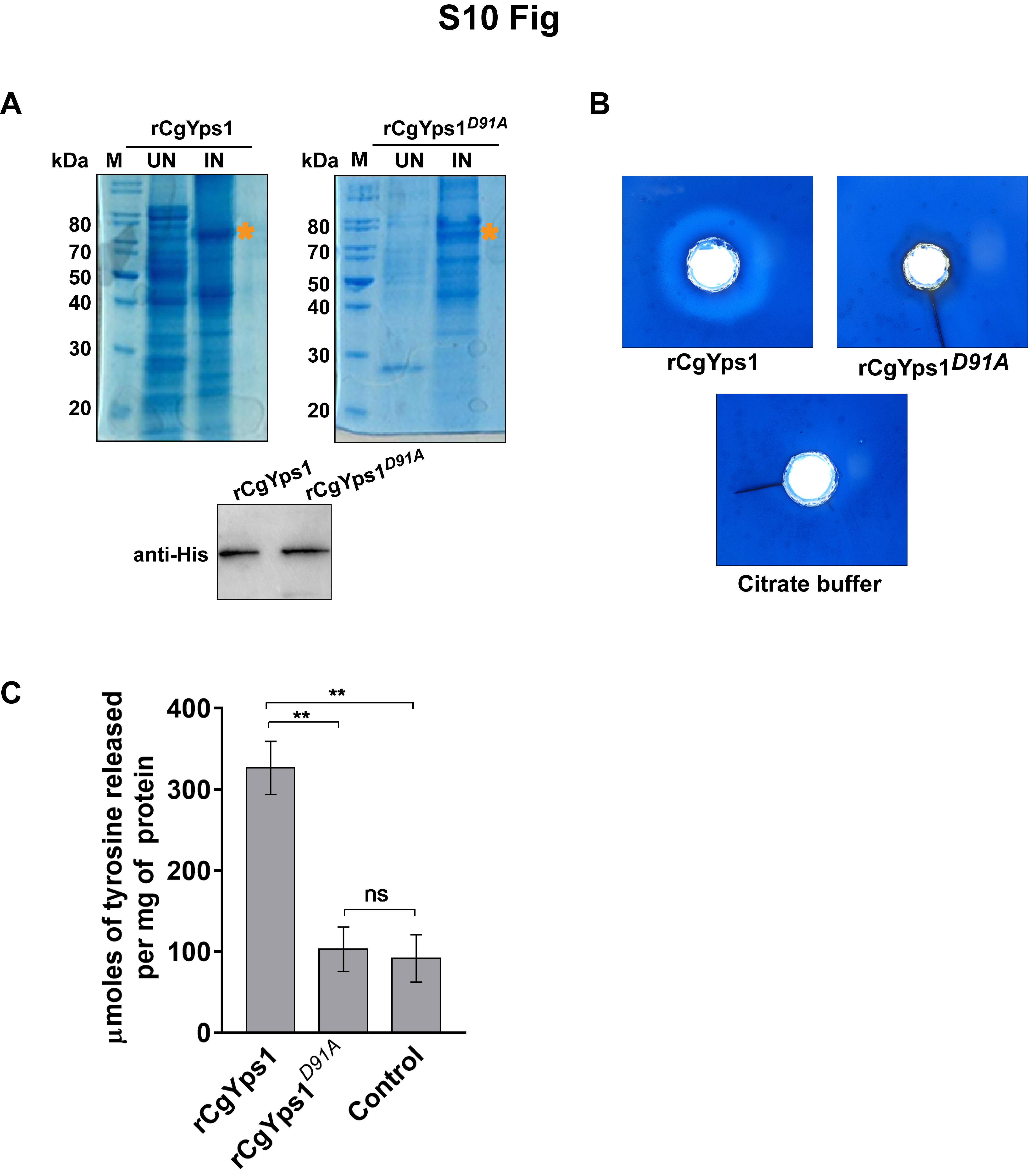

Supplement: S10 Fig — A. The Pichia pastoris GS115 strain expressing secretory forms of either catalytically active (rCgYps1) and inactive CgYps1 (rCgYps1D91A) proteins was grown in the BMGY medium at 30°C for 24 h and incubated in the BMMY medium containing 2% methanol for 48 h. Methanol was used to induce the AOX1 promoter, which drives the expression of CgYps1 proteins. 2% Methanol was added every 24 h to maintain the induction and expression of CgYps1 proteins. After 48 h, 1 ml supernatant was precipitated with 80% ammonium sulphate, pellet was suspended in PBS, and CgYps1 protein induction was checked by SDS PAGE. Induced proteins were purified by anion exchange chromatography using DEAE cellulose resin. The eluate fractions were resolved on 12% SDS-PAGE. The orange asterisk marks CgYps1 band. The protein samples were also probed with anti-His antibody (1:5000 dilution) and shown underneath the gel images. M, protein marker. B. CgYps1-mediated proteolysis of gelatin. The YNB-agar medium containing 1% gelatin was incubated either with 100 μg of purified proteins (rCgYps1 and rCgYps1D91A) or citrate buffer (pH 4.0; used for enzyme elution) for overnight at 37°C. The plate was stained with Coomassie Brilliant Blue G250 and imaged. The zone of hydrolysis was observed only with the CgYps1 enzyme. C. Proteolytic activity of DEAE cellulose-purified rCgYps1 and rCgYps1D91A proteins (20 μg) was measured using 2.5% hemoglobin as a substrate in the citrate buffer (100 mM; pH 4.0) for 30 min at 37°C. TCA (10%) was added to stop the enzymatic reaction and precipitate the undigested hemoglobin and CgYps1 proteins. Cleaved peptides were collected from the supernatant and incubated with sodium bicarbonate (5 mM) and Folin-Ciocalteu reagent at 37°C for 30 min. The absorbance was read at 660 nm and converted into the μ moles of tyrosine released per mg of the protein. Control indicates the enzymatic reaction mixture containing buffer and substrate, but no CgYps1 protein. Data represent mean ± SEM of [file ppat.1009355.s010.tif]

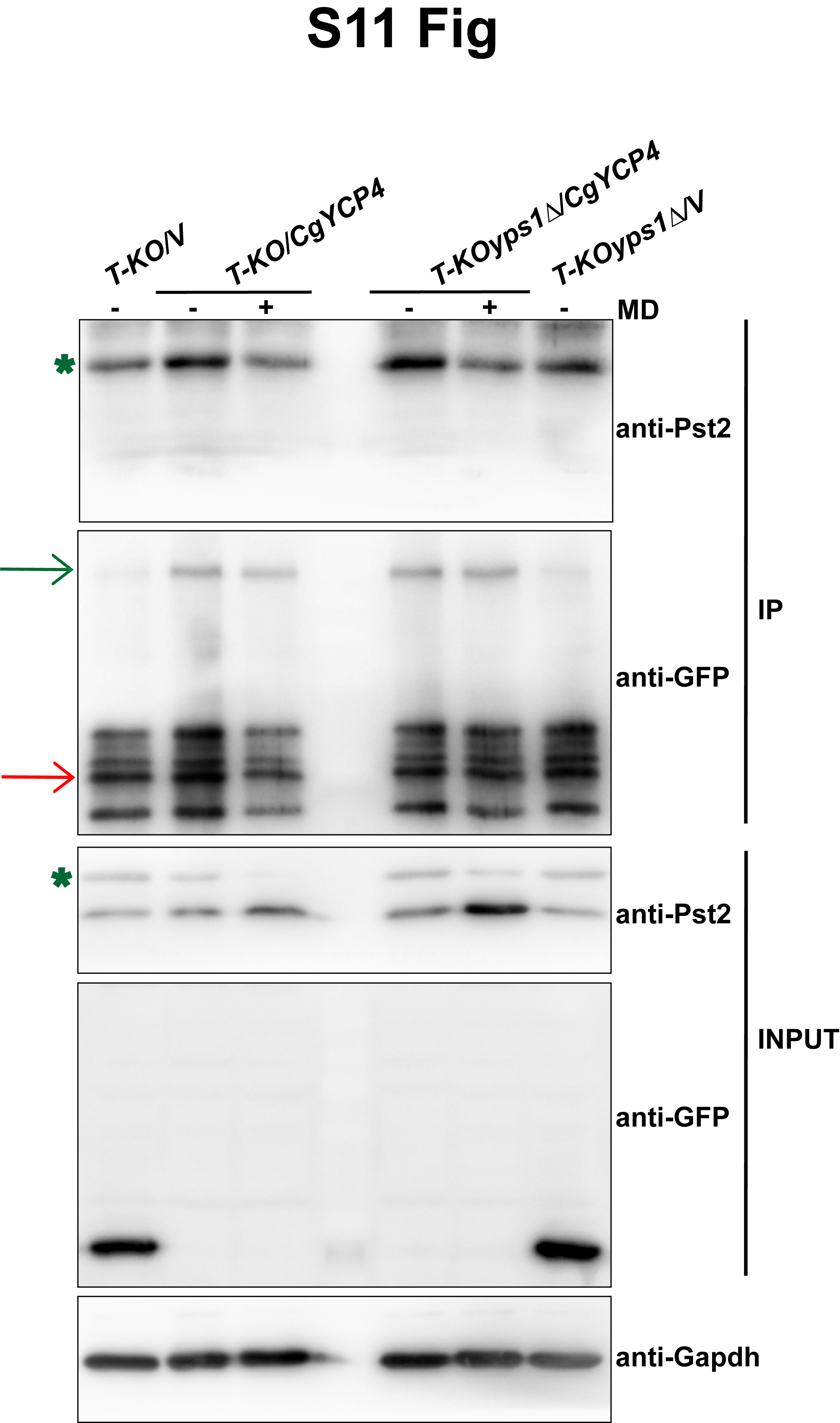

Supplement: S11 Fig — Immunoblot analysis showing no interaction between CgPst2 and CgYcp4-GFP. 6 mg precleared lysates of untreated and MD (90 uM for 90 min)-treated T-KO (Cgrfs1Δpst3Δycp4Δ) and T-KOyps1Δ (Cgrfs1Δpst3Δycp4Δyps1Δ) cells expressing CgYCP4-GFP were incubated with anti-GFP antibody-conjugated beads, followed by Western analysis with anti-GFP and anti-CgPst2 antibodies. The red and green arrows mark GFP and CgYcp4-GFP protein bands, respectively, in IP samples, while the green asterisk denotes non-specific band. Please note that Ycp4-GFP expression was not observed in Input samples, despite loading 200 μg protein. (TIF) [file ppat.1009355.s011.tif]

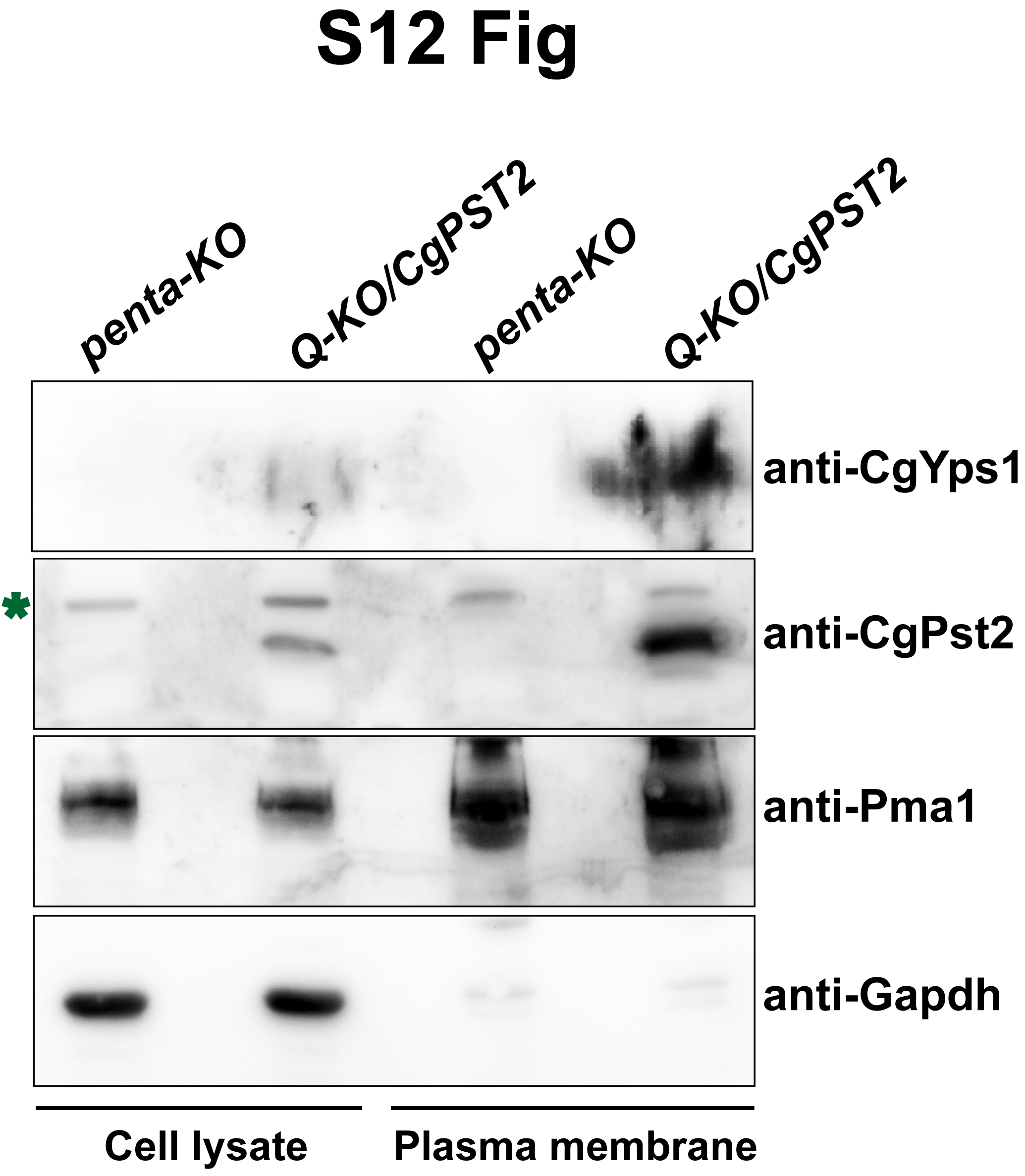

Supplement: S12 Fig — Immunoblot analysis showing enrichment of CgYps1 and CgPst2 in the plasma membrane fraction of the Q-KO strain expressing CgPST2. Whole-cell lysates and plasma membrane fractions (60 μg and 200 μg for CgPst2 and CgYps1, respectively), prepared by glass bead lysis and sucrose gradient ultracentrifugation, respectively, were resolved on 4–20% polyacrylamide gradient, 12% polyacrylamide, 12% polyacrylamide and 12% polyacrylamide gels for CgYps1, CgPst2, CgPma1 and CgGapdh, respectively, and probed with anti-CgYps1, anti-CgPst2, anti-Pma1 and anti-Gapdh antibodies. The penta-KO strain was used as a negative control. (TIF) [file ppat.1009355.s012.tif]

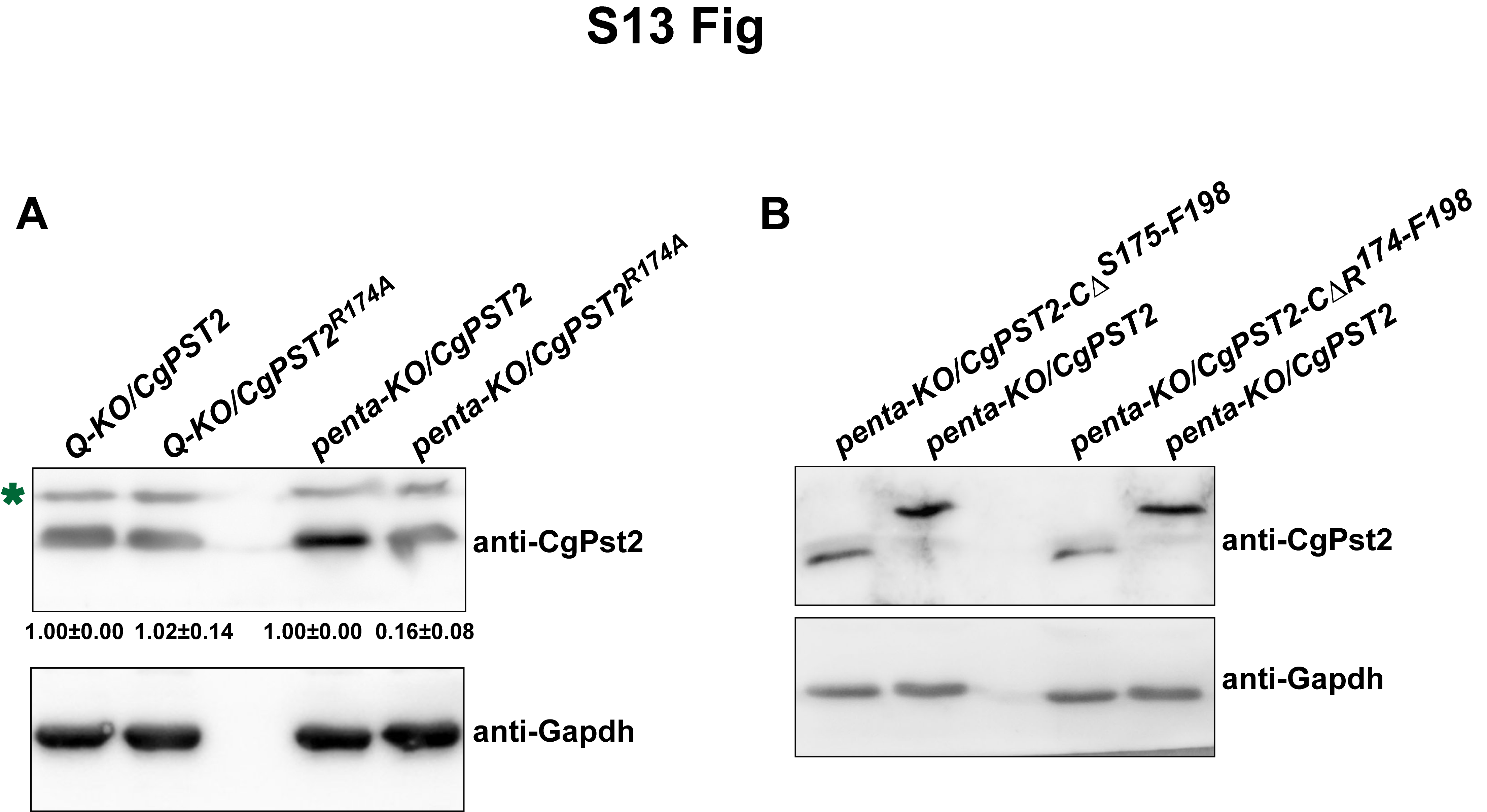

Supplement: S13 Fig — A. Immunoblot analysis of CgPst2 levels in Q-KO and penta-KO strains expressing CgPST2 or CgPST2R174A. Whole-cell extracts (60 μg), prepared by glass bead lysis, were resolved on 12% SDS-PAGE and probed with anti-CgPst2 and anti-Gapdh antibodies. The intensity of individual bands in 4 independent Western blots was quantified using the ImageJ densitometry software, and CgPst2 signal was normalized to the corresponding CgGapdh signal. Fold-change (mean ± SEM) in CgPst2 levels in CgPST2R174A-expressing cells, compared to CgPST2-expressing cells (considered as 1.0), is shown underneath the blot. p ≤ 0.01; paired two-tailed Student’s t test. The green asterisk indicates non-specific band. B. Immunoblot analysis of CgPst2 expression in penta-KO strains expressing CgPST2ΔR174-F198 or CgPST2ΔS175-F198. Whole-cell extracts (60 μg) of indicated strains were prepared by glass bead lysis and resolved on 18% SDS-PAGE for 4–5 h. The blots were probed with anti-CgPst2 and anti-Gapdh antibodies. (TIF) [file ppat.1009355.s013.tif]

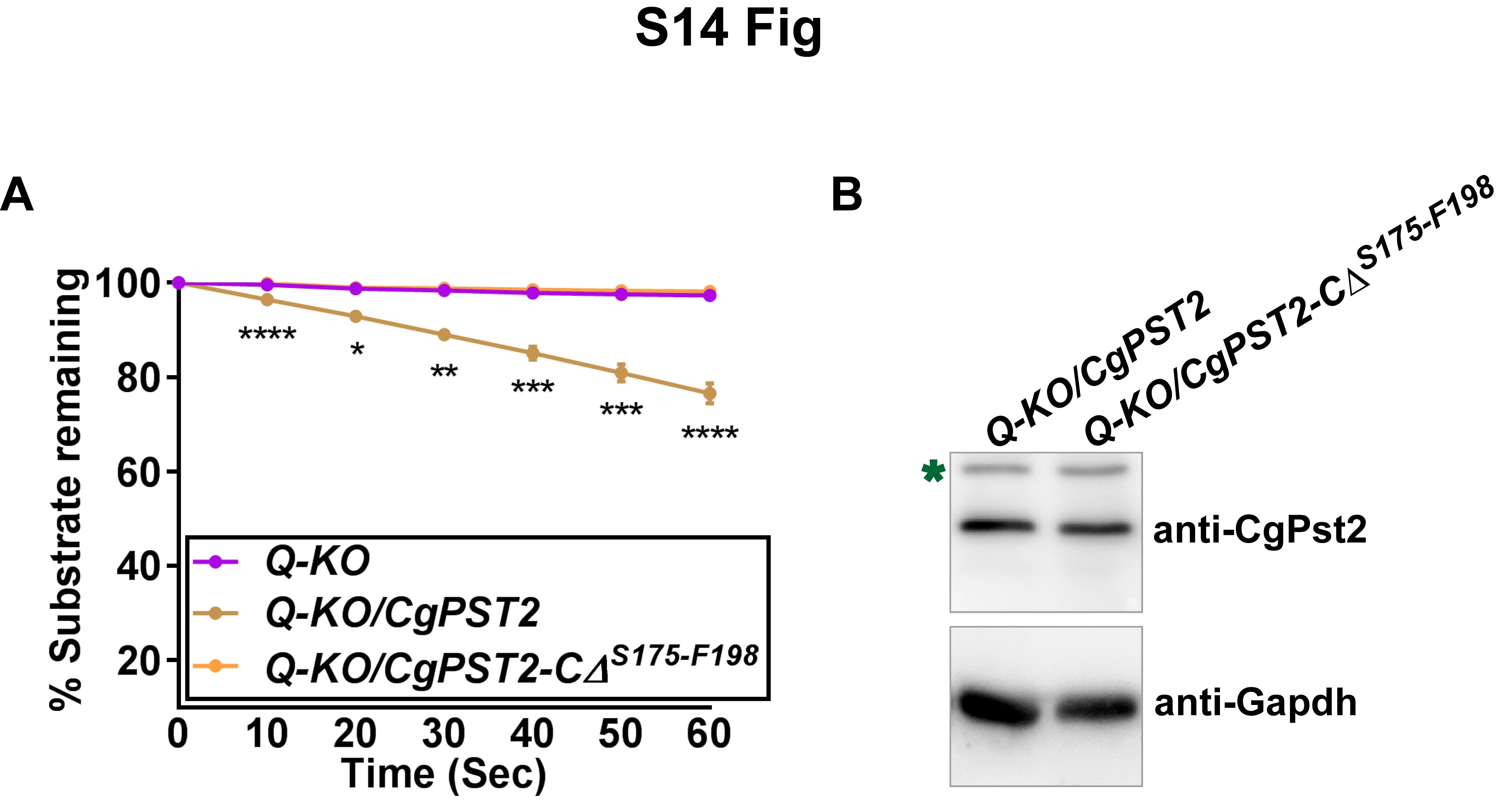

Supplement: S14 Fig — A. NADH:quinone oxidoreductase activity measurement in extracts of Q-KO expressing either CgPST2 or CgPST2-CΔS175-F198. Data represent mean ± SEM. Grouped multiple t-test was performed, with n = 3 to 4. *, p < 0.0332; **, p < 0.0021; ***, p < 0.0002; ****, p < 0.0001. The Q-KO strain lacks four flavodoxin-like proteins, CgPst2, CgRfs1, CgPst3 and CgYcp4. B. Immunoblot analysis of CgPst2 levels in Q-KO strains expressing CgPST2 or CgPST2-CΔS175-F198. The green asterisk denotes non-specific band. (TIF) [file ppat.1009355.s014.tif]

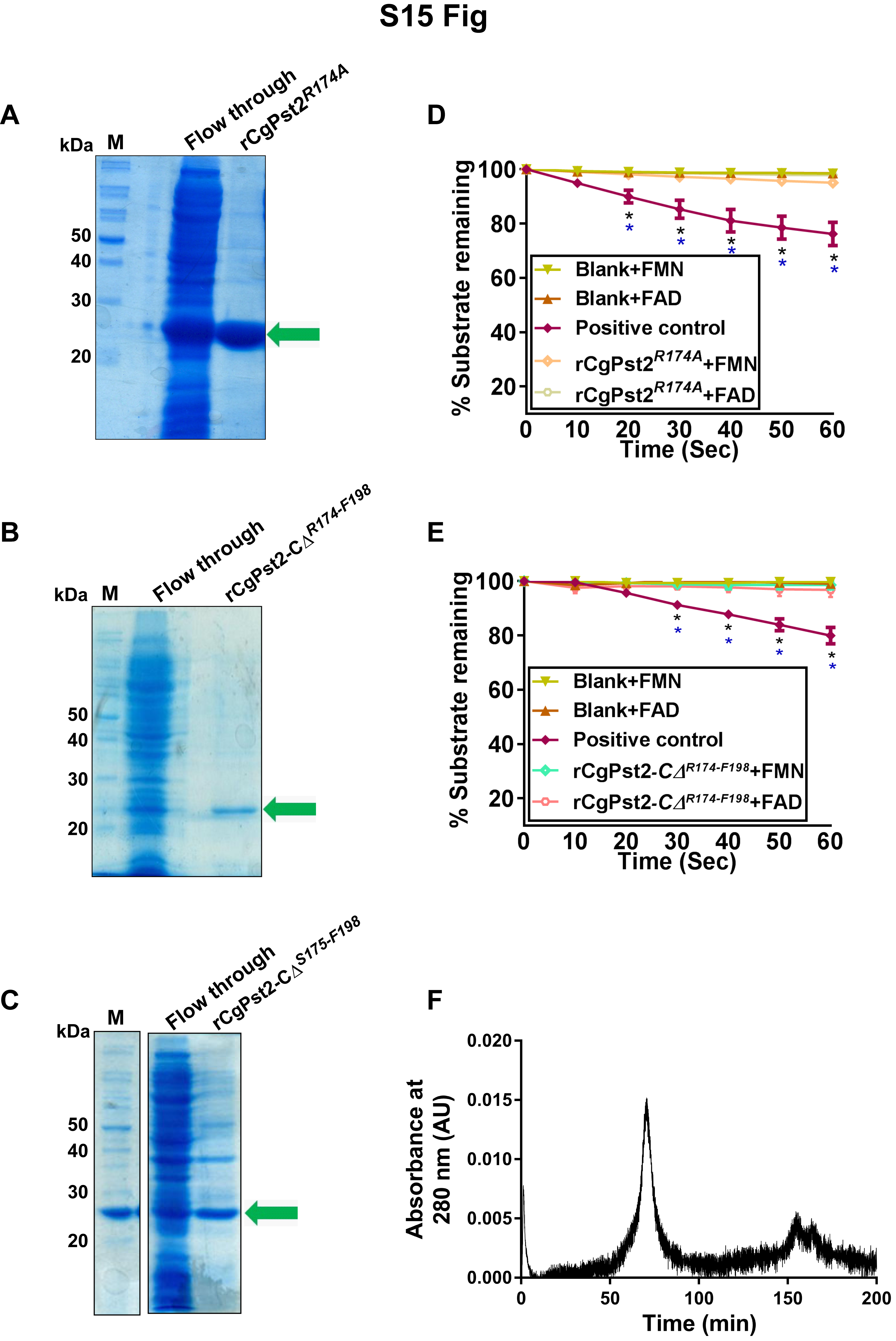

Supplement: S15 Fig — A-C. 6X-Histidine-FLAG-CgPst2R174A A., 6X-Histidine-FLAG-CgPst2-CΔR174-F198 B. and 6X-Histidine-FLAG-CgPst2-CΔS175-F198 C. protein purification from E. coli. The E. coli BL21 (DE3) strain transformants carrying pET28a(+)-6XHIS-FLAG-CgPST2R174A, pET28a(+)-6XHIS-FLAG-CgPST2-CΔR174-F198 and pET28a(+)-6XHIS-FLAG-CgPST2-CΔS175-F198 plasmids were grown in LB medium, induced with IPTG (0.5 mM) at 18οC for 16 h, and cells were collected. After cell lysis, the recombinant proteins were purified using TALON metal affinity resin via affinity purification. 30 μl eluates, representing purified rCgPst2R174A A. rCgPst2-CΔR174-F198 B. and rCgPst2-CΔS175-F198 C. proteins, along with the flow through, were resolved on 12% SDS-PAGE, and stained with Coomassie Brilliant Blue. The green arrow marks CgPst2 band. M, Protein Marker. D-E. NADH:quinone oxidoreductase activity measurement of the recombinant CgPst2R174A D. and CgPst2-CΔR174-F198 E. protein (250 μg) was measured, as described in the legend of S3B Fig. Blank contained no protein. Data represent mean ± SEM. Grouped multiple t-test was performed, with n = 3. Black and blue asterisks indicate statistically significant activity differences between the positive control [NAD(P)H:FMN oxidoreductase (1 Unit, Roche, # 10476480001)] and recombinant proteins incubated with FMN (100 μM) and FAD (100 μM), respectively. *, p < 0.0332. Please note that the enzymatic activity of the purified recombinant CgPst2-CΔS175-F198 protein could not be determined, as it formed precipitates during the assay reaction. F. Size exclusion chromatogram of CgPst2-CΔS175-F198. After loading 300 μg of purified 6XHIS-FLAG-CgPst2-CΔS175-F198 protein on the Sephacryl S-200 column, protein elution profiles were determined using the absorbance values at 280 nm. The CgPst2-CΔS175-F198 protein could not bind to the column, and appeared as aggregates in the column’s void volume, as determined by column calibration with blue dextran. AU, Arbitrary Units. (TIF) [file ppat.1009355.s015.tif]

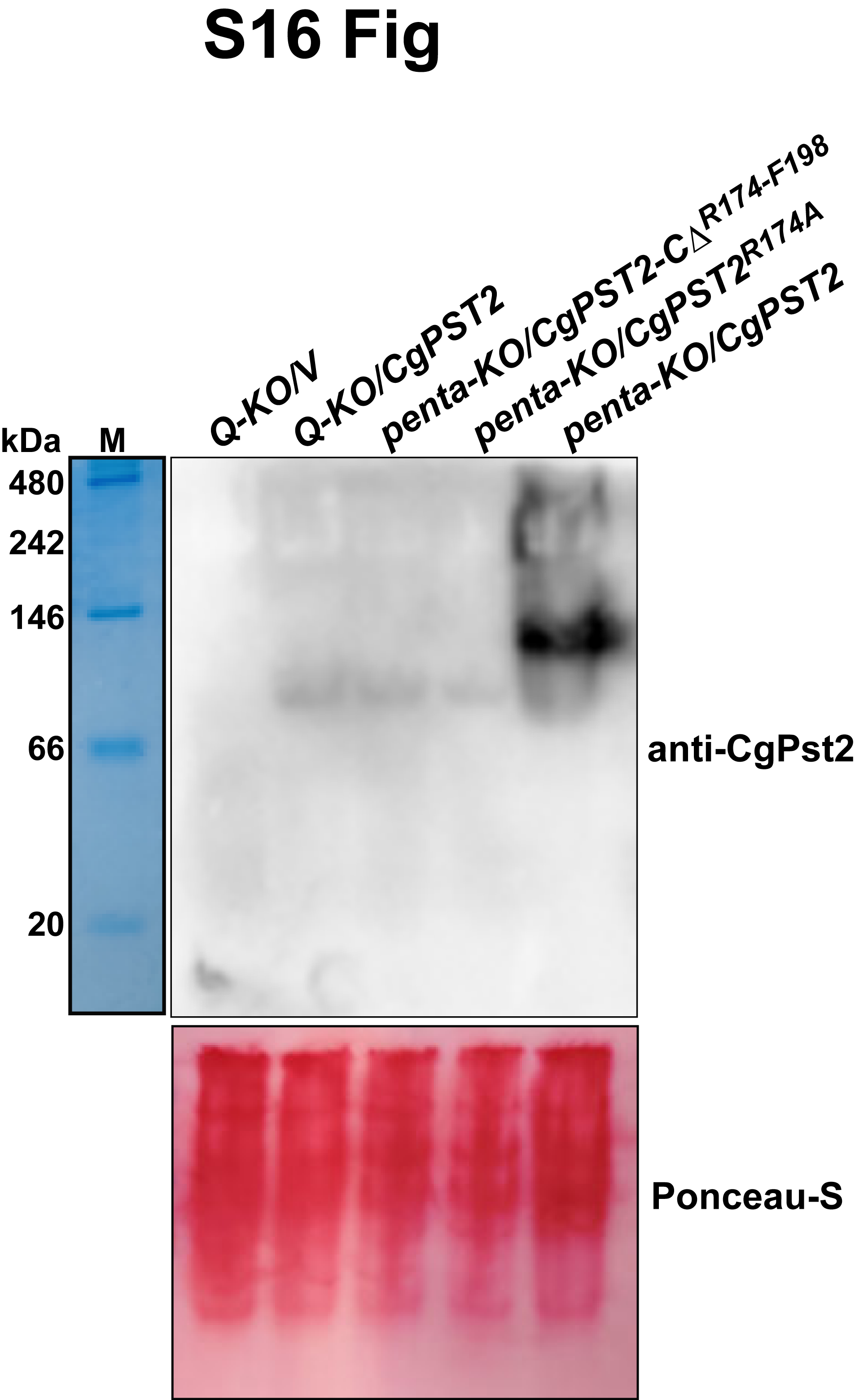

Supplement: S16 Fig — 300 μg whole cell lysates of Q-KO- expressing vector pRK74 (V) or CgPST2, and penta-KO expressing CgPST2-CΔR174-F198, CgPST2R174A and CgPST2 were resolved in a discontinuous Tris-glycine buffer system under non-denaturing conditions, and probed with anti-CgPst2 antibody. The native protein molecular weight marker (M) was stained with coomassie brilliant blue, and is shown on the right side of the blot. The ponceau S-stained membrane is shown as loading control. (TIF) [file ppat.1009355.s016.tif]

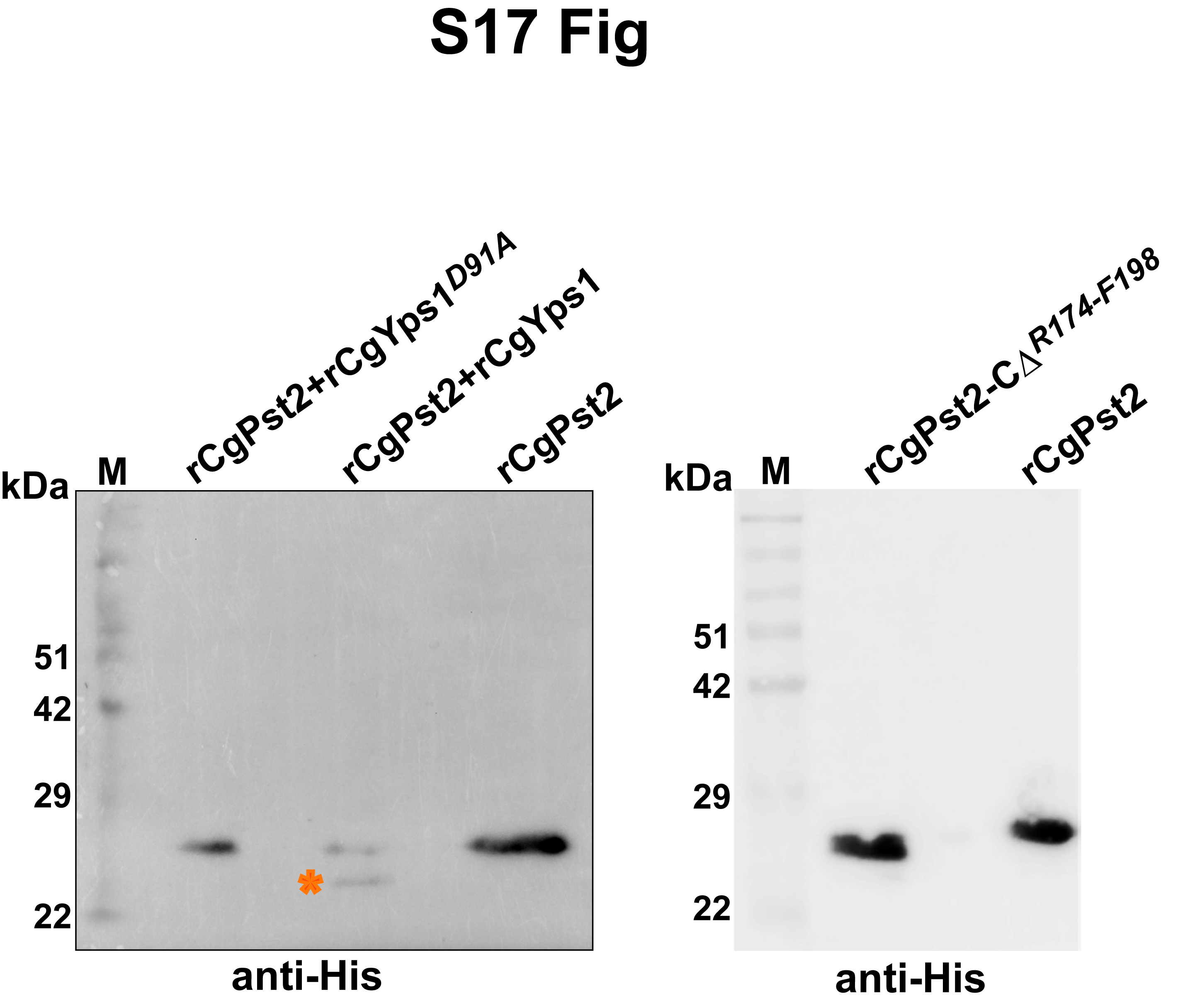

Supplement: S17 Fig — In vitro cleavage assay. The Pichia pastoris GS115 strain expressing either CgYps1 or CgYps1D91A was induced with 2% methanol for 48 h, and pelleted down at 14000 rpm for 10 min at 4°C. The supernatants containing CgYps1 and CgYps1D91A were filtered through a 0.22 μM filter (Millipore, USA), and concentrated using Amicon Ultra centrifugal filter unit (3 kDa cutoff). The retentate was precipitated with ammonium sulphate (100% saturation) for 10 min at 4°C, followed by centrifugation and pellet suspension in citrate buffer (pH 4.0). For the cleavage assay, 30 μg of r6XHis-Flag-CgPst2 was incubated with 60 μg of partially purified rCgYps1 or rCgYps1D91A enzymes at 37°C for 4 h in citrate buffer (pH 4.0). Digested samples were run on 18% SDS-PAGE and probed with anti-His antibody. The assay samples containing both CgYps1 and CgPst2 displayed an additional faster migrating CgPst2 protein band (~ 24 kDa; marked with orange asterisk), probably representing the N-terminal fragment of the cleaved form of CgPst2, which was absent in reaction mixtures containing either CgPst2 alone or both CgPst2 and CgYps1D91A. These data indicate that the catalytically active CgYps1 can cleave CgPst2. Of note, we could not detect the small C-terminal-cleaved CgPst2 fragment, as hexa-His epitope is present at the N-terminus. To estimate the size difference between the cleaved and un-cleaved form of CgPst2, E-coli-purified full-length CgPst2 and CgPst2-CΔR174-F198 (50 μg) were run on 18% SDS-PAGE as a control, and probed with anti-his antibody. M, Protein Marker. (TIF) [file ppat.1009355.s017.tif]
