# Supplementary material for: An aspartyl protease-mediated cleavage regulates structure and function of a flavodoxin-like protein and aids oxidative stress survival
Source: PLoS Pathog. 2021 Feb 25;17(2):e1009355. doi: 10.1371/journal.ppat.1009355 (PMC7943015; doi:10.1371/journal.ppat.1009355)
Supplement: S1 Text — (DOCX) [file ppat.1009355.s024.docx]

**S1 Text: Supporting protocols**

**Serial dilution spotting assay**

*C. glabrata* cells were grown overnight at 30°C and cell suspensions in PBS corresponding to 2.0 OD_600_ were prepared. Cultures were 10-fold serially diluted and 3 μl of each dilution was spotted on different media. Plate images were captured after 1-4 days of incubation at 30°C.

**Glutathione estimation**

Extracts of log phase cells (10.0 OD_600_) were prepared and suspended in 500 μL of cold metaphosphoric acid (5% w/v). Cells were diluted 20 and 30 times with 1X assay buffer (supplied with the kit) for the estimation of oxidized and total glutathione, respectively. Total glutathione (50 μl sample) was measured by recording absorbance at 405 nm at 1 min interval over a duration of 10 min in the MULTISKAN GO microplate spectrophotometer (Thermo Scientific), using the Glutathione (GSSG/GSH) detection kit (Enzo Life Sciences #ADI-900-160), as per manufacturer’s instructions. For oxidized glutathione measurement, 50 μl sample was incubated with GSSG (4 µM) and 4-vinylpyridine (2 M) at room temperature for 1 h. The standard glutathione curve was prepared using GSSG. The ratio of GSSG/GSH was deduced from GSSG/(total GSH - GSSG).

**Expression and purification of CgYps1 and CgYps1*^D91A^* in *Pichia pastoris***

The CgYps1 and CgYps1*^D91A^*-encoding *CgYPS1* (*CAGL0M04191g*) ORFs without the C-terminal GPI-anchor site-encoding sequence were cloned in AvrII and NotI sites in the expression plasmid pPIC-9 carrying the PHO secretion signal sequence (Pichia Expression kit #K1710-01, Life Technologies) in such a manner that the expressed proteins are tagged with the 6X-Histidine epitope at the C-terminus. The plasmids were linearized with StuI and transformed into the *Pichia pastoris* GS115 strain through electroporation, followed by selection for histidine prototrophy. The integration at *HIS4* locus in the genome was confirmed by PCR. For purification, *Pichia* cells were grown in the BMGY (Buffered Glycerol Complex Medium) medium at 30°C for 24 h and suspended in the BMMY medium containing 2% methanol to induce the *AOX1* promoter. After 48 h growth at 30^ο^C, as CgYps1 proteins were secreted out, the culture media was collected, filtered through a 0.22 µM filter (Millipore, USA), followed by concentration using a 3 kDa Amicon Ultra Centrifugal Filter Unit. For anion exchange chromatography-based purification, the media was loaded on activated DEAE cellulose resin (HiMedia; 10 ml Bed volume), and proteins bounds to resin were washed with the citrate buffer (10 mM; pH 6.5), followed by elution with NaCl (150 mM)-containing citrate buffer (100 mM; pH 4.0). As CgYps1 protein was found to precipitate at neutral pH, purification was done at acidic pH, where it also shows maximal activity. Eluates were dialyzed for 4-6 h at 4°C in citrate buffer [100 mM (pH 4.0) and 20% PEG], and approximately 1-2 mg of CgYps1 and CgYps1*^D91A^* was obtained. The proteolytic activity of purified rCgYps1 and rCgYps1*^D91A^* was determined using gelatin and hemoglobin as substrates. For CgPst2 cleavage assay, rCgYps1 and rCgYps1*^D91A^* (60 µg) were incubated with CgPst2 (30 µg) for 4 h at 37℃ in the citrate buffer (pH 4.0). Digested samples were run on 18% SDS-PAGE and probed with anti-His antibody.

**Confocal Microscopy**

*C. glabrata* strains expressing CgPst2-GFP from the *pCU-PDC1-GFP* (Addgene #45324) plasmid were grown to log-phase in CAA medium, washed in PBS, and visualized under the confocal microscope (Zeiss LSM 700; 63X/1.44 NA objective or Leica TCS SP8; 63X/1.52NA objective).

**Quantitative Real-time PCR**

*CgPST2* transcript levels were measured in log-phase cultures of *T-KO* and *T-KOyps1Δ* strains. Total RNA was isolated using the hot phenol-extraction method, and the real-time quantitative reverse transcriptase PCR reaction was set up with 500 ng RNA using the SuperScript III First-Strand Synthesis System [Thermo Fisher Scientific; #18080051]. Specificity of primers was first checked using appropriate *C. glabrata* mutant strains. *CgPST2* transcript levels were normalized against the *CgACT1* mRNA control, and quantified using the 2^ΔΔ^C_t_ method.

**Antibody generation**

For generating anti-CgPst2 polyclonal antibody, BALB/c mice were injected subcutaneously on multiple sites with CgPst2 (100 µg) and Freund’s Complete Adjuvant (1:1) emulsion, followed by the first and the second booster injection after three and six weeks, respectively. The test and final bleed were collected from the retro-orbital plexus in anaesthetized and sacrificed mice. The specificity of anti-CgPst2 sera was checked using appropriate *CgPST2*-deleted strains. The polyclonal antibody against N-terminally 6XHis-tagged CgYps1 protein, devoid of signal peptide and propeptide regions, was raised in BALB/c mice, as described previously [Rasheed *et al.* 2020].

**Reference**

1. Rasheed M, Kumar N, Kaur R. Global secretome characterization of the pathogenic yeast *Candida glabrata*. J Proteome Res*.* 2020;**19**: 49–63.doi:10.1021/acs.jproteome.9b00299
